# A New *N*-methoxypyridone from the Co-Cultivation of Hawaiian Endophytic Fungi *Camporesia sambuci* FT1061 and *Epicoccum sorghinum* FT1062

**DOI:** 10.3390/molecules22071166

**Published:** 2017-07-12

**Authors:** Chunshun Li, Ariel M. Sarotti, Baojun Yang, James Turkson, Shugeng Cao

**Affiliations:** 1 Department of Pharmaceutical Sciences, Daniel K. Inouye College of Pharmacy, University of Hawai’i at Hilo, 200 West Kawili Street, Hilo, HI 96720, USA; chunshun@hawaii.edu; 2 Cancer Biology Program, University of Hawaii Cancer Center, 701 Ilalo Street, Honolulu, HI 96813, USA; byang@cc.hawaii.edu (B.Y.); jturkson@cc.hawaii.edu (J.T.); 3 Instituto de Química Rosario (CONICET), Facultad de Ciencias Bioquímicas y Farmacéuticas, Universidad Nacional de Rosario, Suipacha 531, Rosario 2000, Argentina; sarotti@iquir-conicet.gov.ar

**Keywords:** endophytic fungi, co-culture, pyridone, tetramic acid, Hawaii

## Abstract

A new *N*-methoxypyridone analog (**1**), together with four known compounds, was isolated from the co-culture of Hawaiian endophytic fungi *Camporesia sambuci* FT1061 and *Epicoccum sorghinum* FT1062. The structure of the new compound was elucidated as 11*S*-hydroxy-1-methoxyfusaricide (**1**) by extensive spectroscopic analysis and comparison with the literature. The absolute configuration of **1** was determined by comparison with the experimental and calculated ECD spectra. The absolute configuration of compound **3** was investigated and renamed as (+)-epipyridone by comparison of the optical rotation and CD spectrum with those of **1**. The other known compounds were identified as epicoccarine B (**2**), D8646-2-6 (**4**), and iso-D8646-2-6 (**5**). Compounds **4** and **5** showed modest inhibitory activity towards pathogenic fungi. Epicoccarine B (**2**) inhibited A2780 and TK-10 with an IC_50_ value of 22 μM.

## 1. Introduction

Manipulation of fermentation conditions of fungi has been proven to be an efficient strategy for obtaining diversified compounds [1,2,3]. Co-cultivation of two or more strains has recently been described as a promising strategy for inducing the production of bioactive microbial metabolites [4,5]. In addition to obtaining new metabolites [6,7,8,9], the strategy can also be used to increase the yields of previously described bioactive compounds [10].

**Figure 1:**
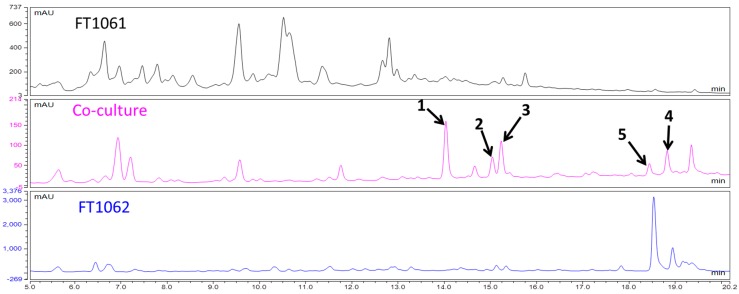
HPLC chromatograms of the EtOAc extracts from co-culture and single cultures of FT1061 and FT1062. (Peaks of compounds **1**–**5** in the chromatography of the extract from co-culture were marked).

**Figure 2:**
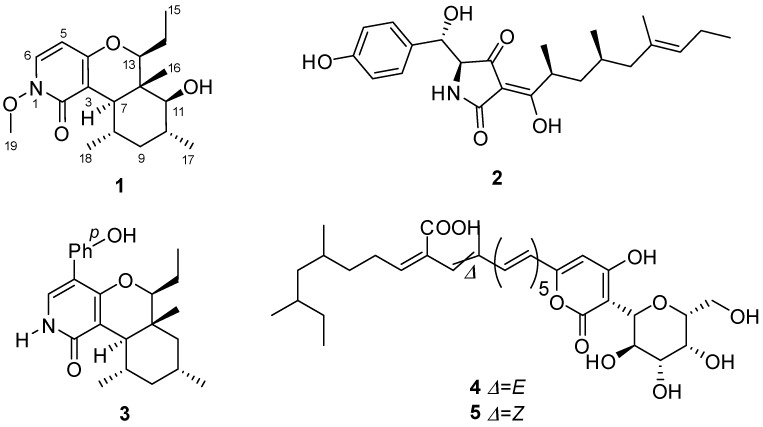
Structures of compounds **1***–***5**.

Endophytic fungi living within plants are well known for the production of therapeutically interesting compounds [11,12,13,14]. During our continuing investigation of Hawaiian endophytic fungi [15,16,17,18,19,20,21,22], two endophytic fungal strains, FT1061 (*Camporesia sambuci*) and FT1062 (*Epicoccum sorghinum*) were isolated from *Rhodomyrtus tomentosa* (downy rose myrtle) [23], an invasive pest plant in the State of Hawaii. *Camporesia sambuci* has rarely been investigated, while *Epicoccum sorghinum* is a facultative plant pathogen that is associated with grain mold of sorghum and other crops, and which produces the mycotoxin tenuazonic acid [24]. We noticed that the co-cultivation of FT1061 and FT1062 produced some compounds that were not obviously observed in the culture of either FT1061 or FT1062 alone (Figure 1). We argue that some silent genes of these two strains are activated under competitive stress, thus producing more defensive secondary metabolites. LC/MS-guided separation from the co-cultured broth led to the isolation and identification of a new *N*-methoxypyridone analog (**1**), and four known compounds: epicoccarine B (**2**), (+)-epipyridone (**3**), D8646-2-6 (**4**), and iso-D8646-2-6 (**5**) (Figure 2). The aims of this study were to characterize these compounds and to evaluate their anti-microbial and anti-cancer activity.

## 2. Results and Discussion

**Figure 3:**
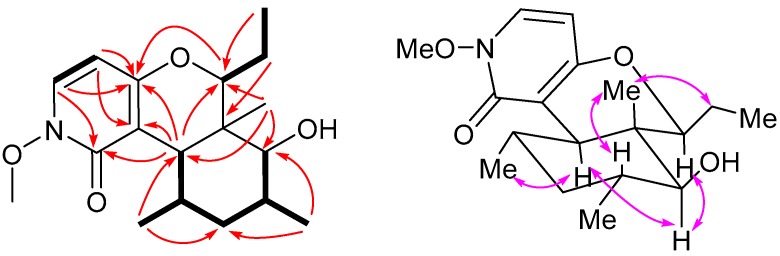
Key ^1^H-^1^H COSY (bold), HMBC (red single-headed arrows) and NOESY (pink double-headed arrows) of **1**.

11*S*-Hydroxy-1-methoxyfusaricide (**1**) was isolated as a colorless gum. Its molecular formula was determined to be C_18_H_27_NO_4_ by HR-ESIMS (*m*/*z* 322.2017, calcd for [M + H]^+^ 322.2013), with six degrees of unsaturation. The IR spectrum (supplementary materials Figure S9) showed the existence of hydroxyl (3394 cm^−1^) and amide (1640 cm^−1^) groups. A detailed analysis of 1D and 2D NMR spectra (Table 1) demonstrated the presence of five methyls, including one methoxy group; two methylenes; seven methines, including two oxygenated and two olefinic ones; and four carbons with no hydrogen attached including a carbonyl carbon. The ^1^H-^1^H COSY spectrum of **1** indicated three spin systems: C5–C6, C7–C8(-C18)–C9–C10(-C17)–C11, and C13–C15, which were also verified by the corresponding HMBC correlations (Figure 3). Meanwhile, the HMBC correlations from the methyl group CH_3_-16 to C-7, C-11, C-12 and C-13, and from H-7 to C-2 (δ_C_ 159.4), C-3 (δ_C_ 114.6), and C-4 (δ_C_ 165.8), as well as a correlation from H-13 to the oxygenated olefinic carbon C-4, suggested that compound **1** should be an analog of fusaricide [25,26]. However, one oxygenated methine (instead of a methylene) at 11-position and one methoxy group at 1-position were observed in **1**. The HMBC correlations from the methyl groups CH_3_-17 (δ_H_ 1.07) and CH_3_-16 (δ_H_ 1.12) to the oxgenated methine (δ_C_ 84.7) suggested that C-11 was substituted by a hydroxy group, which was also consistent with the molecular formula. Since no HMBC was observed between the methyoxy group with any other carbon, the position of the methyoxy was assigned to be connected to the nitrogen atom. The methyoxy (δ_C_ 65.0) was de-shielded [27], which also supported this deduction.

The relative configuration of the molecule was determined by the analysis of the NOESY spectrum. The correlations from H-11 to H-13 and H-7 implied their co-facial orientation, which was assigned as α. The observed NOE cross-peak between Ha-14 and H_3_-16 indicated that both were on the β orientation. Meanwhile, the correlations between H-10 and H_3_-16, and between H-7 and H_3_-18, suggested that the orientations of two methyl groups CH_3_-17 and CH_3_-18 were α. Hence, the relative configuration of compound **1** was determined as shown in Figure 3.

**Figure 4:**
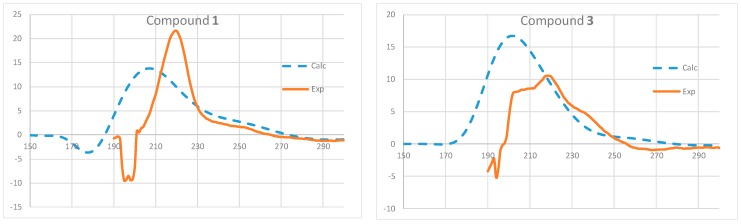
Experimental and calculated CD spectra of compounds **1** and **3**.

The absolute configuration of compound **1** was determined by comparing its optical rotation with those of (**3**) [28], cordypyridone C (**6**) [27], 14-hydroxycordypyridone C (**7**) [27] and fusaricide (**8**) [25,26,27], and its CD with that of compound **3**. The absolute configuration of the *p*-bromobenzoate of compound **7** was established using anomalous scattering X-ray crystallographic methods [27]. By comparison of the spectral data and optical rotations (**1**: ${\left[\alpha \right]}_{D}^{25}$ + 104.8, *c* 0.02, MeOH; **3**: ${\left[\alpha \right]}_{D}^{25}$ + 123.3, *c* 0.06, MeOH; **6**: ${\left[\alpha \right]}_{D}^{24}$ + 243, *c* 0.06, MeOH; **7**: ${\left[\alpha \right]}_{D}^{27}$ + 152, *c* 0.15, MeOH; **8**: ${\left[\alpha \right]}_{D}$ + 194, *c* 0.12, CHCl_3_), we believe that all five compounds should have the same absolute configuration. The structure of compound **8** was redrawn as shown in reference [27]. The absolute configuration of (+)-epipyridone should be drwan as **3** rather than its enanthiomer [28]. The CD spectrum of **1** was similar to that of compound **3** (Figure 4), also indicating that both must have the same absolute configuration, which was consistent with a biogenetic point of view. Hence, compound **1** was determined as 11*S*-hydroxy-1-methoxyfusaricide, which could also be named as 11*S*-hydroxy-14-methyl cordypyridone C. In order to confirm the absolute configuration suggested for **1** and **3**, we next carried out ECD calculations using time-dependent density functional theory (TDDFT) at the B3LYP/6-31G* level. As shown in Figure 4, a good correlation between experimental and calculated data was found. Despite the fact that the computed maximum absorption bands are slightly shifted toward the low wavelength region, the collected results are completely congruent with the configurational analysis discussed above. 

Compounds **2**−**5** were identified to be epicoccarine B [28], (+)-epipyridone [28], D8646-2-6 [29], and iso-D8646-2-6 [29], respectively, by comparison of the NMR data with those reported in the literature. The anti-microbial activities of the isolated compounds were evaluated against four bacterial strains *Escherichia coli*, *Pseudomonas aeruginosa*, *Staphylococcus aureus*, and *Bacillus subtilis*, and four pathogenic fungal strains *Pennicillium chrysogenum*, *Aspergillus niger*, *Paecilomyces lilacinus*, and *Fusarium graminearum*. Compounds **4** and **5** showed weak activity against *A. niger* and *P. lilacinus* with the MIC values of 32 μg/mL, respectively. Compound **2** exhibited anti-proliferative activity against the human ovarian cancer cell line A2780 and the human kidney renal cell adenocarcinoma TK-10 with an IC_50_ value of 22 μM.

## 3. Materials and Methods

### 3.1. General Experimental Procedures

Optical rotation was measured with a Rudolph Research Analytical AutoPol IV Automatic Polarimeter. UV and IR spectra were obtained with Shimadzu UV-1800 spectrophotometer (Shimadzu, Kyoto, Japan) and Thermo scientific Nicolet iS50FT-IR spectrometer (Thermo Fisher Scientific, Waltham, MA, USA), respectively. CD spectra were recorded on Jasco J-815 circular dichroism spectrophotometer (Jasco Products Company, Oklahoma City, OK, USA) in methanol at the concentration of 0.01 mg/mL (the length of the cell path was 1 cm). NMR spectra including 1D and 2D experiments were recorded on a Bruker 400 MHz NMR; HPLC was carried out on Thermo scientific Ultimate 3000 LC system using a Phenomenex Luna phenyl-hexyl column (100 mm × 21.2 mm, 5 μm particle size, Phenomenex, Torrance, CA, USA) and a Phenomenex Luna C18 HPLC column (250 mm × 10 mm, 5 μm particle size, Phenomenex, Torrance, CA, USA). All solvents were HPLC grade. Column chromatography was performed using Diaion HP-20 (Sigma, St. Louis, MO, USA).

### 3.2. Isolation and Identification of Fungal Strain

The fungal strains *Camporesia sambuci* FT1061 and *Epicoccum sorghinum* FT1062 were isolated on PDA medium from a healthy fruit of the plant Rhodomyrtus tomentosa collected on the Big Island in Hawaii in 2016. The fungal strains have been deposited at the strain bank of Daniel K. Inouye College of Pharmacy, University of Hawai’i at Hilo. Mycelia were retrieved by filtration and ground to a fine powder in liquid N_2_. Genomic DNA was extracted using the SurePrep RNA/DNA/protein purification kit (Fisher Bioreagents, Waltham, MA, USA), and large subunit rDNA was amplified by PCR using primers LROR and LR5. PCR products were sequenced at Genewiz (http://www.genewiz.com/). The DNA sequence data obtained from the fungal strains FT1061 and FT1062 have been deposited at GenBank with accession number KY971273 and KY971274, respectively.

### 3.3. Cultivation

The two fungal strains FT1061 and FT1062 were inoculated together and grown under static conditions at room temperature for 30 days in one 1 L conical flask containing the liquid medium (300 mL/flask) composed of mannitol (20 g), sucrose (10 g), monosodium glutamate (5 g), KH_2_PO_4_ (0.5 g), MgSO_4_·7H_2_O (0.3 g), yeast extract (3 g), corn steep liquor (2 mL), in 1 L distilled water; pH 6.5 prior sterilization.

### 3.4. Isolation of Compounds ***1***–***5***

The whole fermented broth (4.5 L) was filtered through filter paper to separate the supernatant from the mycelia. The filtered supernatant was passed through a HP-20 column (Diaion, Sigma, St. Louis, MO, USA), eluted with MeOH-H_2_O (10%, 40%, 70%, 90% and 100% methanol in H_2_O) to afford five fractions (Fr. A‒E). Fraction C (517.8 mg) was separated by a preparative HPLC column (C18 column, 5 µm, 100.0 mm × 21.2 mm; 10 mL/min; 10–100% methanol in H_2_O in 40 min) to generate 40 sub-fractions (C1‒40). C35 (27.4 mg) was subjected to the semi-preparative HPLC (C18 column, 5 µm, 250.0 mm × 10.0 mm; 4 mL/min; with 0.1% formic acid in 75% methanol in H_2_O) to obtain compounds **4** (7.12 mg, *t*_R_ 31.5 min) and **5** (1.56 mg, *t*_R_ 33.5 min). Fraction D (347.2 mg) was separated with a preparative HPLC column (C18 column, 5 µm, 100.0 mm × 21.2 mm; 10 mL/min; 30–100% methanol in H_2_O in 30 min) to generate 30 sub-fractions (D1‒30). D20 (8.47 mg) was subjected to the semi-preparative HPLC (C18 column, 5 µm, 250.0 mm × 10.0 mm; 3 mL/min; with 0.1% formic acid in 58% methanol in H_2_O) to afford compound **1** (1.34 mg, *t*_R_ 35.0 min). D26 (18.28 mg) was subjected to the semi-preparative HPLC (C18 column, 5 µm, 250.0 mm × 10.0 mm; 3 mL/min; with 0.1% formic acid in 75% methanol in H_2_O) to afford compounds **2** (8.51 mg, *t*_R_ 20.8 min) and **3** (1.38 mg, *t*_R_ 25.6 min).

### 3.5. Charaterization of Compound ***1***

11*S*-Hydroxy-1-methoxyfusaricide (**1**), Colorless solid; ${\left[\alpha \right]}_{D}^{25}$ + 104.8 (*c* = 0.02, MeOH); UV (MeOH) λ_max_ (log ε) 202 (3.63), 260 (3.64) nm; IR ν_max_ 3394, 2936, 2831, 1640, 1593, 1542, 1455, 1356, 1277, 1234, 1203, 1177 cm^−1^; ^1^H (in methanol-*d*_6_, and in CDCl_3_ at 400 MHz) and ^13^C-NMR (in methanol-d_4_, 100 MHz) data, see Table 1; positive HR-ESIMS *m*/*z* 322.2017 [M + H]^+^ (calcd. for C_18_H_28_NO_4_ 322.2013).

### 3.6. Anti-Microbial Activity

The isolated compounds were tested for their inhibitory activities against four bacteria, *E. coli*, *P*. *aeruginosa*, *S. aureus*, and *B. subtilis*, and five fungi *C. sambuci* (FT1061), *P. chrysogenum*, *A. niger*, *P. lilacinus*, and *F. graminearum* by the broth-microdilution method [30]. Chloramphenicol was used as the positive control.

### 3.7. Anti-Proliferative Activity

Viability of TK-10, A2780 and A2780CisR, was determined using the CyQuant cell proliferation assay kit, according to the manufacturer’s instructions (Life Technologies, Camarillo, CA, USA) [31]. Briefly, cells were cultured in 96-well plates at 6000 cells per well for 24 h and subsequently treated with compounds (20 μg/mL) for 72 h and analyzed. Relative viability of the treated cells was normalized to the DMSO-treated control cells [31,32]. Cisplatin was used as a positvie control.

### 3.8. ECD Calculations

Initial systematic conformational searches of compounds **1** and **3** were carried out at the MMFF level using Spartan 08 [33]. Further full geometry optimizations of all conformers found were done at the B3LYP/6-31G* level of theory. The excitation energies (nm) and rotatory strength (*R*) in dipole velocity (*R_vel_*) of the first twenty singlet excitations were calculated using TDDFT at the B3LYP/6-31G* level from all significantly populated conformers, which were then averaged using Boltzmann weighting [34]. The calculated rotatory strengths were simulated into the ECD curve as the sum of gaussians with 0.5 eV width at half-heights (σ). All DFT calculations were carried out using Gaussian 09 [35].

## 4. Conclusions

Microbial communication can lead to the activation of silent fungal secondary metabolite gene clusters [36], which has been proved to be a potentail way to enhance chemical divesity [37]. Some co-cultivations were conducted between fungi [6,7], but some between fungi and bacteria [8,9]. Co-cultivation of two endophytic fungi FT1061 (*Camporesia sambuci*) and FT1062 (*Epicoccum sorghinum*) led to the identification of a new *N*-methoxypyridone analog, 11*S*-hydroxy-1-methoxyfusaricide (**1**), which was not produced by FT1061 or FT1062 alone. LC-MS investigation suggested that compounds **2**–**5** were detected in the single cultured broth of FT1062 but not FT1061. The structural similarity of compounds **1** and **3** implied that compound **1** should also be produced by FT1062, but it was not detected in the single cultured broth of FT1062, probably due to low yield or a silenced gene. LC-MS data also indicated that most of the major metabolites in the single cultured broth of FT1061 are small molecules with molecular weights in the range of 100–200 Da, so we didn’t pursue them. A number of compounds normally produced by the strain FT1061 were missing in the co-culture. We assume that the genes accounting for the production of these small molecules in FT1061 were silenced when co-cultured with FT1062. Compounds **1**–**5** were evaluated for their anti-bacterial, anti-fungal and anti-proliferative activities. Compounds **4** and **5** showed moderate inhibitory activities against two fungal strains *A. niger* and *P. lilacinus*. Compound **2** exhibited moderate inhibition against the human ovarian cancer cell line A2780 and the human kidney spindle cell carcinoma cell line TK-10.

## Figures and Tables

**Table 1:** ^1^H- (400 MHz) and ^13^C-NMR (100 MHz) spectroscopic data for compound **1**.

No.	1 in Methanol-*d*_4_			1 in CDCl_3_
	δ_H_, *J* (Hz)	δ_C_ *^a^*	HMBC Correlation	δ_H_, *J* (Hz)
2		159.4		
3		114.6		
4		165.8		
5	6.03, d, 7.6	101.5	C-3, C-4	5.84, d, 7.6
6	7.73, dd, 7.6, 0.8	136.6	C-2, C-4	7.37, dd, 7.6, 0.8
7	2.07, d, 11.6	50.3	C-2, C-3, C-4, C-12,	2.04, d, 11.6
			C-8, C-9, C-13, C-16	
8	2.69, m	27.3		2.75, m
9a	1.84, dt, 13.5, 4.0	44.2	C-7, C-11, C-8, C-10, C-17	1.82, dt, 13.5, 4.0
9b	0.85, br.d, 13.5		C-7, C-11, C-8, C-10, C-17, C-18	0.82, br.d, 13.5
10	1.69, m	33.7		1.66, m
11	3.03, d, 10.4	84.7	C-9, C-10, C-12, C-13, C-16, C-17	3.08, d, 10.3
12		45.8		
13	4.10, dd, 10.8, 1.6	94.9	C-4, C-11, C-12, C-14, C-15, C-16	4.06, dd, 10.9, 1.7
14a	2.05, m	27.4	C-12, C-13, C-15	2.00, m
14b	1.27, m		C-13, C-15	1.25, m
15	1.12, t, 7.4	11.5	C-13, C-14	1.08, t, 7.3
16	0.71, s	9.8	C-7, C-11, C-12, C-13	0.73, s
17	0.99, d, 6.3	18.7	C-9, C-10, C-11	0.97, d, 6.4
18	1.07, d, 6.0	23.7	C-7, C-8, C-9	1.08, d, 5.9
19	3.98, s	65.0		4.01, s

*^a^* Data of ^13^C were obtained by HSQC and HMBC spectra.

## Supplementary Materials

The following are available online: NMR, HRESIMS, IR spectra of compound **1**, and ECD calculation information of compounds **1** and **3** as supporting information.
