# Supplementary material for: A New N-methoxypyridone from the Co-Cultivation of Hawaiian Endophytic Fungi Camporesia sambuci FT1061 and Epicoccum sorghinum FT1062
Source: Molecules. 2017 Jul 12;22(7):1166. doi: 10.3390/molecules22071166 (PMC6152147; doi:10.3390/molecules22071166)
Supplement: Supplementary file 1 [file molecules-22-01166-s001.pdf]

Supporting Information

A New N-methoxypyridone from the Co-cultivation of Hawaiian Endophytic Fungi *Camporesia sambuci* FT1061 and *Epicoccum sorghinum* FT1062

Chunshun Li[1,2], Ariel M. Sarotti[3], Baojun Yang[2], James Turkson[2] and Shugeng Cao[1,2,*]

[1] Department of Pharmaceutical Sciences, Daniel K. Inouye College of Pharmacy, University of Hawai'i at Hilo, 200 West Kawili Street, Hilo, HI 96720, USA; chunshun@hawaii.edu (C.L.); scao@hawaii.edu (S. C.)

[2] Cancer Biology Program, Cancer Center, University of Hawaii, 701 Ilalo Street, Honolulu, Hawaii 96813, USA; jturkson@cc.hawaii.edu (J.T.)

[3] xInstituto de Química Rosario (CONICET), Facultad de CienciasBioquímicas y Farmacéuticas, Universidad Nacional de Rosario, Suipacha 531, Rosario 2000, Argentina; sarotti@iquir-conicet.gov.ar (A. M. S.)

* Correspondence: scao@hawaii.edu; Tel.: +1-808-981-8017

Reference 35. Frisch, M. J.; Trucks, G. W.; Schlegel, H. B.; Scuseria, G. E.; Robb, M. A.; Cheeseman, J. R.; Scalmani, G.; Barone, V.; Mennucci, B.; Petersson, G. A.; Nakatsuji, H.; Caricato, M.; Li, X.; Hratchian, H. P.; Izmaylov, A. F.; Bloino, J.; Zheng, G.; Sonnenberg, J. L.; Hada, M.; Ehara, M.; Toyota, K.; Fukuda, R.; Hasegawa, J.; Ishida, M.; Nakajima, T.; Honda, Y.; Kitao, O.; Nakai, H.; Vreven, T.; Montgomery, J. A., Jr., Peralta, J. E.; Ogliaro, F.; Bearpark, M.; Heyd, J. J.; Brothers, E.; Kudin, K. N.; Staroverov, V. N.; Kobayashi, R.; Normand, J.; Raghavachari, K.; Rendell, A.; Burant, J. C.; Iyengar, S. S.; Tomasi, J.; Cossi, M.; Rega, N.; Millam, J. M.; Klene, M.; Knox, J. E.; Cross, J. B.; Bakken, V.; Adamo, C.; Jaramillo, J.; Gomperts, R.; Stratmann, R. E.; Yazyev, O.; Austin, A. J.; Cammi, R.; Pomelli, C.; Ochterski, J. W.; Martin, R. L.; Morokuma, K.; Zakrzewski, V. G.; Voth, G. A.; Salvador, P.; Dannenberg, J. J.; Dapprich, S.; Daniels, A. D.; Farkas, O.; Foresman, J. B.; Ortiz, J. V.; Cioslowski, J.; Fox, D. J. Gaussian 09, Gaussian, Inc.: Wallingford, CT, 2009.

Content

**Figure S1**	^{1}H NMR spectrum (400 MHz, methanol-d_4) of compound **1**................................	**S4**
**Figure S2**	^{1}H-^{1}H COSY of compound **1** in methanol-d_4..	**S5**
**Figure S3**	HSQC spectrum of compound **1** in methanol-d_4..	**S6**
**Figure S4**	HMBC spectrum of compound **1** in methanol-d_4...	**S7**
**Figure S5**	NOESY spectrum of compound **1** in methanol-d_4...	**S8**
**Figure S6**	^{1}H NMR spectrum (400 MHz, $CDCl_3$) of compound **1**..	**S9**
**Figure S7**	HSQC spectrum of compound **1** in $CDCl_3$...	**S10**
**Figure S9**	IR spectrum of compound **1**..	**S11**
**Figure S10**	HRESIMS of compound **1**..	**S12**
	Information of the ECD calculation..	**S13**

^{1}H spectrum (400 MHz, Methanol-*d*4) of compound **1**

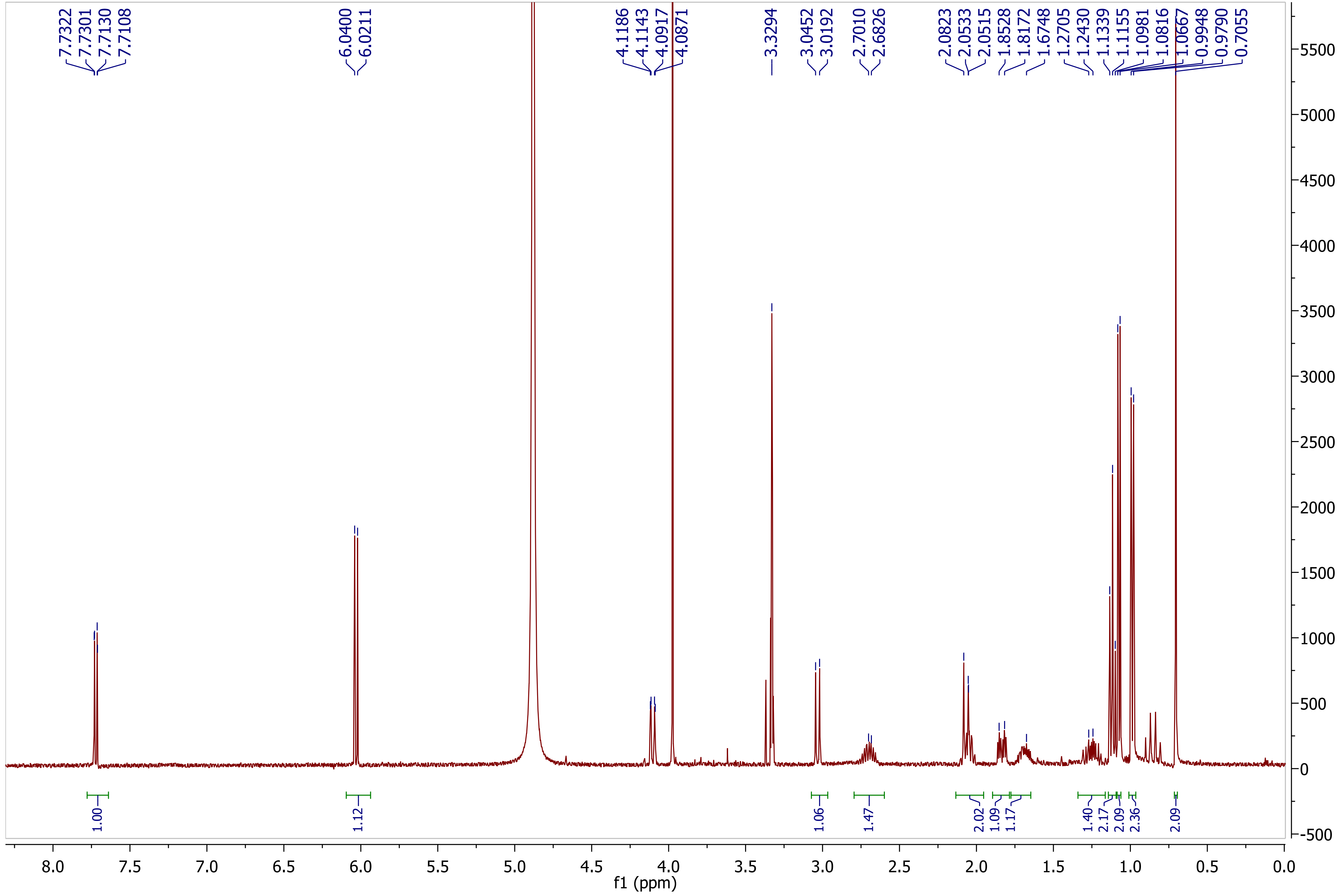

^{1}H-^{1}H COSY spetrum of compound **1** in methanol-d_4

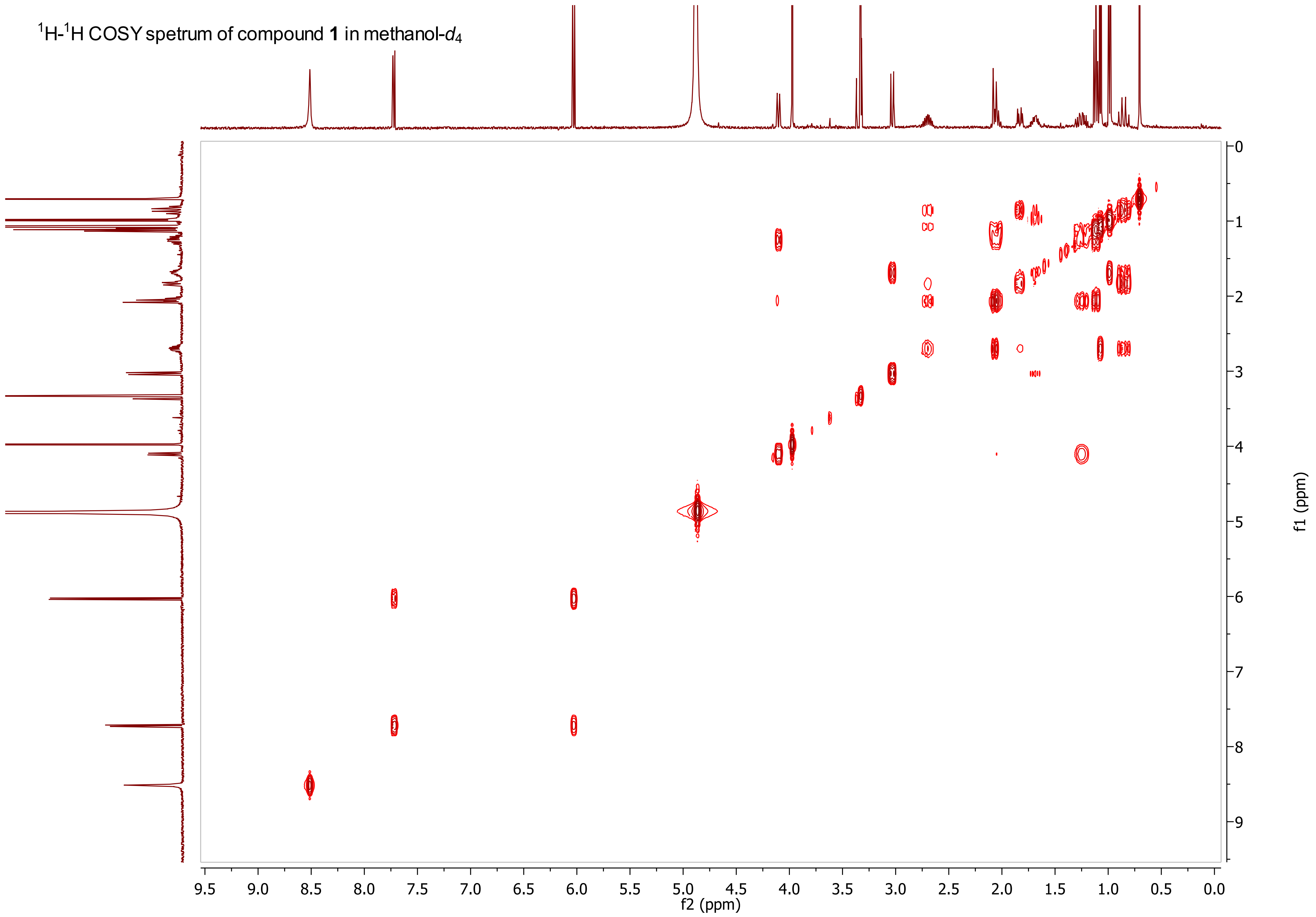

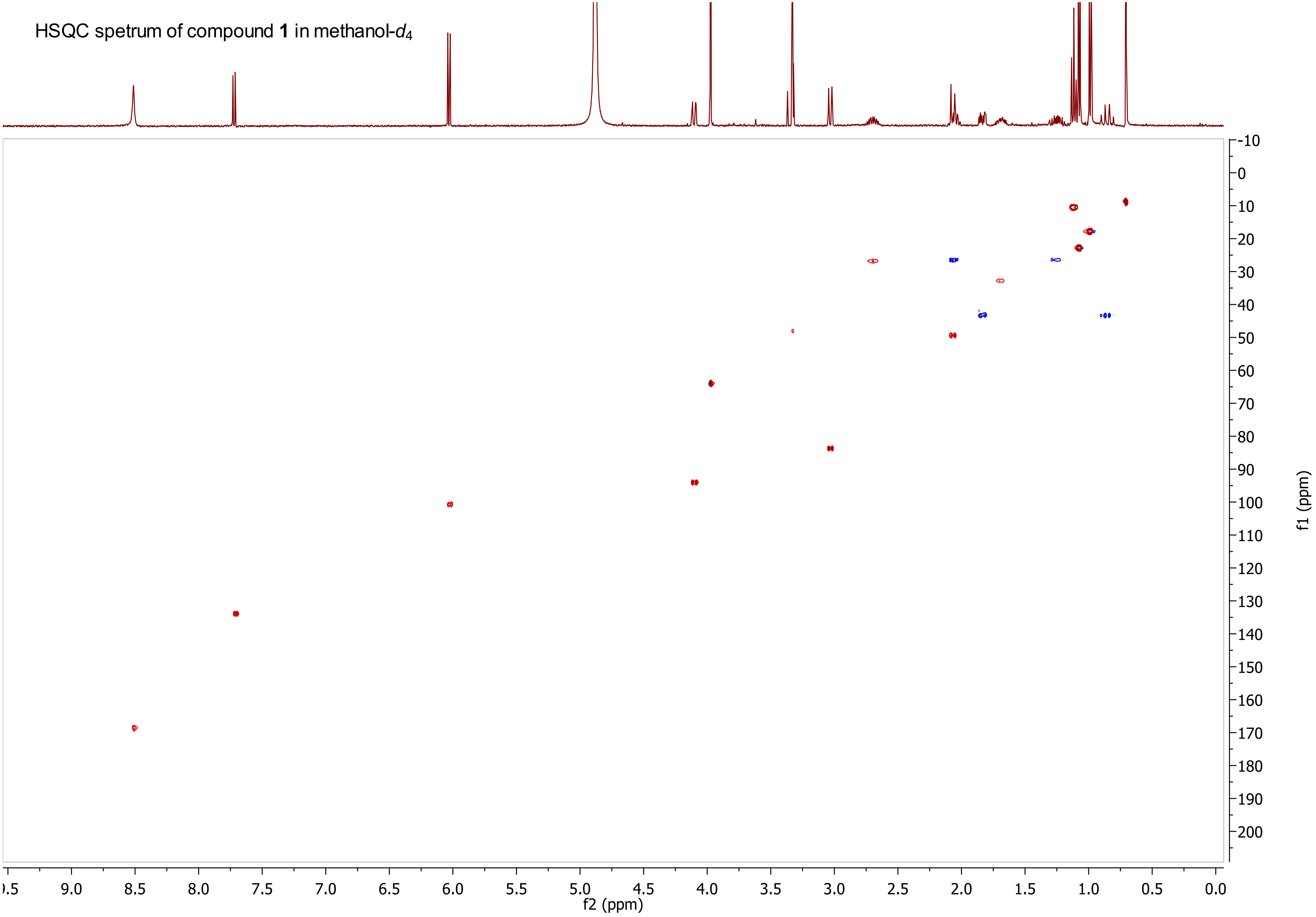

HSQC spetrum of compound **1** in methanol-d_4

HMBC spetrum of compound **1** in methanol-d_4

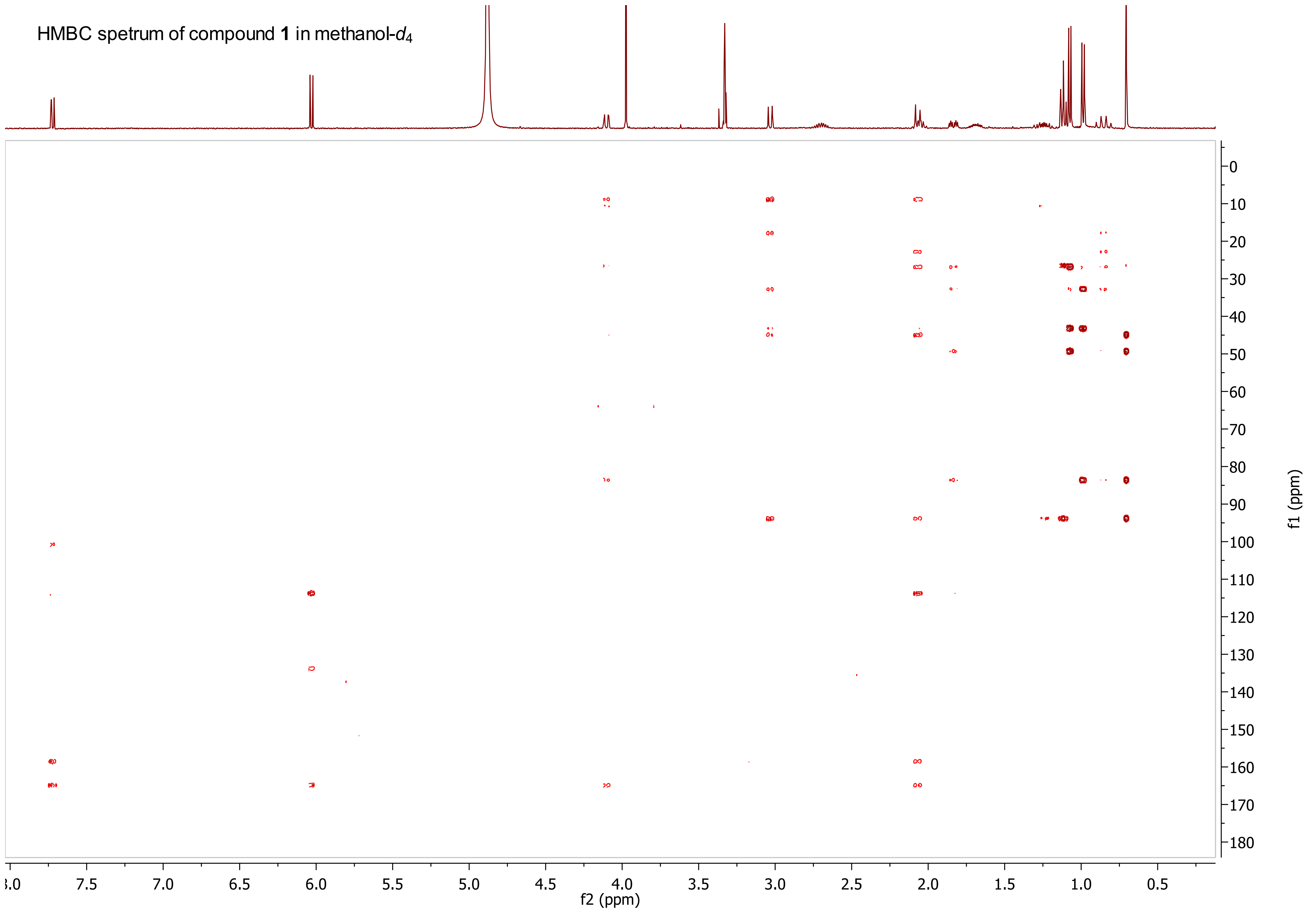

NOESY spetrum of compound **1** in methanol-d_4

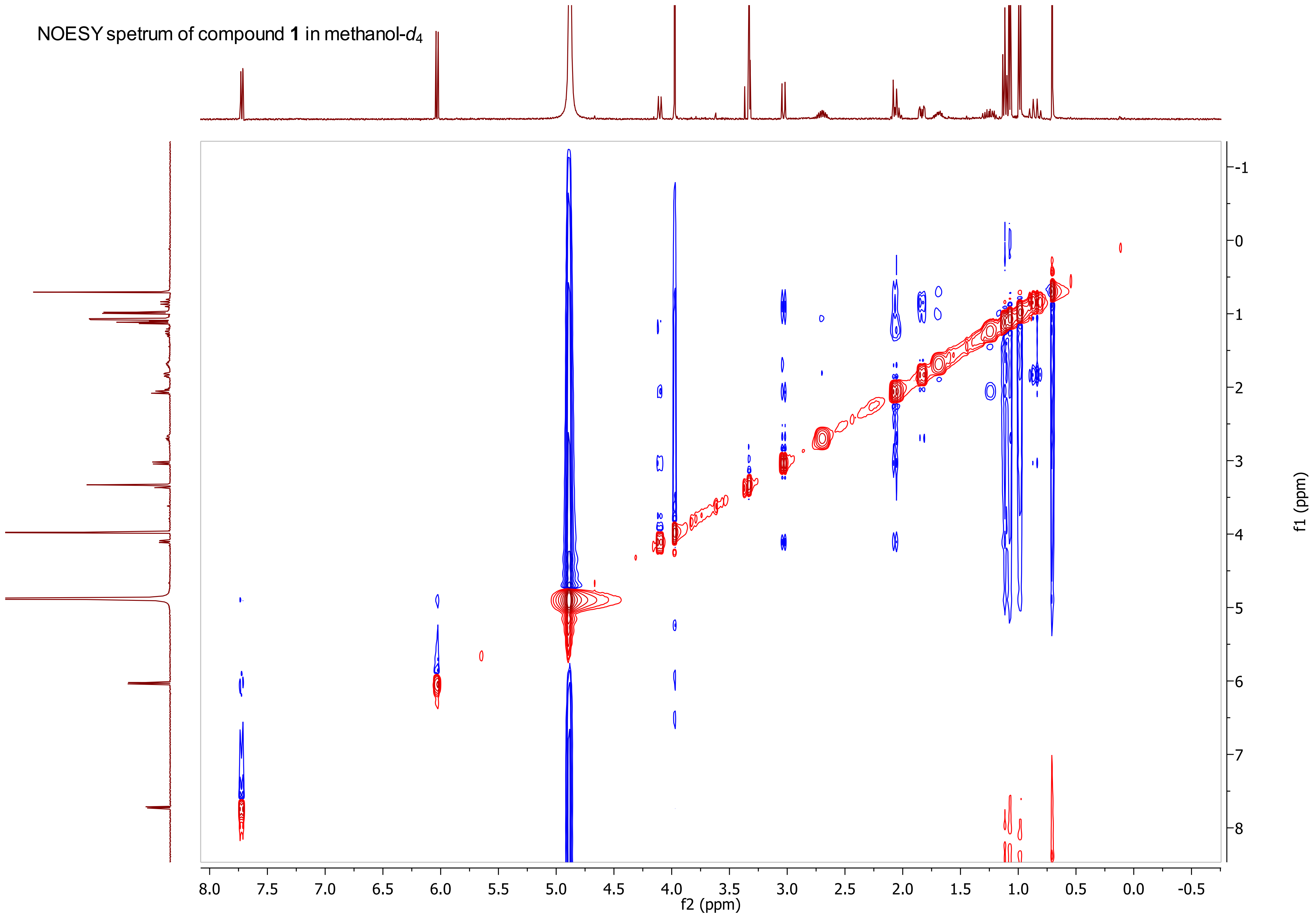

^{1}H spectrum (400 MHz, CDCl3) of compound **1**

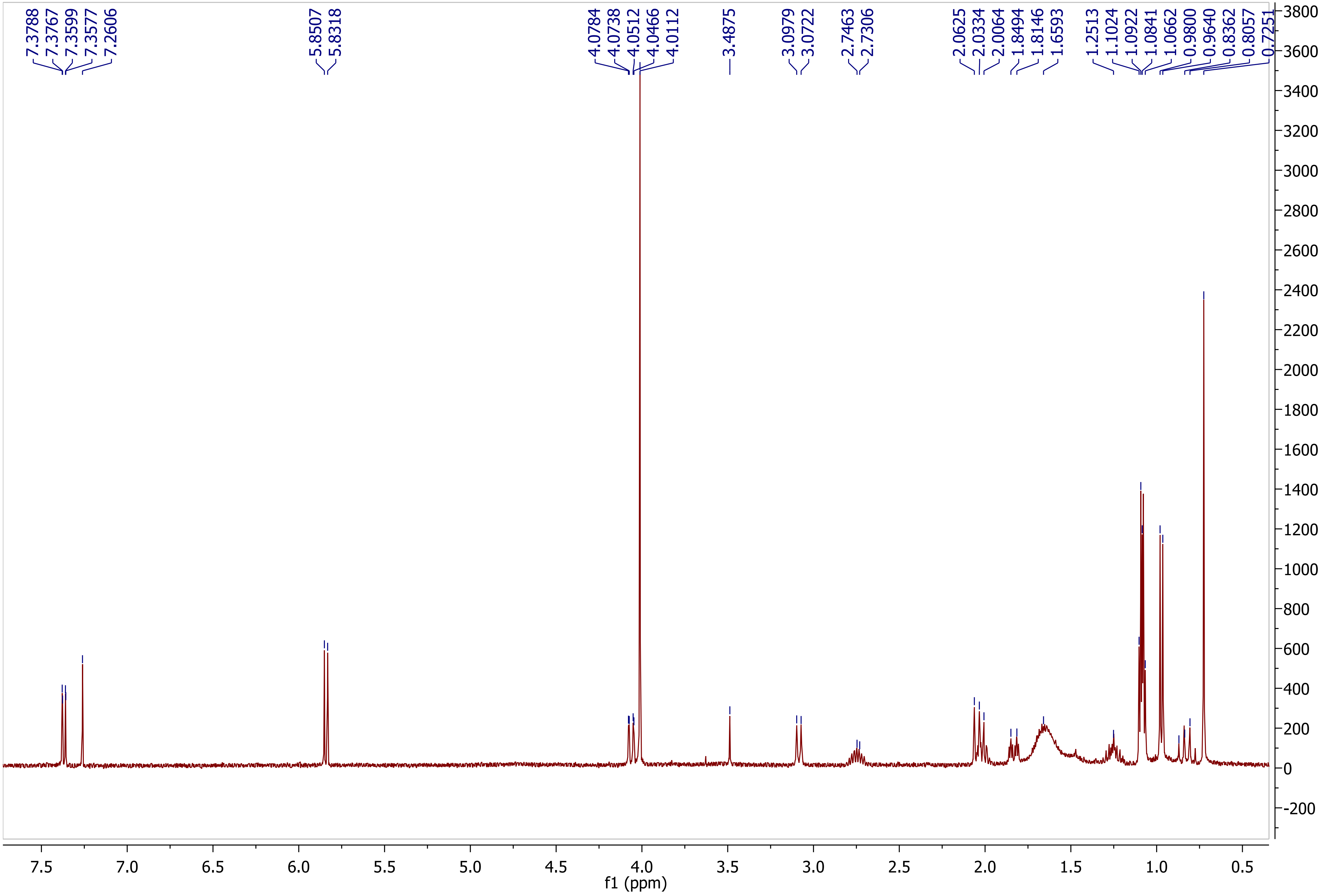

HSQC spetrum of compound **1** in $CDCl_3$

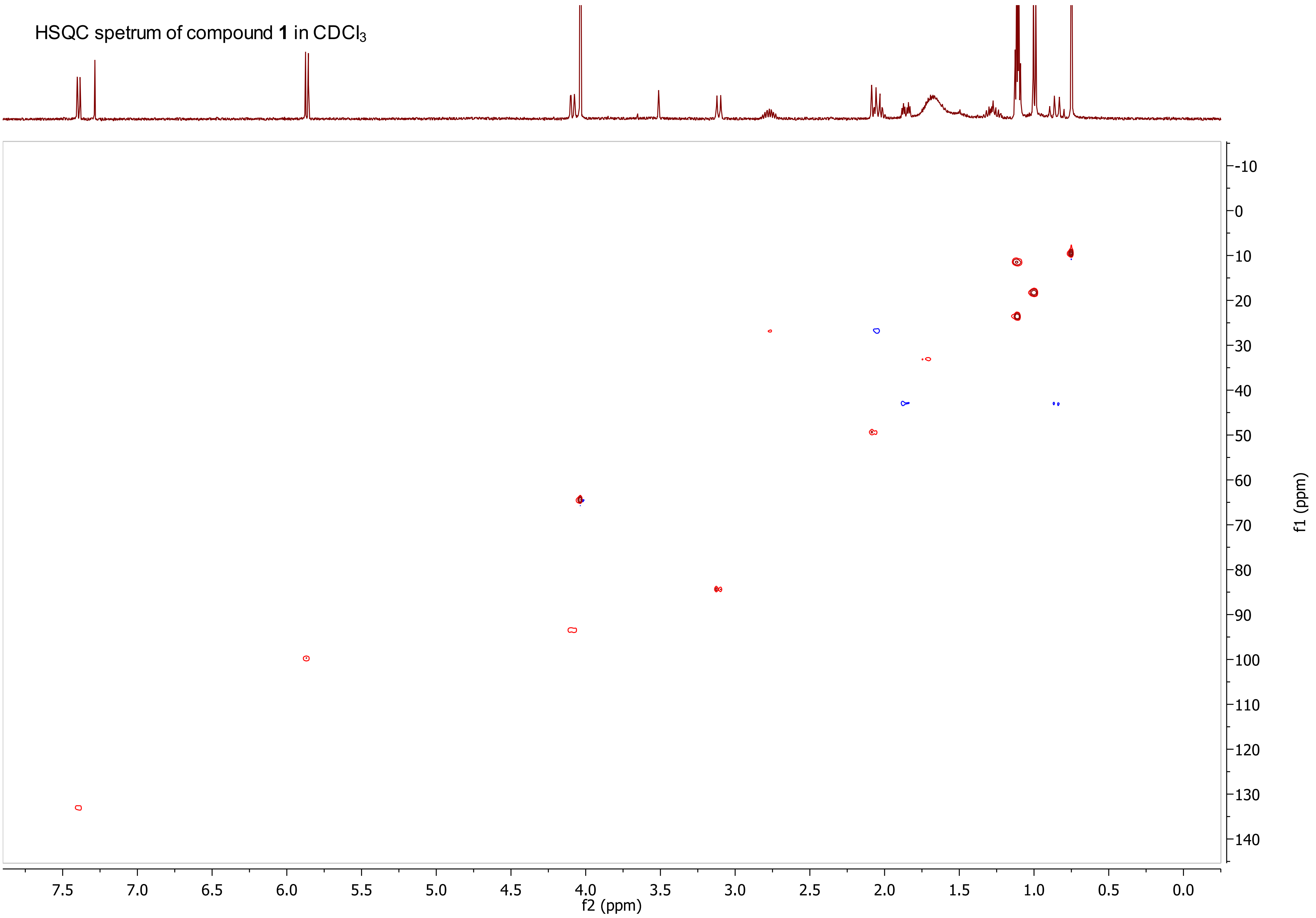

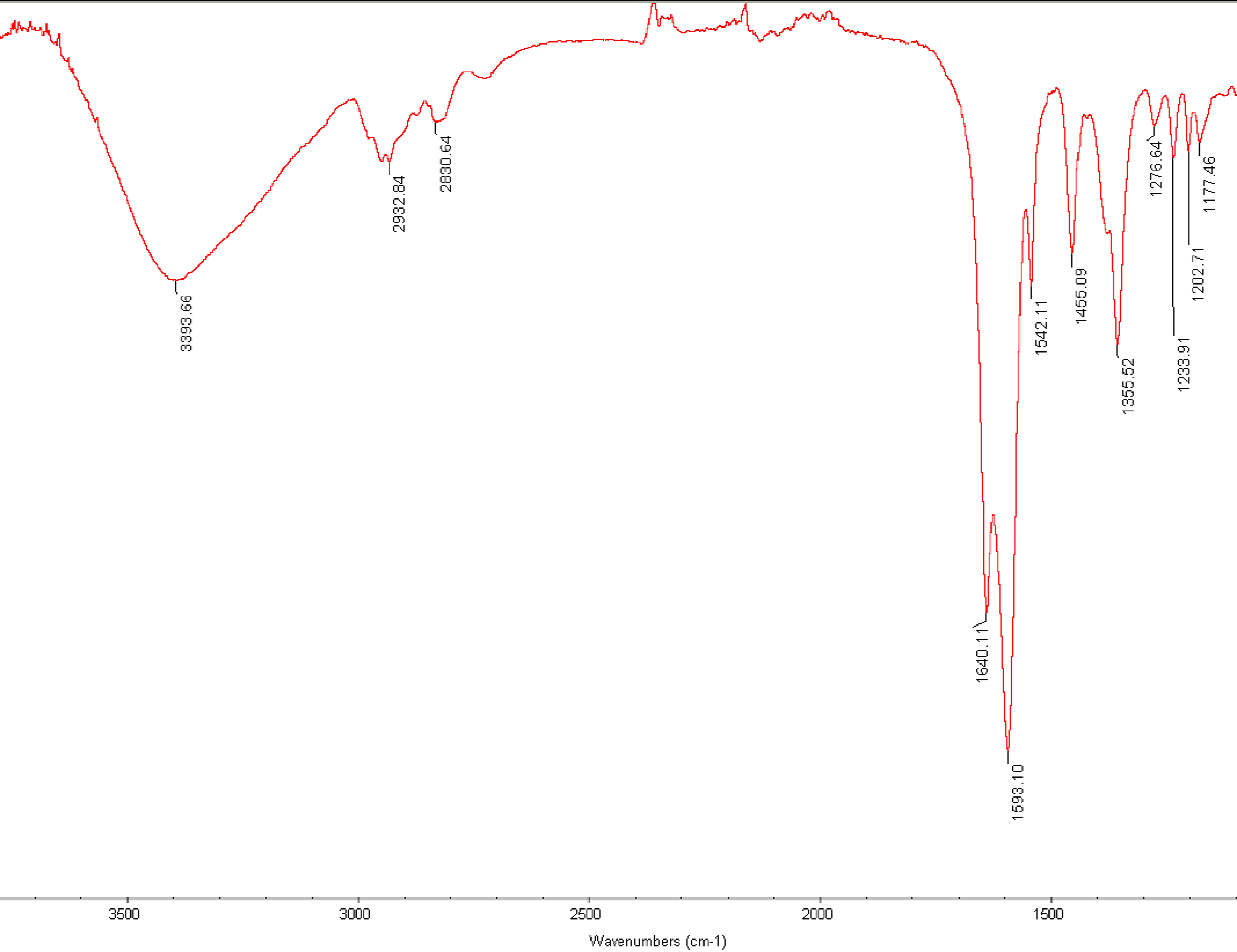
3393.66
2932.84
2830.64
1640.11
1593.10
1542.11
1455.09
1355.52
1276.64
1233.91
1202.71
1177.46
3500
3000
2500
2000
1500
Wavenumbers (cm-1)

HRESIMS of compound **1**

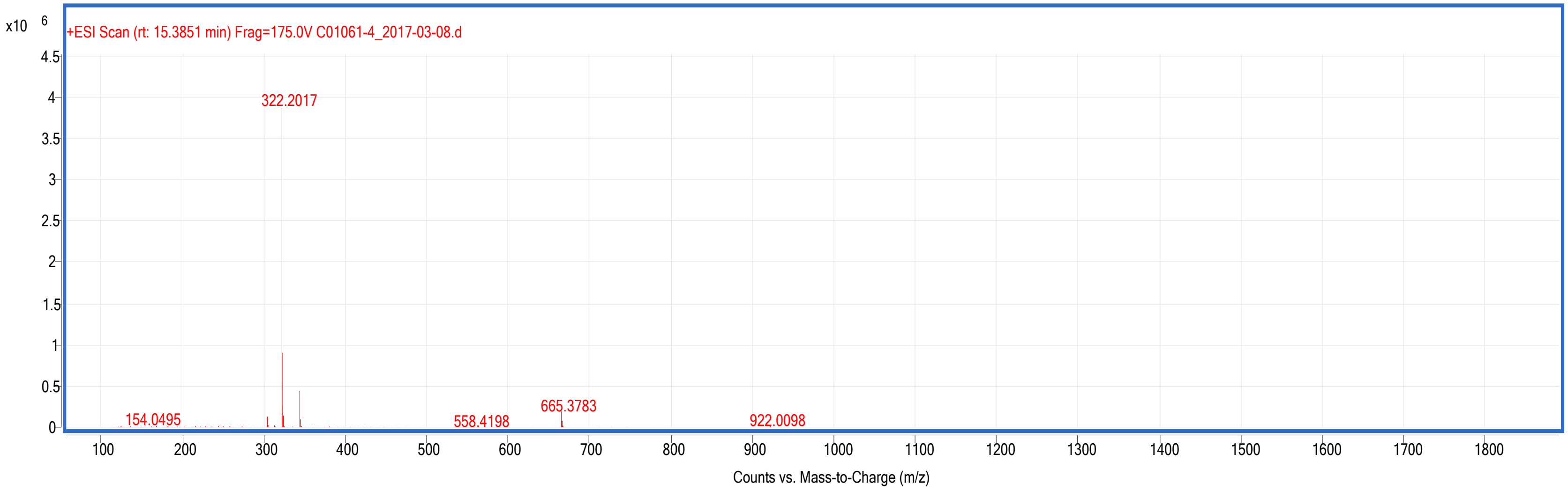

Cartesian Coordinates of all conformers found for compounds **1** and **3** at the B3LYP/6-31G* level of theory.

Compound 1_c01
B3LYP/6-31G* Geometry
C -2.268006 2.180728 0.613182
C -1.030172 0.129857 0.026084
N -3.431065 0.278504 -0.147375
C -2.258718 -0.453285 -0.495627
C -3.440713 1.548659 0.345811
C -1.054158 1.468712 0.374032
O 0.073244 2.208286 0.471032
C 1.209955 1.683683 -0.271433
C 1.521931 0.270060 0.292909
C 0.283759 -0.632492 -0.109469
C 0.431229 -2.011042 0.618254
C 1.666488 -2.691435 -0.005020
C 2.960111 -1.860964 0.052929
C 2.758792 -0.386654 -0.374432
O -2.362027 -1.470448 -1.176611
C 2.267346 2.786173 -0.221297
C 1.790860 4.084451 -0.884732
C 1.735060 0.401980 1.820929
H 0.389032 -0.858344 -1.182894
C 4.067775 -2.540397 -0.768951
C -0.746436 -2.994179 0.611408
O 3.923773 0.374505 -0.034619
O -4.646787 -0.163914 -0.661003
C -5.113190 -1.328576 0.040277
H -2.238480 3.207365 0.954636
H -4.423028 1.988945 0.462804
H 0.878692 1.567878 -1.316160
H 0.639168 -1.806792 1.674470
H 1.849471 -3.654619 0.489960
H 1.429967 -2.930189 -1.053621
H 3.298512 -1.827118 1.096778
H 2.605398 -0.353613 -1.468372
H 2.541330 2.982695 0.821139
H 3.169310 2.415145 -0.711475
H 2.577836 4.845900 -0.847026
H 1.536991 3.922016 -1.940065
H 0.904400 4.487062 -0.386003
H 0.809352 0.685358 2.328144
H 2.485351 1.167831 2.029811
H 2.095980 -0.520730 2.276549
H 4.202177 -3.580101 -0.449820
H 3.818794 -2.552063 -1.838044
H 5.042554 -2.049560 -0.651550
H -0.452093 -3.892129 1.171060
H -1.038549 -3.288952 -0.397502
H -1.629118 -2.576550 1.103540
H 4.677162 -0.022373 -0.496767
H -5.239093 -1.114726 1.108590
H -4.427936 -2.164737 -0.113234
H -6.082829 -1.546223 -0.412823
SCF Energy = -1057.75305409
Number of imaginary frequencies = 0

Compound 1_c02
B3LYP/6-31G* Geometry
C -2.264259 2.188093 0.607501
C -1.032013 0.132475 0.024591
N -3.433001 0.286719 -0.146643
C -2.262912 -0.448976 -0.494262
C -3.438844 1.558314 0.342450
C -1.052488 1.472121 0.370418
O 0.077367 2.209512 0.469003
C 1.213920 1.680008 -0.270446
C 1.518404 0.265851 0.291504
C 0.280336 -0.633716 -0.110668
C 0.427524 -2.013569 0.615607
C 1.659875 -2.695617 -0.012412
C 2.957693 -1.871011 0.035905
C 2.765774 -0.391259 -0.372409
O -2.369246 -1.467755 -1.171848
C 2.278413 2.775822 -0.215771
C 1.822764 4.068374 -0.904480
C 1.722831 0.396210 1.821752
H 0.387589 -0.858481 -1.184247
C 4.065735 -2.529460 -0.798206
C -0.752273 -2.994211 0.611548
O 3.981677 0.326836 -0.131698
O -4.650454 -0.154322 -0.657014
C -5.118412 -1.316481 0.047651
H -2.231936 3.215813 0.945405
H -4.419895 2.001743 0.458166
H 0.886959 1.566245 -1.316408
H 0.637586 -1.811459 1.672273
H 1.841571 -3.660880 0.478730
H 1.419794 -2.930269 -1.060770
H 3.298850 -1.858067 1.084072
H 2.636742 -0.336241 -1.461771
H 2.528966 2.988436 0.830422
H 3.188403 2.393511 -0.682521
H 2.610534 4.828154 -0.855023
H 1.596584 3.894412 -1.964016
H 0.924640 4.478170 -0.432961
H 0.790466 0.653455 2.329979
H 2.440017 1.192614 2.045103
H 2.102020 -0.519529 2.279758
H 4.278088 -3.542041 -0.435751
H 3.763746 -2.609893 -1.850356
H 4.989272 -1.945228 -0.757398
H -0.458078 -3.892891 1.169961
H -1.046872 -3.288011 -0.396844
H -1.633213 -2.575161 1.105674
H 4.198808 0.223784 0.808823
H -5.241943 -1.099954 1.115699
H -4.435539 -2.154660 -0.105335
H -6.089324 -1.532588 -0.403351
SCF Energy = -1057.75433022
Number of imaginary frequencies = 0

Compound 1_c03
B3LYP/6-31G* Geometry
C -2.272457 2.186466 -0.459728
C -1.043241 0.051877 -0.312684
N -3.360378 0.233180 0.284171
C -2.196570 -0.596768 0.278310
C -3.399838 1.554776 -0.033555
C -1.080854 1.412581 -0.558911
O 0.026101 2.085848 -0.936300
C 1.355263 1.676339 -0.458665
C 1.379525 0.270903 0.224065
C 0.316521 -0.594623 -0.553512
C 0.423673 -2.126791 -0.358666
C 1.881561 -2.591866 -0.511280
C 2.894804 -1.828901 0.348278
C 2.808326 -0.331524 0.014284
O -2.286018 -1.740435 0.727060
C 1.896200 2.854921 0.356977
C 2.007884 4.147818 -0.458585
C 1.040525 0.336133 1.728354
H 0.542370 -0.424941 -1.621339
C 4.316342 -2.370804 0.141173
C -0.437239 -2.883204 -1.384336
O 3.823065 0.419219 0.692518
O -4.502335 -0.264592 0.901295
C -5.162278 -1.237379 0.071943
H -2.263329 3.242963 -0.693838
H -4.362217 2.031866 0.103983
H 1.937811 1.598807 -1.384777
H 0.065780 -2.390466 0.641248
H 1.938024 -3.664046 -0.281707
H 2.183293 -2.493470 -1.566756
H 2.627884 -1.964906 1.408819
H 3.066326 -0.217614 -1.047664
H 1.243575 3.018516 1.223472
H 2.880361 2.565546 0.736259
H 2.403357 4.961487 0.159478
H 2.686468 4.018843 -1.311027
H 1.034122 4.458269 -0.848705
H 0.117110 0.890673 1.910646
H 1.830603 0.835661 2.298304

H 0.899169 -0.665208 2.145895
H 4.366649 -3.431988 0.411038
H 4.617820 -2.278707 -0.910105
H 5.050171 -1.826442 0.741984
H -0.292084 -3.964786 -1.273540
H -0.146853 -2.614729 -2.409394
H -1.499522 -2.679176 -1.249939
H 3.767568 0.199002 1.636032
H -4.519917 -2.107178 -0.077049
H -5.454791 -0.794432 -0.887875
H -6.052505 -1.515844 0.640010
SCF Energy = -1057.75901641
Number of imaginary frequencies = 0

Compound 1_c04
B3LYP/6-31G* Geometry
C -2.273468 2.167642 -0.486314
C -1.037098 0.039036 -0.318703
N -3.350817 0.219165 0.286478
C -2.186418 -0.610278 0.278196
C -3.395996 1.537352 -0.044690
C -1.081109 1.395586 -0.583959
O 0.024957 2.057909 -0.986225
C 1.344679 1.687144 -0.450930
C 1.379720 0.273502 0.219063
C 0.325252 -0.599605 -0.559468
C 0.442671 -2.130637 -0.355617
C 1.905002 -2.591931 -0.489916
C 2.900221 -1.805472 0.369825
C 2.797159 -0.319286 -0.009175
O -2.273527 -1.753803 0.727793
C 1.811502 2.871712 0.401775
C 1.931090 4.173637 -0.398186
C 1.056267 0.323523 1.728199
H 0.548850 -0.430580 -1.627927
C 4.328660 -2.341843 0.193360
C -0.408168 -2.897749 -1.381320
O 3.734902 0.490630 0.708716
O -4.489356 -0.276758 0.911297
C -5.152900 -1.253066 0.089139
H -2.269392 3.221447 -0.732429
H -4.358631 2.013223 0.095330
H 1.966447 1.639777 -1.353033
H 0.078798 -2.389172 0.643155
H 1.963189 -3.659802 -0.241043
H 2.214387 -2.510983 -1.544989
H 2.629548 -1.913437 1.428503
H 3.014694 -0.246656 -1.090869
H 1.109395 3.010613 1.232639
H 2.779572 2.606555 0.834921
H 2.266328 4.994078 0.246146
H 2.660233 4.072233 -1.212094
H 0.972389 4.458782 -0.841614
H 0.163804 0.921911 1.927589
H 1.889418 0.751750 2.289861
H 0.862312 -0.678585 2.121218
H 4.367451 -3.414260 0.414557
H 4.681932 -2.207545 -0.838430
H 5.039517 -1.851616 0.869415
H -0.260479 -3.978151 -1.262137
H -0.113164 -2.635206 -2.406644
H -1.472025 -2.696164 -1.254226
H 4.624325 0.195088 0.464320
H -4.510086 -2.122283 -0.061154
H -5.452071 -0.813506 -0.870254
H -6.039232 -1.531100 0.663514
SCF Energy = -1057.75884366
Number of imaginary frequencies = 0

Compound 1_c05
B3LYP/6-31G* Geometry
C -2.371199 1.956793 0.845393
C -1.028807 -0.001688 0.173901
N -3.440079 -0.027348 0.172238
C -2.246183 -0.696748 -0.219072
C -3.509600 1.231591 0.684261
C -1.130303 1.337433 0.508159
O -0.059442 2.164373 0.506732
C 1.060972 1.705561 -0.300848
C 1.512301 0.333456 0.266726
C 0.327147 -0.668510 -0.038526
C 0.630791 -2.017364 0.699836
C 1.875244 -2.610011 0.007798
C 3.102937 -1.685121 -0.039683
C 2.765918 -0.235962 -0.463006
O -2.325652 -1.739553 -0.864507
C 2.037775 2.880462 -0.346570
C 1.424858 4.124333 -1.002699
C 1.799213 0.512499 1.779115
H 0.381059 -0.907554 -1.113262
C 4.208083 -2.272928 -0.928550
C -0.458144 -3.095125 0.785134
O 3.930140 0.585076 -0.315373
O -4.644050 -0.700786 0.016551
C -5.091439 -0.672475 -1.352733
H -2.391068 2.982536 1.190197
H -4.507545 1.581231 0.918421
H 0.672006 1.540168 -1.318497
H 0.886222 -1.775628 1.738123
H 2.162951 -3.546884 0.503052
H 1.591608 -2.887000 -1.019209
H 3.504943 -1.621075 0.984688
H 2.564547 -0.219206 -1.542755
H 2.361928 3.126975 0.671491
H 2.930932 2.562461 -0.888059
H 2.154172 4.941311 -1.033251
H 1.116675 3.917004 -2.035339
H 0.545245 4.473106 -0.453963
H 0.881385 0.692257 2.343884
H 2.452415 1.375092 1.946016
H 2.288702 -0.357011 2.222773
H 4.519042 -3.258864 -0.563951
H 3.852287 -2.397138 -1.959420
H 5.084167 -1.618808 -0.952170
H -0.052443 -3.950685 1.341262
H -0.783100 -3.438371 -0.198097
H -1.344413 -2.745766 1.320505
H 4.212827 0.525020 0.611349
H -4.381253 -1.196742 -1.993769
H -5.238371 0.361032 -1.690558
H -6.049742 -1.196091 -1.334180
SCF Energy = -1057.75396001
Number of imaginary frequencies = 0

Compound 1_c06
B3LYP/6-31G* Geometry
C -2.374587 1.948186 0.851126
C -1.026989 -0.004456 0.173468
N -3.437856 -0.036919 0.172353
C -2.242213 -0.701384 -0.221840
C -3.510879 1.220192 0.688921
C -1.132114 1.333582 0.510378
O -0.064603 2.163174 0.507061
C 1.056468 1.709095 -0.301808
C 1.514692 0.338173 0.268163
C 0.330522 -0.666901 -0.039702
C 0.634015 -2.015284 0.698746
C 1.882645 -2.604672 0.012181
C 3.105755 -1.673462 -0.022500
C 2.759726 -0.230202 -0.462096
O -2.319274 -1.741827 -0.871928
C 2.025690 2.889819 -0.349953
C 1.397805 4.136005 -0.987082
C 1.807636 0.519198 1.778132
H 0.383657 -0.905560 -1.114472
C 4.214781 -2.280535 -0.897644
C -0.452124 -3.096275 0.778489
O 3.875125 0.631122 -0.206387
O -4.640122 -0.712989 0.013970
C -5.087840 -0.679181 -1.354846
H -2.396892 2.972542 1.199904
H -4.509655 1.565708 0.925644
H 0.665100 1.541266 -1.318479
H 0.885628 -1.772847 1.737316

H 2.171427 -3.539839 0.510289
H 1.604341 -2.884189 -1.015950
H 3.501092 -1.589554 0.998266
H 2.539766 -0.234026 -1.545341
H 2.368360 3.124970 0.663727
H 2.910934 2.580130 -0.908515
H 2.124913 4.954733 -1.026484
H 1.069235 3.935035 -2.014941
H 0.528408 4.480264 -0.419648
H 0.893699 0.729330 2.339300
H 2.498244 1.351976 1.928637
H 2.275609 -0.360190 2.222201
H 4.455463 -3.296402 -0.564666
H 3.902563 -2.341792 -1.948268
H 5.150117 -1.707513 -0.853452
H -0.046069 -3.951617 1.334842
H -0.773455 -3.438988 -0.206199
H -1.340844 -2.750124 1.311749
H 4.635134 0.276112 -0.691254
H -4.376243 -1.198254 -1.998583
H -5.237731 0.355562 -1.687687
H -6.044740 -1.205524 -1.338921
SCF Energy = -1057.75269061
Number of imaginary frequencies = 0

Compound 1_c07
B3LYP/6-31G* Geometry
C -2.375812 2.013207 -0.717076
C -1.027947 -0.041095 -0.488500
N -3.385474 -0.006376 -0.048068
C -2.184884 -0.780513 -0.027757
C -3.490750 1.305700 -0.387102
C -1.133274 1.317472 -0.724045
O -0.041914 2.057802 -1.013665
C 1.257178 1.739243 -0.402328
C 1.325200 0.310244 0.230547
C 0.382670 -0.596167 -0.648441
C 0.574718 -2.124865 -0.481475
C 2.067972 -2.496548 -0.513981
C 2.947699 -1.683658 0.441307
C 2.787714 -0.194750 0.095197
O -2.242567 -1.945274 0.370135
C 1.602482 2.921627 0.509363
C 1.701807 4.251311 -0.246553
C 0.889781 0.296064 1.712135
H 0.672079 -0.379608 -1.692199
C 4.414084 -2.134172 0.361703
C -0.146584 -2.904063 -1.594030
O 3.621370 0.642917 0.904055
O -4.579863 -0.665439 0.212117
C -4.749534 -0.905721 1.621686
H -2.418029 3.067283 -0.958393
H -4.495486 1.709118 -0.360099
H 1.938952 1.749935 -1.261419
H 0.151178 -2.437481 0.477753
H 2.170372 -3.567103 -0.292503
H 2.450313 -2.363143 -1.539213
H 2.604340 -1.838941 1.472696
H 3.081121 -0.076363 -0.964207
H 0.841384 2.999730 1.295407
H 2.553283 2.696010 0.999163
H 1.945665 5.069042 0.440890
H 2.488954 4.214835 -1.010270
H 0.760616 4.496597 -0.747490
H -0.048132 0.837326 1.859603
H 1.653039 0.753065 2.345988
H 0.726441 -0.727271 2.062461
H 4.497303 -3.209710 0.553419
H 4.837100 -1.945934 -0.634833
H 5.041864 -1.628673 1.105452
H 0.055875 -3.977511 -1.494406
H 0.213441 -2.590948 -2.583769
H -1.227203 -2.768659 -1.548278
H 4.541711 0.402223 0.721816
H -4.765333 0.040616 2.176647
H -3.961286 -1.563680 1.990781
H -5.722572 -1.396055 1.696444
SCF Energy = -1057.75857559
Number of imaginary frequencies = 0

Compound 1_c08
B3LYP/6-31G* Geometry
C -2.374600 2.033203 -0.692498
C -1.034143 -0.028476 -0.483996
N -3.394219 0.010558 -0.050486
C -2.195211 -0.765508 -0.028287
C -3.493729 1.326074 -0.377273
C -1.133493 1.334062 -0.700854
O -0.042848 2.083198 -0.964970
C 1.270791 1.728501 -0.409472
C 1.325557 0.306828 0.235659
C 0.374102 -0.592444 -0.643502
C 0.556134 -2.122555 -0.485465
C 2.046483 -2.497961 -0.537449
C 2.945425 -1.708222 0.419111
C 2.798506 -0.208522 0.119930
O -2.256334 -1.930275 0.369234
C 1.691040 2.909399 0.471319
C 1.784070 4.230957 -0.299411
C 0.872370 0.306524 1.711113
H 0.666209 -0.375564 -1.686398
C 4.407551 -2.163631 0.308684
C -0.176913 -2.892251 -1.597356
O 3.714406 0.576029 0.893977
O -4.591275 -0.647653 0.199025
C -4.772188 -0.892078 1.606476
H -2.411590 3.090070 -0.922143
H -4.497388 1.732322 -0.352913
H 1.917852 1.707825 -1.295099
H 0.139531 -2.439149 0.475825
H 2.148830 -3.572432 -0.336231
H 2.419899 -2.346734 -1.563170
H 2.606291 -1.892011 1.451507
H 3.128123 -0.047423 -0.915661
H 0.974663 3.012025 1.296059
H 2.662344 2.662039 0.908673
H 2.089678 5.045274 0.366942
H 2.525472 4.166590 -1.105486
H 0.823009 4.499675 -0.747596
H -0.091021 0.806035 1.837553
H 1.588968 0.828272 2.353584
H 0.755606 -0.713948 2.088421
H 4.497851 -3.228928 0.550320
H 4.782823 -2.019643 -0.712511
H 5.060773 -1.600323 0.980906
H 0.022978 -3.966786 -1.505238
H 0.177998 -2.574824 -2.587508
H -1.256575 -2.753727 -1.543804
H 3.610529 0.314571 1.822685
H -4.790816 0.052565 2.164317
H -3.987994 -1.552610 1.979556
H -5.746621 -1.380866 1.672299
SCF Energy = -1057.75860721
Number of imaginary frequencies = 0

Compound 1_c09
B3LYP/6-31G* Geometry
C 2.290101 -2.271041 0.648684
C 1.089693 -0.208142 0.026494
N 3.483635 -0.415612 -0.178067
C 2.323093 0.334957 -0.525543
C 3.472215 -1.672963 0.346986
C 1.088416 -1.538151 0.410314
O -0.052023 -2.252772 0.541704
C -1.187851 -1.715939 -0.197116
C -1.447870 -0.287326 0.356057
C -0.209073 0.581616 -0.103177
C -0.312995 1.978377 0.597932
C -1.554203 2.666557 -0.003852
C -2.859711 1.866362 0.135566
C -2.703675 0.378741 -0.260503
O 2.439133 1.334536 -1.230187
C -2.272643 -2.802327 -0.118189
C -3.074085 -2.967096 -1.414410

C -1.585466 -0.392833 1.896561
H -0.335402 0.784272 -1.178903
C -3.991308 2.544728 -0.653895
C   0.880797 2.940025 0.536004
O -3.856575 -0.358003 0.163086
O   4.701068 -0.013394 -0.719922
C   5.204403 1.156222 -0.053270
H   2.242777 -3.287958 1.016183
H   4.446073 -2.132755 0.459590
H -0.858994 -1.626297 -1.244292
H -0.496321 1.800940 1.664034
H -1.697179 3.649779 0.464116
H -1.353749 2.863881 -1.068412
H -3.149491 1.869212 1.194554
H -2.609868 0.315775 -1.359659
H -1.746645 -3.740242 0.095145
H -2.953201 -2.616609 0.715231
H -3.640417 -2.062439 -1.650464
H -2.417932 -3.198834 -2.262683
H -3.791453 -3.789712 -1.316511
H -0.616624 -0.566670 2.371467
H -2.244426 -1.221526 2.164858
H -2.016506 0.506257 2.338948
H -4.094048 3.594151 -0.355527
H -3.789756 2.525283 -1.732725
H -4.968350 2.074615 -0.482139
H   0.616737 3.856784 1.079982
H   1.151265 3.204195 -0.487437
H   1.769095 2.519348 1.014931
H -4.632399 0.044726 -0.254705
H   5.344074 0.963764 1.017421
H   4.534835 2.003537 -0.214520
H   6.170600 1.342269 -0.527319
SCF Energy = -1057.75041524
Number of imaginary frequencies = 0

Compound 1_c10
B3LYP/6-31G* Geometry
C   2.289546 -2.274206 0.643109
C   1.091815 -0.208322 0.024384
N   3.486315 -0.417681 -0.176619
C   2.327108 0.335306 -0.523763
C   3.472719 -1.676466 0.344491
C   1.089125 -1.539346 0.405470
O -0.052585 -2.253814 0.535633
C -1.186632 -1.713565 -0.204135
C -1.444431 -0.287192 0.351428
C -0.207132 0.582400 -0.105566
C -0.314152 1.979904 0.594444
C -1.554925 2.666767 -0.010858
C -2.862713 1.869431 0.122699
C -2.712569 0.377963 -0.256127
O   2.445304 1.336988 -1.224568
C -2.271992 -2.799593 -0.133894
C -3.096103 -2.939728 -1.419166
C -1.574242 -0.396854 1.893280
H -0.335486 0.784439 -1.181307
C -4.000678 2.523777 -0.673454
C   0.879388 2.942032 0.534606
O -3.917686 -0.321769 0.075079
O   4.705066 -0.015285 -0.715076
C   5.209356 1.151475 -0.043983
H   2.240722 -3.292419 1.006796
H   4.445867 -2.137927 0.456375
H -0.856785 -1.621123 -1.250467
H -0.498602 1.804012 1.661102
H -1.697927 3.651942 0.452748
H -1.353559 2.859567 -1.075756
H -3.150796 1.891795 1.186688
H -2.646702 0.296837 -1.349315
H -1.742342 -3.741066 0.053522
H -2.939503 -2.635770 0.715719
H -3.685955 -2.042112 -1.618765
H -2.450588 -3.137598 -2.284029
H -3.794519 -3.779470 -1.330092
H -0.602606 -0.552340 2.367921
H -2.205764 -1.246597 2.170118
H -2.013583 0.496648 2.343435
H -4.178598 3.549389 -0.329669
H -3.750873 2.569904 -1.741209
H -4.930495 1.957191 -0.570314
H   0.613549 3.858955 1.077271
H   1.151231 3.205686 -0.488549
H   1.767261 2.522237 1.015151
H -4.095124 -0.163113 1.016031
H   6.176599 1.337112 -0.515969
H   5.346988 0.955391 1.026308
H   4.541663 2.000524 -0.203744
SCF Energy = -1057.75130038
Number of imaginary frequencies = 0

Compound 1_c11
B3LYP/6-31G* Geometry
C -2.222974 2.335265 0.350346
C -1.078722 0.188188 -0.068952
N -3.481022 0.408405 -0.149272
C -2.350057 -0.396535 -0.474401
C -3.426809 1.722896 0.204669
C -1.044615 1.558676 0.135488
O   0.105059 2.270607 0.100589
C   1.192039 1.607879 -0.598763
C   1.469375 0.268916 0.137198
C   0.205750 -0.635338 -0.170603
C   0.332941 -1.951093 0.671101
C   1.565692 -2.698377 0.120839
C   2.875091 -1.891923 0.126933
C   2.698099 -0.468936 -0.446908
O -2.520498 -1.470892 -1.044677
C   2.259708 2.684271 -0.837921
C   2.888217 3.386624 0.370618
C   1.652228 0.537800 1.651945
H   0.283026 -0.953982 -1.222706
C   3.984915 -2.663577 -0.605285
C -0.852535 -2.923373 0.728778
O   3.864028 0.319349 -0.179574
O -4.733497 -0.039745 -0.559858
C -5.198910 -1.113872 0.273990
H -2.143225 3.390297 0.578236
H -4.387800 2.208762 0.318211
H   0.803959 1.344680 -1.595507
H   0.532044 -1.663561 1.709551
H   1.722896 -3.621359 0.694700
H   1.337701 -3.018197 -0.908056
H   3.196059 -1.763798 1.168857
H   2.551431 -0.547582 -1.539778
H   3.046433 2.239503 -1.453471
H   1.759649 3.440629 -1.457898
H   3.434572 4.275515 0.033741
H   2.125739 3.716712 1.083431
H   3.600578 2.738237 0.885884
H   0.711847 0.848534 2.114410
H   2.385590 1.324861 1.819338
H   2.006089 -0.344155 2.187836
H   4.099785 -3.668509 -0.183626
H   3.752400 -2.776873 -1.672199
H   4.963794 -2.174356 -0.519939
H -0.563739 -3.785221 1.345313
H -1.145906 -3.281786 -0.259251
H -1.733273 -2.470175 1.190023
H   4.618400 -0.118341 -0.601287
H -5.264434 -0.793398 1.320832
H -4.547601 -1.984287 0.171322
H -6.196199 -1.340494 -0.109325
SCF Energy = -1057.74902544
Number of imaginary frequencies = 0

Compound 1_c12
B3LYP/6-31G* Geometry
C -2.220413 2.338565 0.338838
C -1.079031 0.188123 -0.071877
N -3.481474 0.410618 -0.149226
C -2.352045 -0.397296 -0.472089
C -3.425204 1.726794 0.197498
C -1.043259 1.559500 0.127372

O 0.108035 2.270693 0.091691
C 1.194615 1.602619 -0.603374
C 1.467009 0.266464 0.134934
C 0.205007 -0.637251 -0.172036
C 0.334793 -1.953054 0.669608
C 1.562821 -2.701720 0.110391
C 2.875895 -1.901369 0.107485
C 2.709096 -0.468452 -0.441714
O -2.524384 -1.474460 -1.035895
C 2.269261 2.671707 -0.845015
C 2.881052 3.394674 0.360290
C 1.639610 0.539360 1.651307
H 0.284472 -0.955773 -1.224119
C 3.985565 -2.652431 -0.641908
C -0.852047 -2.923075 0.736324
O 3.924392 0.267321 -0.252767
O -4.735229 -0.038510 -0.554367
C -5.199709 -1.108383 0.285633
H -2.139399 3.394804 0.560577
H -4.385530 2.214440 0.308961
H 0.808892 1.337301 -1.600203
H 0.540631 -1.665986 1.707411
H 1.720760 -3.627977 0.678595
H 1.329378 -3.015496 -0.918740
H 3.199002 -1.797369 1.156338
H 2.594353 -0.519802 -1.533234
H 3.064534 2.217878 -1.442433
H 1.780200 3.421202 -1.481446
H 3.426702 4.281431 0.018021
H 2.111363 3.728277 1.063529
H 3.596281 2.762298 0.893043
H 0.688990 0.810680 2.117061
H 2.329692 1.365519 1.827408
H 2.023558 -0.330279 2.189874
H 4.174458 -3.630146 -0.183542
H 3.699555 -2.824980 -1.687383
H 4.918876 -2.082044 -0.639524
H -0.561237 -3.783615 1.353598
H -1.150595 -3.283667 -0.249268
H -1.729889 -2.467178 1.200583
H 4.154811 0.205720 0.688071
H -5.263002 -0.782741 1.331014
H -4.549301 -1.979781 0.185841
H -6.197838 -1.336045 -0.094758
SCF Energy = -1057.74992905
Number of imaginary frequencies = 0

Compound 1_c13
B3LYP/6-31G* Geometry
C -2.352108 2.049910 -0.710543
C -1.022885 -0.001675 -0.369096
N -3.340999 0.136934 0.243330
C -2.139236 -0.634646 0.305222
C -3.444838 1.415675 -0.206571
C -1.125959 1.325058 -0.748641
O -0.061098 2.021851 -1.195219
C 1.305032 1.669632 -0.792711
C 1.401491 0.376344 0.079392
C 0.359854 -0.617333 -0.567871
C 0.538203 -2.112978 -0.208336
C 2.011866 -2.528348 -0.349777
C 3.007299 -1.637660 0.399346
C 2.853332 -0.188077 -0.086313
O -2.170354 -1.726886 0.873878
C 1.943814 2.985887 -0.308351
C 1.191573 3.785170 0.763351
C 1.089679 0.589375 1.574713
H 0.554908 -0.564871 -1.653530
C 4.446514 -2.140846 0.217472
C -0.307985 -3.015026 -1.122620
O 3.842307 0.667371 0.499951
O -4.452619 -0.346936 0.923686
C -5.072210 -1.429500 0.207187
H -2.394278 3.075467 -1.053454
H -4.427014 1.860604 -0.106166
H 1.794413 1.415662 -1.740702
H 0.215096 -2.280544 0.823779
H 2.121780 -3.567183 -0.011733
H 2.286083 -2.528881 -1.417416
H 2.767719 -1.672321 1.474469
H 3.099474 -0.174877 -1.157101
H 2.961299 2.754709 0.018036
H 2.032509 3.623014 -1.197960
H 1.682972 4.753874 0.909398
H 0.159187 3.980216 0.456984
H 1.166798 3.283539 1.733903
H 0.143814 1.113218 1.723965
H 1.867637 1.181419 2.066621
H 1.010336 -0.369469 2.096774
H 4.546790 -3.164326 0.596853
H 4.723736 -2.149599 -0.844457
H 5.167282 -1.506127 0.740225
H -0.110032 -4.070130 -0.897518
H -0.051132 -2.849362 -2.177911
H -1.375425 -2.844683 -0.984009
H 3.803652 0.542959 1.461498
H -4.391913 -2.280745 0.143742
H -5.389003 -1.103005 -0.790840
H -5.946199 -1.686062 0.809759
SCF Energy = -1057.75435311
Number of imaginary frequencies = 0

Compound 1_c14
B3LYP/6-31G* Geometry
C -2.311697 2.149927 -0.572451
C -1.045709 0.040968 -0.349016
N -3.363593 0.220421 0.268091
C -2.186086 -0.589940 0.288221
C -3.425079 1.526022 -0.104238
C -1.109198 1.389155 -0.657490
O -0.033406 2.095226 -1.060236
C 1.332443 1.623461 -0.829629
C 1.397666 0.343741 0.062345
C 0.316588 -0.616686 -0.576261
C 0.447956 -2.122053 -0.230487
C 1.911107 -2.584358 -0.339663
C 2.904016 -1.716041 0.435702
C 2.813696 -0.276110 -0.097436
O -2.257020 -1.709525 0.797049
C 2.127543 2.893244 -0.480621
C 1.677454 3.703838 0.740435
C 1.098941 0.591702 1.557301
H 0.495392 -0.568419 -1.664270
C 4.330125 -2.276676 0.331435
C -0.401383 -2.989228 -1.175459
O 3.753174 0.587200 0.554973
O -4.490968 -0.266459 0.919874
C -5.144658 -1.283069 0.140574
H -2.317523 3.194579 -0.854755
H -4.391900 1.996965 0.022918
H 1.685960 1.303879 -1.819300
H 0.098435 -2.292842 0.791997
H 1.978845 -3.624698 0.004920
H 2.210744 -2.598336 -1.400761
H 2.622679 -1.705818 1.497339
H 3.047489 -0.312455 -1.177725
H 3.177212 2.608916 -0.379261
H 2.048844 3.537790 -1.366371
H 2.198458 4.668083 0.752463
H 0.602456 3.908881 0.706487
H 1.901863 3.199327 1.683046
H 0.267757 1.285761 1.696320
H 1.976342 0.998721 2.062910
H 0.822761 -0.343297 2.055573
H 4.356806 -3.321279 0.660830
H 4.695849 -2.250712 -0.704454
H 5.038591 -1.726985 0.962857
H -0.249925 -4.051705 -0.948555
H -0.103276 -2.829679 -2.220965
H -1.465862 -2.780949 -1.071627
H 4.640079 0.247862 0.364071
H -5.451713 -0.886895 -0.835122
H -6.026035 -1.548561 0.728393
H -4.491785 -2.150011 0.023570

SCF Energy = -1057.75410006
Number of imaginary frequencies = 0

Compound 1_c15
B3LYP/6-31G* Geometry
C 2.389328 2.055818 -0.886589
C 1.096941 0.077904 -0.174348
N 3.507410 0.128074 -0.133463
C 2.328288 -0.566832 0.258175
C 3.546546 1.372097 -0.683782
C 1.162157 1.408881 -0.550490
O 0.068187 2.204633 -0.589373
C -1.049227 1.730453 0.217446
C -1.441915 0.334339 -0.336886
C -0.242070 -0.625123 0.031169
C -0.489758 -2.001345 -0.677108
C -1.738799 -2.608317 -0.006178
C -2.992736 -1.720408 -0.046488
C -2.714141 -0.249592 0.341213
O 2.429016 -1.588015 0.934577
C -2.058157 2.889414 0.234811
C -2.788925 3.058153 1.572127
C -1.659809 0.476879 -1.866374
H -0.316192 -0.834002 1.110975
C -4.123413 -2.308039 0.809657
C 0.629285 -3.050600 -0.701873
O -3.884514 0.537979 0.092626
O 4.728782 -0.502603 0.061075
C 5.151309 -0.422101 1.436152
H 2.383432 3.070752 -1.262446
H 4.537099 1.745694 -0.912267
H -0.658492 1.597704 1.238415
H -0.724260 -1.792984 -1.727758
H -1.978590 -3.572268 -0.474133
H -1.487268 -2.835575 1.041237
H -3.346045 -1.703171 -1.090646
H -2.576693 -0.191544 1.429259
H -1.476637 3.796180 0.030448
H -2.787548 2.790615 -0.573228
H -3.429062 2.200596 1.792055
H -2.078706 3.189803 2.398149
H -3.428682 3.947379 1.542816
H -0.711353 0.573428 -2.399648
H -2.248176 1.371483 -2.093072
H -2.185540 -0.378458 -2.297701
H -4.395872 -3.311142 0.461528
H -3.811907 -2.393825 1.858586
H -5.014191 -1.674418 0.774518
H 0.261731 -3.933359 -1.241823
H 0.936576 -3.355601 0.299623
H 1.520269 -2.693256 -1.223709
H -4.129537 0.405762 -0.837213
H 4.445913 -0.949102 2.080248
H 5.261892 0.624492 1.746461
H 6.124780 -0.917141 1.448530
SCF Energy = -1057.75099680
Number of imaginary frequencies = 0

Compound 1_c16
B3LYP/6-31G* Geometry
C 2.392268 2.046911 -0.896451
C 1.095193 0.074557 -0.178166
N 3.505302 0.120939 -0.131629
C 2.324145 -0.570225 0.260144
C 3.547811 1.362305 -0.688247
C 1.163298 1.403874 -0.558778
O 0.071654 2.201131 -0.599670
C -1.047434 1.733805 0.208252
C -1.445267 0.335450 -0.341522
C -0.244686 -0.625288 0.027809
C -0.491314 -2.000928 -0.681225
C -1.743848 -2.606857 -0.016192
C -2.993742 -1.713018 -0.060440
C -2.703148 -0.249106 0.348200
O 2.421645 -1.588268 0.942267
C -2.053085 2.896082 0.216202
C -2.760899 3.089471 1.562103
C -1.674296 0.473385 -1.868579
H -0.316957 -0.834616 1.107498
C -4.121268 -2.323233 0.788342
C 0.626156 -3.051988 -0.699741
O -3.823637 0.578280 0.015050
O 4.725482 -0.509606 0.071358
C 5.143033 -0.419468 1.447153
H 2.388593 3.059739 -1.278004
H 4.539492 1.732761 -0.916973
H -0.656818 1.604540 1.230026
H -0.721302 -1.791717 -1.732180
H -1.985718 -3.567308 -0.490499
H -1.494901 -2.841210 1.030631
H -3.348513 -1.673091 -1.098637
H -2.532506 -0.217010 1.439514
H -1.473717 3.798367 -0.012294
H -2.794480 2.779420 -0.577205
H -3.420357 3.963809 1.522586
H -3.377094 2.223125 1.815919
H -2.039898 3.254939 2.372530
H -0.729237 0.596317 -2.403159
H -2.298519 1.343707 -2.083674
H -2.185240 -0.392079 -2.292477
H -4.323281 -3.354197 0.476716
H -3.850235 -2.345755 1.851837
H -5.068138 -1.776218 0.690472
H 0.259666 -3.935268 -1.239696
H 0.929781 -3.355694 0.303351
H 1.519318 -2.696658 -1.219149
H -4.599858 0.217922 0.469273
H 4.434413 -0.940629 2.092510
H 5.253944 0.629272 1.750172
H 6.115796 -0.915721 1.467095
SCF Energy = -1057.75007254
Number of imaginary frequencies = 0

Compound 1_c17
B3LYP/6-31G* Geometry
C -2.438107 1.905100 -0.887351
C -1.016681 -0.081999 -0.531219
N -3.374010 -0.090805 -0.065730
C -2.146052 -0.817701 0.001852
C -3.526234 1.188587 -0.496863
C -1.170900 1.253396 -0.858660
O -0.124379 2.037336 -1.187763
C 1.242001 1.717284 -0.766561
C 1.347720 0.409619 0.083015
C 0.408170 -0.616829 -0.665287
C 0.640672 -2.114936 -0.344009
C 2.141798 -2.450117 -0.358477
C 3.004016 -1.525689 0.504495
C 2.821440 -0.082154 0.007713
O -2.161619 -1.946058 0.496944
C 1.820497 3.049666 -0.254473
C 1.067050 3.765234 0.873202
C 0.924793 0.567344 1.559184
H 0.667427 -0.517227 -1.733771
C 4.477763 -1.958264 0.475573
C -0.067013 -3.022454 -1.364790
O 3.636171 0.837851 0.745171
O -4.541608 -0.768471 0.259338
C -4.694018 -0.895720 1.685484
H -2.515763 2.936875 -1.204328
H -4.543559 1.560063 -0.490787
H 1.764156 1.493647 -1.705764
H 0.235434 -2.340291 0.647041
H 2.274143 -3.490812 -0.034574
H 2.512571 -2.408168 -1.395900
H 2.659848 -1.577072 1.546171
H 3.128457 -0.063010 -1.054428
H 2.858549 2.869099 0.033727
H 1.837319 3.717167 -1.126304
H 1.483323 4.769690 1.011633
H 0.005248 3.877852 0.631159
H 1.147227 3.244175 1.829854
H 0.000346 1.139881 1.657574
H 1.706084 1.069333 2.133265

H 0.746934 -0.412445 2.013938
H 4.575868 -3.006997 0.777207
H 4.902452 -1.867408 -0.533789
H 5.095663 -1.370837 1.165449
H 0.159276 -4.074621 -1.152352
H 0.285719 -2.808659 -2.383252
H -1.150043 -2.907113 -1.330231
H 4.560584 0.586585 0.601439
H -4.741186 0.092684 2.159504
H -3.880000 -1.491514 2.101275
H -5.648016 -1.412711 1.809858
SCF Energy = -1057.75391589
Number of imaginary frequencies = 0

Compound 1_c18
B3LYP/6-31G* Geometry
C -2.447246 1.876923 -0.921146
C -1.012559 -0.093378 -0.528603
N -3.371697 -0.114631 -0.075124
C -2.137645 -0.828406 0.014153
C -3.531987 1.156279 -0.528253
C -1.174784 1.236723 -0.874188
O -0.130578 2.023119 -1.205391
C 1.231838 1.721290 -0.757398
C 1.347831 0.415876 0.093106
C 0.416200 -0.619958 -0.650484
C 0.662110 -2.113453 -0.322282
C 2.165847 -2.432752 -0.363997
C 3.047055 -1.507012 0.479419
C 2.839806 -0.055641 0.019889
O -2.143764 -1.945959 0.533240
C 1.789341 3.055404 -0.225500
C 0.967708 3.790075 0.841630
C 0.917773 0.572331 1.565715
H 0.680242 -0.522870 -1.718101
C 4.523807 -1.918782 0.393515
C -0.052207 -3.034354 -1.326664
O 3.730617 0.838104 0.699466
O -4.535559 -0.798169 0.250939
C -4.700647 -0.903309 1.677460
H -2.532053 2.902958 -1.254552
H -4.552671 1.518364 -0.536180
H 1.772288 1.506505 -1.687599
H 0.274451 -2.336213 0.676754
H 2.316200 -3.472990 -0.046881
H 2.515086 -2.383142 -1.408129
H 2.730766 -1.586244 1.532390
H 3.160428 0.004455 -1.029386
H 2.806763 2.868081 0.127403
H 1.869805 3.716427 -1.098283
H 1.399385 4.781953 1.017463
H -0.066893 3.931744 0.513826
H 0.950052 3.268309 1.801779
H -0.054799 1.059765 1.656685
H 1.634655 1.175396 2.131105
H 0.835560 -0.404865 2.052359
H 4.657946 -2.945350 0.753514
H 4.877862 -1.880079 -0.644579
H 5.165157 -1.258707 0.984011
H 0.192200 -4.082369 -1.114080
H 0.280105 -2.820424 -2.351873
H -1.135790 -2.933098 -1.273707
H 3.644177 0.670398 1.651171
H -4.761902 0.092278 2.134586
H -3.885346 -1.484621 2.110851
H -5.650976 -1.427145 1.801061
SCF Energy = -1057.75402376
Number of imaginary frequencies = 0

Compound 1_c19
B3LYP/6-31G* Geometry
C -2.312868 2.166577 0.577165
C -1.082855 0.079771 0.096381
N -3.492747 0.172414 0.176045
C -2.344995 -0.594159 -0.173811
C -3.487564 1.484091 0.537748
C -1.113676 1.454144 0.274793
O -0.012290 2.229222 0.135831
C 1.056365 1.607064 -0.625424
C 1.461631 0.307612 0.118543
C 0.236634 -0.673956 -0.076106
C 0.503604 -1.956083 0.784613
C 1.728950 -2.648984 0.151199
C 2.990071 -1.777581 0.032471
C 2.700631 -0.368346 -0.530863
O -2.496652 -1.697906 -0.692230
C 2.045278 2.729645 -0.971321
C 2.701001 3.517302 0.168592
C 1.725239 0.630246 1.611929
H 0.259600 -1.017418 -1.123220
C 4.082634 -2.481854 -0.784117
C -0.612731 -2.993813 0.966808
O 3.880762 0.440281 -0.444421
O -4.730933 -0.454009 0.131514
C -5.222709 -0.555078 -1.219013
H -2.273876 3.225153 0.799158
H -4.459664 1.906819 0.759826
H 0.613931 1.294543 -1.584434
H 0.769333 -1.628841 1.796902
H 1.980838 -3.549915 0.726112
H 1.438863 -3.002192 -0.850406
H 3.384682 -1.630471 1.051367
H 2.512876 -0.451239 -1.610402
H 2.819489 2.305966 -1.616630
H 1.467397 3.433090 -1.585504
H 3.176770 4.418768 -0.234225
H 1.965136 3.833799 0.914652
H 3.481280 2.935896 0.666868
H 0.793222 0.825730 2.148082
H 2.348482 1.518795 1.717902
H 2.227566 -0.188395 2.132859
H 4.360941 -3.436697 -0.323170
H 3.730806 -2.694050 -1.801855
H 4.978851 -1.859652 -0.861934
H -0.223559 -3.810179 1.589892
H -0.952429 -3.410888 0.017277
H -1.486949 -2.579685 1.473861
H 4.176466 0.420141 0.479791
H -4.558064 -1.179127 -1.818159
H -5.336065 0.440967 -1.665013
H -6.201631 -1.028093 -1.114982
SCF Energy = -1057.74946488
Number of imaginary frequencies = 0

Compound 1_c20
B3LYP/6-31G* Geometry
C -2.314643 2.163030 0.586110
C -1.082535 0.080099 0.094981
N -3.492179 0.169601 0.175347
C -2.343636 -0.593494 -0.179258
C -3.488459 1.479341 0.544497
C -1.114675 1.453583 0.279139
O -0.015043 2.229723 0.141419
C 1.054419 1.612080 -0.621971
C 1.462886 0.310449 0.120497
C 0.237222 -0.672021 -0.078676
C 0.500469 -1.954885 0.781721
C 1.732270 -2.644889 0.158175
C 2.989951 -1.767018 0.052491
C 2.691406 -0.367182 -0.531561
O -2.494473 -1.694398 -0.704585
C 2.038194 2.740021 -0.964279
C 2.713982 3.506025 0.178386
C 1.729913 0.628971 1.613098
H 0.259714 -1.014218 -1.126113
C 4.086363 -2.490467 -0.745858
C -0.614425 -2.996057 0.952881
O 3.823943 0.492101 -0.355101
O -4.729487 -0.458671 0.129396
C -5.222767 -0.553960 -1.220791
H -2.276560 3.220346 0.814192
H -4.460781 1.899483 0.770471
H 0.610953 1.301431 -1.581417
H 0.757739 -1.627888 1.795741

H 1.982895 -3.543291 0.737787
H 1.450040 -3.002303 -0.844571
H 3.377922 -1.598343 1.065534
H 2.479149 -0.475430 -1.611305
H 2.801435 2.322685 -1.626940
H 1.452008 3.451435 -1.561623
H 3.194752 4.407547 -0.219465
H 1.986804 3.823324 0.932755
H 3.488011 2.904473 0.660380
H 0.800569 0.858698 2.140799
H 2.393467 1.486132 1.712248
H 2.206072 -0.202950 2.133950
H 4.294159 -3.475252 -0.312379
H 3.781397 -2.645270 -1.788975
H 5.037593 -1.942319 -0.747273
H -0.227171 -3.813821 1.575480
H -0.947730 -3.410568 -0.000123
H -1.492497 -2.585986 1.456341
H 4.577125 0.082036 -0.805836
H -4.558120 -1.174531 -1.823565
H -5.337663 0.443992 -1.662156
H -6.201161 -1.028419 -1.117909
SCF Energy = -1057.74849991
Number of imaginary frequencies = 0

Compound 1_c21
B3LYP/6-31G* Geometry
C -2.294483 2.256861 0.078058
C -1.063167 0.136604 -0.222334
N -3.471024 0.229113 -0.080827
C -2.322936 -0.583127 -0.316011
C -3.471492 1.579298 0.072533
C -1.087668 1.514430 -0.090623
O 0.018493 2.286589 -0.090757
C 1.320516 1.705027 -0.379958
C 1.423172 0.301619 0.291010
C 0.291456 -0.557382 -0.390783
C 0.326080 -2.093719 -0.057992
C 1.532806 -2.527568 0.805129
C 2.884894 -1.833916 0.514683
C 2.746535 -0.410701 -0.088376
O -2.494828 -1.781467 -0.546176
C 2.310683 2.802433 0.018941
C 2.079509 4.113424 -0.743696
C 1.279286 0.464381 1.820758
H 0.530837 -0.498828 -1.464441
C 3.820647 -2.698109 -0.349941
C 0.250563 -2.906241 -1.363083
O 3.866263 0.380928 0.327481
O -4.720185 -0.355718 -0.251657
C -5.058319 -1.197238 0.865091
H -2.255504 3.332603 0.188611
H -4.449643 2.033461 0.170358
H 1.371514 1.567352 -1.470702
H -0.570404 -2.343462 0.510697
H 1.286249 -2.369706 1.859574
H 1.654384 -3.613420 0.700736
H 3.383505 -1.679038 1.480749
H 2.759620 -0.490752 -1.189720
H 2.228809 2.981012 1.097662
H 3.319357 2.431709 -0.164137
H 2.822996 4.862210 -0.448290
H 2.173253 3.964336 -1.827137
H 1.085571 4.525116 -0.547760
H 0.528521 1.219189 2.068068
H 2.232336 0.785160 2.251532
H 0.980208 -0.462598 2.312126
H 4.035679 -3.652707 0.143058
H 3.374668 -2.919180 -1.326301
H 4.787116 -2.207596 -0.534978
H 0.222967 -3.981503 -1.149189
H 1.117912 -2.716117 -2.009450
H -0.657477 -2.653118 -1.915313
H 4.664874 -0.146645 0.171822
H -5.075491 -0.617623 1.796253
H -4.358960 -2.032247 0.935777
H -6.062167 -1.560508 0.634620
SCF Energy = -1057.74765688
Number of imaginary frequencies = 0

Compound 1_c22
B3LYP/6-31G* Geometry
C -2.314491 2.069359 -0.929649
C -1.070256 0.015626 -0.369438
N -3.367319 0.302571 0.229395
C -2.201360 -0.514382 0.361015
C -3.426320 1.530221 -0.354918
C -1.124229 1.292993 -0.891757
O 0.002140 1.826703 -1.424278
C 1.241024 1.710628 -0.628467
C 1.356003 0.322768 0.119329
C 0.291134 -0.637976 -0.533999
C 0.433799 -2.130356 -0.152858
C 1.888450 -2.591638 -0.357010
C 2.954086 -1.710175 0.306578
C 2.776991 -0.256651 -0.159963
O -2.268954 -1.551710 1.021217
C 1.314161 3.020805 0.180756
C 2.696347 3.462130 0.675807
C 1.095470 0.445049 1.634438
H 0.500840 -0.592236 -1.616611
C 4.367117 -2.228563 0.003449
C -0.484699 -3.020521 -1.006566
O 3.811927 0.594342 0.339802
O -4.498184 -0.069396 0.946970
C -5.160152 -1.190431 0.333706
H -2.322801 3.054978 -1.376468
H -4.390601 2.019217 -0.293662
H 2.012809 1.743663 -1.402653
H 0.152545 -2.268010 0.896329
H 1.989965 -3.623725 0.003656
H 2.099782 -2.628696 -1.437990
H 2.808042 -1.744536 1.397818
H 2.928703 -0.240244 -1.248319
H 0.936567 3.793028 -0.499576
H 0.605152 2.982209 1.016457
H 3.094234 2.818743 1.462930
H 3.428805 3.461653 -0.138091
H 2.632292 4.480924 1.075122
H 0.112124 0.878298 1.834769
H 1.835447 1.081218 2.130362
H 1.113251 -0.533026 2.122944
H 4.488967 -3.254722 0.369222
H 4.555530 -2.234931 -1.077837
H 5.134307 -1.602364 0.467207
H -0.293555 -4.078360 -0.788354
H -0.294149 -2.865138 -2.077179
H -1.537666 -2.827148 -0.800082
H 3.827338 0.499177 1.305376
H -5.464527 -0.946955 -0.691482
H -6.042879 -1.354578 0.955331
H -4.513177 -2.069265 0.352620
SCF Energy = -1057.75318699
Number of imaginary frequencies = 0

Compound 1_c23
B3LYP/6-31G* Geometry
C -2.314999 2.191584 0.316264
C -1.065036 0.088760 -0.030381
N -3.458610 0.140646 0.278585
C -2.307769 -0.663324 0.033966
C -3.472780 1.490538 0.429341
C -1.111382 1.469548 0.058835
O -0.028817 2.264799 -0.066357
C 1.258288 1.700008 -0.442770
C 1.452217 0.327949 0.271556
C 0.289083 -0.582587 -0.278461
C 0.388117 -2.102401 0.110077
C 1.677188 -2.477757 0.875200
C 2.981016 -1.768287 0.439922
C 2.757117 -0.369112 -0.193371
O -2.468358 -1.876133 -0.116656
C 2.250457 2.834852 -0.175615
C 1.924501 4.103897 -0.973749

C 1.435367 0.550524 1.799777
H 0.440162 -0.563105 -1.369590
C 3.853483 -2.643107 -0.478155
C 0.212746 -2.969485 -1.149666
O 3.887438 0.461242 0.100410
O -4.666218 -0.509656 0.495764
C -5.242086 -0.972315 -0.739939
H -2.287968 3.269171 0.411329
H -4.443514 1.923535 0.636537
H 1.219968 1.518290 -1.527751
H -0.446266 -2.346275 0.768294
H 1.521215 -2.285955 1.941234
H 1.814769 -3.564117 0.799719
H 3.561295 -1.571574 1.351201
H 2.679946 -0.485792 -1.289088
H 2.252564 3.061523 0.897136
H 3.249553 2.477260 -0.425241
H 2.672490 4.880329 -0.777833
H 1.928871 3.905748 -2.053406
H 0.941529 4.504863 -0.711316
H 0.667014 1.273555 2.084885
H 2.402919 0.941917 2.127108
H 1.237324 -0.368180 2.353608
H 4.141358 -3.571692 0.027162
H 3.321810 -2.913898 -1.397270
H 4.783242 -2.136654 -0.774428
H 0.230497 -4.035198 -0.891602
H 1.011664 -2.789307 -1.881931
H -0.748297 -2.758483 -1.624654
H 4.683542 -0.055019 -0.099845
H -4.591611 -1.713952 -1.206241
H -5.430587 -0.130866 -1.418249
H -6.188746 -1.428349 -0.442138
SCF Energy = -1057.74740265
Number of imaginary frequencies = 0

Compound 1_c24
B3LYP/6-31G* Geometry
C -2.310931 2.080447 -0.907499
C -1.070403 0.019303 -0.366368
N -3.365775 0.306591 0.238427
C -2.202567 -0.516427 0.357926
C -3.422526 1.539978 -0.333814
C -1.122812 1.300208 -0.879956
O 0.002172 1.833810 -1.414822
C 1.245519 1.711290 -0.625497
C 1.355283 0.320874 0.121062
C 0.289460 -0.634977 -0.538893
C 0.427449 -2.131141 -0.169034
C 1.882999 -2.595980 -0.361106
C 2.933118 -1.715601 0.326326
C 2.763215 -0.266578 -0.158592
O -2.274756 -1.563293 1.002406
C 1.328871 3.018232 0.187768
C 2.719872 3.458424 0.658058
C 1.103777 0.443527 1.637415
H 0.496418 -0.581730 -1.621720
C 4.351940 -2.241001 0.060071
C -0.486700 -3.011852 -1.036837
O 3.725364 0.613955 0.426637
O -4.496981 -0.070330 0.952898
C -5.164783 -1.179636 0.325230
H -2.317294 3.070138 -1.345243
H -4.384888 2.031722 -0.264655
H 2.012488 1.740800 -1.404981
H 0.137771 -2.275686 0.876683
H 1.977257 -3.629999 -0.003804
H 2.106886 -2.628660 -1.440095
H 2.768835 -1.736031 1.411419
H 2.900079 -0.272639 -1.255953
H 0.931875 3.793007 -0.478718
H 0.639987 2.968950 1.039066
H 2.652606 4.453284 1.113752
H 3.157843 2.771699 1.383264
H 3.416581 3.523108 -0.185861
H 0.138555 0.914405 1.841558
H 1.885177 1.031414 2.123620
H 1.081986 -0.539419 2.115243
H 4.439250 -3.289453 0.366339
H 4.603461 -2.190074 -1.008450
H 5.109862 -1.680749 0.620723
H -0.302281 -4.072052 -0.824027
H -0.286463 -2.850078 -2.104804
H -1.540613 -2.815280 -0.838062
H 4.602678 0.340333 0.120563
H -4.521213 -2.061153 0.329741
H -5.470793 -0.920208 -0.695625
H -6.046705 -1.349459 0.946526
SCF Energy = -1057.75333182
Number of imaginary frequencies = 0

Compound 1_c25
B3LYP/6-31G* Geometry
C -2.297893 2.257624 0.084982
C -1.067117 0.137376 -0.218045
N -3.475317 0.230538 -0.079904
C -2.327200 -0.581913 -0.314908
C -3.475247 1.580366 0.075924
C -1.091231 1.515118 -0.083448
O 0.016248 2.287092 -0.078063
C 1.316242 1.704308 -0.374500
C 1.421644 0.300519 0.291008
C 0.287314 -0.557204 -0.387451
C 0.324581 -2.095396 -0.062696
C 1.532499 -2.534235 0.797558
C 2.884654 -1.834050 0.516365
C 2.747992 -0.417780 -0.097776
O -2.498503 -1.779633 -0.547067
C 2.311747 2.797678 0.022426
C 2.093733 4.105856 -0.748681
C 1.275665 0.459184 1.820127
H 0.526496 -0.494033 -1.460799
C 3.848971 -2.683233 -0.326841
C 0.253414 -2.899757 -1.373050
O 3.941863 0.322248 0.180406
O -4.724461 -0.352884 -0.255049
C -5.066076 -1.197836 0.858150
H -2.258973 3.333204 0.197115
H -4.453368 2.034877 0.172424
H 1.363983 1.569789 -1.465499
H -0.572275 -2.349774 0.503332
H 1.280245 -2.394263 1.853957
H 1.659495 -3.617772 0.678689
H 3.369458 -1.681564 1.493964
H 2.752646 -0.502309 -1.192373
H 2.222472 2.987129 1.099503
H 3.318167 2.416854 -0.156796
H 2.837220 4.853332 -0.450302
H 2.197804 3.949686 -1.829864
H 1.099330 4.521697 -0.564022
H 0.462993 1.144602 2.072421
H 2.190040 0.880067 2.255866
H 1.073623 -0.487405 2.322128
H 4.088727 -3.624567 0.180990
H 3.409821 -2.934064 -1.299797
H 4.783649 -2.141374 -0.505682
H 0.220380 -3.975931 -1.165190
H 1.126476 -2.709859 -2.011608
H -0.650008 -2.640246 -1.930020
H 4.078039 0.308125 1.141472
H -5.085209 -0.621193 1.791123
H -4.367710 -2.033730 0.927825
H -6.069621 -1.559363 0.623800
SCF Energy = -1057.74862661
Number of imaginary frequencies = 0

Compound 1_c26
B3LYP/6-31G* Geometry
C -2.315032 2.191538 0.316105
C -1.065060 0.088668 -0.030419
N -3.458615 0.140596 0.278786
C -2.307834 -0.663401 0.033840
C -3.472820 1.490493 0.429340
C -1.111443 1.469438 0.058771

O -0.028872 2.264719 -0.066414
C 1.258207 1.699969 -0.442815
C 1.452186 0.327955 0.271617
C 0.289119 -0.582703 -0.278430
C 0.388305 -2.102511 0.110196
C 1.677426 -2.477570 0.875344
C 2.981196 -1.768191 0.439800
C 2.757219 -0.368930 -0.193269
O -2.468585 -1.876134 -0.117016
C 2.250342 2.834846 -0.175750
C 1.924227 4.103895 -0.973823
C 1.435259 0.550624 1.799824
H 0.440295 -0.563257 -1.369539
C 3.853515 -2.642925 -0.478543
C 0.212987 -2.969749 -1.149445
O 3.887438 0.461497 0.100684
O -4.666224 -0.509702 0.495852
C -5.242044 -0.972333 -0.739841
H -2.288011 3.269122 0.411132
H -4.443546 1.923496 0.636555
H 1.219909 1.518148 -1.527786
H -0.446027 -2.346453 0.768434
H 1.521515 -2.285396 1.941308
H 1.815086 -3.563952 0.800191
H 3.561682 -1.571623 1.350990
H 2.680121 -0.485453 -1.289026
H 2.252666 3.061482 0.896999
H 3.249408 2.477289 -0.425572
H 2.672062 4.880429 -0.777710
H 1.928823 3.905800 -2.053483
H 0.941142 4.504631 -0.711497
H 0.666885 1.273670 2.084827
H 2.402807 0.942025 2.127138
H 1.237187 -0.368053 2.353693
H 4.141169 -3.571701 0.026541
H 3.321822 -2.913290 -1.397765
H 4.783367 -2.136511 -0.774580
H 0.231073 -4.035421 -0.891242
H 1.011698 -2.789463 -1.881896
H -0.748221 -2.759075 -1.624236
H 4.683631 -0.055065 -0.098435
H -4.591594 -1.713996 -1.206153
H -5.430507 -0.130897 -1.418181
H -6.188714 -1.428371 -0.442069
SCF Energy = -1057.74740264
Number of imaginary frequencies = 0

Compound 1_c27
B3LYP/6-31G* Geometry
C -2.317926 2.193029 0.319190
C -1.068773 0.089654 -0.027824
N -3.462750 0.142522 0.278981
C -2.312235 -0.662061 0.034792
C -3.476361 1.492367 0.429810
C -1.114434 1.470506 0.063213
O -0.030043 2.265691 -0.056705
C 1.255109 1.699144 -0.437074
C 1.450020 0.326614 0.272268
C 0.285244 -0.582668 -0.275714
C 0.385406 -2.104539 0.105093
C 1.675382 -2.485057 0.867581
C 2.980236 -1.769702 0.441401
C 2.758262 -0.376168 -0.200182
O -2.472753 -1.874488 -0.116579
C 2.252079 2.829661 -0.170107
C 1.941109 4.095493 -0.979037
C 1.428967 0.544860 1.800282
H 0.437266 -0.558674 -1.366513
C 3.882245 -2.626581 -0.460655
C 0.212658 -2.964300 -1.159926
O 3.951940 0.398997 -0.043850
O -4.671117 -0.506922 0.493932
C -5.244930 -0.970322 -0.742623
H -2.290708 3.270601 0.414371
H -4.447371 1.925556 0.635238
H 1.215900 1.520160 -1.522202
H -0.449384 -2.352268 0.761321
H 1.514246 -2.309402 1.936355
H 1.816674 -3.569697 0.778571
H 3.549526 -1.578106 1.365031
H 2.675483 -0.494989 -1.288724
H 2.243599 3.067399 0.901011
H 3.249976 2.461320 -0.411815
H 2.689400 4.870430 -0.778990
H 1.958189 3.889931 -2.056865
H 0.956276 4.500572 -0.730198
H 0.604576 1.197376 2.097826
H 2.353202 1.032260 2.134056
H 1.325190 -0.387559 2.355859
H 4.190443 -3.544783 0.052448
H 3.361139 -2.919496 -1.379743
H 4.782953 -2.071230 -0.742805
H 0.223450 -4.031087 -0.906388
H 1.018005 -2.785349 -1.885185
H -0.744281 -2.746480 -1.640262
H 4.167017 0.417653 0.902560
H -4.594004 -1.712561 -1.207278
H -5.431858 -0.129258 -1.421797
H -6.192217 -1.425766 -0.446058
SCF Energy = -1057.74846934
Number of imaginary frequencies = 0

Compound 1_c28
B3LYP/6-31G* Geometry
C -2.415794 1.850763 -1.161367
C -1.058700 -0.108556 -0.527853
N -3.403107 0.007363 -0.069444
C -2.200338 -0.748731 0.088140
C -3.520882 1.221463 -0.671653
C -1.174096 1.168696 -1.039807
O -0.058842 1.795124 -1.486965
C 1.133443 1.753496 -0.616270
C 1.297900 0.364628 0.122833
C 0.354352 -0.659843 -0.616130
C 0.580911 -2.146714 -0.250944
C 2.078085 -2.496193 -0.344959
C 3.009824 -1.547858 0.418678
C 2.761685 -0.110136 -0.066835
O -2.241794 -1.812137 0.708036
C 1.064311 3.051272 0.213368
C 2.383499 3.589870 0.778535
C 0.938099 0.445798 1.620247
H 0.629506 -0.576351 -1.681628
C 4.479163 -1.960832 0.245451
C -0.199218 -3.082516 -1.189410
O 3.613478 0.833843 0.586371
O -4.591521 -0.584107 0.339101
C -4.734211 -0.539024 1.771178
H -2.469840 2.831447 -1.615761
H -4.528006 1.617890 -0.710079
H 1.946523 1.851040 -1.341596
H 0.231652 -2.328051 0.770576
H 2.226438 -3.524568 0.010133
H 2.376492 -2.498336 -1.406196
H 2.774948 -1.593573 1.490109
H 2.969611 -0.092668 -1.152872
H 0.652382 3.802944 -0.470239
H 0.327483 2.937103 1.017093
H 2.209090 4.568809 1.240338
H 2.826944 2.927231 1.522583
H 3.125749 3.722825 -0.017076
H -0.070459 0.842735 1.763618
H 1.640203 1.081095 2.164452
H 0.956794 -0.543752 2.084676
H 4.624199 -3.004988 0.544234
H 4.798373 -1.872488 -0.802254
H 5.152287 -1.355626 0.864480
H 0.047472 -4.128340 -0.968516
H 0.068979 -2.895006 -2.238100
H -1.276809 -2.966950 -1.070817
H 4.527525 0.626665 0.341487
H -4.738117 0.498781 2.127067
H -3.940146 -1.114943 2.249089
H -5.706278 -0.999754 1.959746

SCF Energy = -1057.75300439
Number of imaginary frequencies = 0

Compound 1_c29
B3LYP/6-31G* Geometry
C -2.074661 2.337901 0.537849
C -0.951995 0.192274 0.062004
N -3.339642 0.475396 -0.152133
C -2.210616 -0.345805 -0.436440
C -3.279575 1.766148 0.279292
C -0.902237 1.545201 0.352596
O 0.264123 2.225721 0.440184
C 1.369549 1.606367 -0.268245
C 1.598265 0.196755 0.330031
C 0.316078 -0.657612 -0.007517
C 0.354820 -1.951215 0.875345
C 1.776638 -2.607133 0.786322
C 2.712777 -2.075334 -0.321112
C 2.781969 -0.514438 -0.376450
O -2.370173 -1.397455 -1.051830
C 2.512379 2.619873 -0.227351
C 2.187063 3.903638 -1.000125
C 1.849694 0.358332 1.849428
H 0.402401 -0.980907 -1.053693
C 2.419945 -2.706221 -1.695992
C -0.714934 -3.023406 0.615556
O 3.999679 -0.032588 0.205950
O -4.572876 0.081396 -0.663222
C -5.125282 -1.010580 0.090751
H -1.987324 3.375643 0.832620
H -4.234844 2.269994 0.357090
H 1.044738 1.483060 -1.314296
H 0.208713 -1.618465 1.909897
H 2.288013 -2.498428 1.748803
H 1.663544 -3.688074 0.639708
H 3.727111 -2.398736 -0.046833
H 2.753855 -0.216283 -1.439392
H 2.737498 2.865167 0.816227
H 3.410808 2.144781 -0.630817
H 3.021497 4.611470 -0.944493
H 1.999220 3.693885 -2.060745
H 1.297831 4.394749 -0.593382
H 0.960694 0.748547 2.351811
H 2.671546 1.054880 2.022293
H 2.130132 -0.577088 2.331643
H 2.523308 -3.796332 -1.644785
H 1.407906 -2.492913 -2.055039
H 3.122821 -2.341475 -2.456079
H -0.505376 -3.886999 1.260525
H -0.714029 -3.364133 -0.423924
H -1.723238 -2.675908 0.839844
H 4.733503 -0.443195 -0.277094
H -5.245259 -0.733895 1.145186
H -4.498983 -1.899027 -0.011928
H -6.103882 -1.183150 -0.362532
SCF Energy = -1057.74202154
Number of imaginary frequencies = 0

Compound 1_c30
B3LYP/6-31G* Geometry
C -2.122484 2.356078 -0.095458
C -1.012353 0.140304 -0.096586
N -3.403191 0.412111 0.178658
C -2.298028 -0.492627 0.170336
C -3.330131 1.763956 0.085682
C -0.965742 1.524658 -0.178561
O 0.171262 2.238067 -0.313857
C 1.453244 1.573758 -0.414010
C 1.476172 0.227564 0.360885
C 0.310305 -0.640628 -0.241011
C 0.295007 -2.125800 0.249880
C 1.692170 -2.599205 0.721721
C 2.883974 -1.985778 -0.027468
C 2.873810 -0.432603 0.050742
O -2.539332 -1.684181 0.365788
C 2.479022 2.637422 -0.011818
C 2.480576 3.847292 -0.953869
C 1.270022 0.464361 1.872129
H 0.518579 -0.690665 -1.322461
C 3.049795 -2.472121 -1.478577
C -0.255170 -3.065223 -0.841157
O 3.899312 0.039073 0.933490
O -4.647554 -0.099473 0.525259
C -5.233901 -0.830879 -0.566041
H -2.016753 3.430399 -0.168587
H -4.273349 2.288308 0.175358
H 1.593840 1.337665 -1.480038
H -0.384769 -2.202200 1.100819
H 1.814446 -2.373112 1.789398
H 1.739992 -3.693491 0.651075
H 3.797045 -2.293823 0.499244
H 3.200095 -0.054270 -0.925187
H 2.257183 2.966231 1.010350
H 3.470662 2.178332 0.018164
H 2.728516 3.550737 -1.980937
H 1.503395 4.338881 -0.973154
H 3.225479 4.583774 -0.633092
H 0.357608 1.035915 2.057889
H 2.102966 1.026935 2.300644
H 1.171801 -0.476922 2.421050
H 3.025017 -3.566361 -1.534295
H 2.268252 -2.088693 -2.142541
H 4.013551 -2.136864 -1.880757
H -0.329214 -4.087900 -0.451765
H 0.394870 -3.093820 -1.722574
H -1.255684 -2.762041 -1.149767
H 3.747967 -0.363455 1.803066
H -4.622825 -1.701410 -0.810794
H -5.364947 -0.182670 -1.441260
H -6.208615 -1.145312 -0.186819
SCF Energy = -1057.74647723
Number of imaginary frequencies = 0

Compound 1_c31
B3LYP/6-31G* Geometry
C -2.419315 1.833272 -1.183661
C -1.057376 -0.115229 -0.527526
N -3.403482 -0.002573 -0.076146
C -2.197847 -0.750759 0.095734
C -3.523935 1.204242 -0.692311
C -1.175632 1.157186 -1.050958
O -0.060407 1.784228 -1.498102
C 1.126510 1.752410 -0.621281
C 1.299478 0.367525 0.120644
C 0.357289 -0.664198 -0.608419
C 0.590321 -2.145984 -0.229191
C 2.087711 -2.489658 -0.336100
C 3.036626 -1.535510 0.400703
C 2.776188 -0.095812 -0.070127
O -2.235022 -1.803890 0.733174
C 1.043533 3.053176 0.202147
C 2.349704 3.590738 0.797945
C 0.935132 0.449759 1.616868
H 0.634270 -0.589360 -1.674086
C 4.502180 -1.940287 0.188277
C -0.193693 -3.094540 -1.151850
O 3.707658 0.829130 0.497818
O -4.590894 -0.595008 0.333774
C -4.742399 -0.531874 1.764281
H -2.475566 2.808988 -1.648400
H -4.532564 1.595853 -0.739549
H 1.943556 1.855439 -1.340908
H 0.249528 -2.318614 0.796977
H 2.244921 -3.514496 0.025114
H 2.372685 -2.499262 -1.400602
H 2.822672 -1.591477 1.480003
H 2.994079 -0.057611 -1.146611
H 0.656079 3.803839 -0.496489
H 0.282718 2.949350 0.985309
H 2.737545 2.969232 1.607351
H 3.136368 3.654698 0.039206
H 2.180674 4.596887 1.198716
H -0.089704 0.803420 1.757058
H 1.591303 1.134829 2.163028

H 0.998844 -0.530333 2.097649
H 4.678369 -2.957961 0.555480
H 4.760407 -1.920675 -0.878278
H 5.187142 -1.262366 0.705114
H 0.062454 -4.136749 -0.925057
H 0.062839 -2.913974 -2.204586
H -1.270734 -2.984845 -1.023437
H 3.674457 0.722645 1.461747
H -4.752694 0.510469 2.106574
H -3.949028 -1.098399 2.254380
H -5.713775 -0.993942 1.952960
SCF Energy = -1057.75275738
Number of imaginary frequencies = 0

Compound 1_c32
B3LYP/6-31G* Geometry
C -2.077677 2.334679 0.546075
C -0.952852 0.191345 0.067424
N -3.339519 0.473503 -0.154939
C -2.209312 -0.347022 -0.436289
C -3.281296 1.762990 0.280259
C -0.904459 1.542922 0.363279
O 0.263016 2.221873 0.457704
C 1.366574 1.609177 -0.262261
C 1.600687 0.196732 0.324379
C 0.315673 -0.657164 -0.002375
C 0.356467 -1.947178 0.884170
C 1.773161 -2.612226 0.780159
C 2.715604 -2.077453 -0.323210
C 2.783232 -0.516390 -0.396630
O -2.365397 -1.398386 -1.052706
C 2.506180 2.626972 -0.224643
C 2.172261 3.909238 -0.996498
C 1.869827 0.353754 1.841398
H 0.400063 -0.985463 -1.047274
C 2.432499 -2.715256 -1.696981
C -0.722874 -3.013340 0.640947
O 4.064448 -0.036821 0.027790
O -4.570589 0.080602 -0.671753
C -5.125611 -1.014382 0.076169
H -1.992351 3.371979 0.842988
H -4.236939 2.266673 0.354498
H 1.035513 1.490028 -1.306534
H 0.222282 -1.610286 1.919407
H 2.281855 -2.528172 1.748349
H 1.650565 -3.689962 0.619611
H 3.734116 -2.388492 -0.056526
H 2.745188 -0.226037 -1.454015
H 2.730223 2.877135 0.818714
H 3.406113 2.156818 -0.629970
H 3.003160 4.621114 -0.941763
H 1.985675 3.697876 -2.056857
H 1.280570 4.395547 -0.589250
H 0.990776 0.743619 2.360229
H 2.685024 1.062643 2.010369
H 2.147418 -0.584257 2.325723
H 2.526376 -3.806022 -1.638810
H 1.425736 -2.494445 -2.067601
H 3.149740 -2.358256 -2.445771
H -0.513590 -3.874292 1.289512
H -0.733312 -3.359727 -0.396386
H -1.726582 -2.657055 0.872889
H 4.189554 -0.308867 0.950533
H -5.250376 -0.741246 1.130967
H -4.498426 -1.902121 -0.026951
H -6.102083 -1.185790 -0.382027
SCF Energy = -1057.74249907
Number of imaginary frequencies = 0

Compound 1_c33
B3LYP/6-31G* Geometry
C -2.176365 2.306023 -0.301558
C -1.012202 0.118310 -0.283434
N -3.423101 0.325276 -0.183037
C -2.300904 -0.557105 -0.200061
C -3.379373 1.679501 -0.252489
C -0.995310 1.505332 -0.306313
O 0.130642 2.248557 -0.307360
C 1.431225 1.614444 -0.349663
C 1.427393 0.242035 0.381653
C 0.335766 -0.628545 -0.344396
C 0.309710 -2.132224 0.090201
C 1.650472 -2.585736 0.716794
C 2.914151 -1.941937 0.125765
C 2.852772 -0.386095 0.156827
O -2.528424 -1.764691 -0.119024
C 2.404050 2.686909 0.148379
C 2.425329 3.930299 -0.748965
C 1.100798 0.430612 1.878609
H 0.638068 -0.636274 -1.404816
C 3.300043 -2.451172 -1.275090
C -0.074982 -3.042895 -1.091578
O 3.826418 0.138668 1.068380
O -4.687759 -0.244597 -0.248878
C -5.089829 -0.776802 1.026568
H -2.093207 3.384017 -0.343030
H -4.341377 2.176567 -0.267651
H 1.643753 1.417167 -1.411586
H -0.465454 -2.259306 0.848661
H 1.640166 -2.376328 1.794127
H 1.728528 -3.677763 0.636608
H 3.751717 -2.212081 0.783135
H 3.207566 -0.023677 -0.815450
H 2.118318 2.972365 1.167879
H 3.403247 2.250361 0.216871
H 3.131426 4.672560 -0.360700
H 2.738542 3.677598 -1.769893
H 1.438195 4.398172 -0.807207
H 0.191843 1.022579 2.009932
H 1.907602 0.958294 2.394265
H 0.936058 -0.524644 2.384471
H 3.321416 -3.546485 -1.306591
H 2.605579 -2.110353 -2.049935
H 4.297995 -2.086430 -1.546826
H -0.157519 -4.082731 -0.752542
H 0.675781 -3.014154 -1.888555
H -1.041937 -2.757252 -1.505780
H 3.658957 -0.258196 1.937415
H -5.117041 0.015011 1.785521
H -4.422067 -1.585693 1.327314
H -6.097929 -1.159481 0.853152
SCF Energy = -1057.74638414
Number of imaginary frequencies = 0

Compound 1_c34
B3LYP/6-31G* Geometry
C -2.290947 2.302602 -0.180655
C -1.086926 0.148780 -0.311381
N -3.488846 0.282828 -0.100699
C -2.355523 -0.561439 -0.287686
C -3.473091 1.641387 -0.080916
C -1.096114 1.534754 -0.314753
O 0.016063 2.292727 -0.432373
C 1.275296 1.641353 -0.742649
C 1.400582 0.340937 0.110590
C 0.252952 -0.587613 -0.434205
C 0.290874 -2.063101 0.107081
C 1.534299 -2.379223 0.971519
C 2.877680 -1.757213 0.521099
C 2.722839 -0.393821 -0.206544
O -2.542294 -1.775426 -0.384478
C 2.316469 2.768598 -0.698420
C 2.562733 3.466882 0.642097
C 1.256139 0.655279 1.621872
H 0.460850 -0.679660 -1.511353
C 3.740423 -2.722887 -0.310154
C 0.157486 -3.050532 -1.066259
O 3.827774 0.454701 0.130468
O -4.747469 -0.302797 -0.165877
C -5.051704 -1.018661 1.044509
H -2.239358 3.383498 -0.179420
H -4.443685 2.114072 0.003576
H 1.213274 1.325278 -1.794314
H -0.581077 -2.222493 0.743741

H 1.341411 -2.056512 1.998990
H 1.643463 -3.470045 1.028208
H 3.440387 -1.524246 1.434820
H 2.733419 -0.572934 -1.296453
H 3.256988 2.368987 -1.083138
H 1.959626 3.512793 -1.423412
H 3.206620 4.340598 0.487911
H 1.627454 3.819417 1.088916
H 3.065284 2.806612 1.352635
H 0.716500 1.590764 1.781838
H 2.238586 0.755355 2.090092
H 0.702269 -0.124989 2.147581
H 3.976575 -3.627192 0.261849
H 3.227946 -3.032463 -1.227444
H 4.696555 -2.268206 -0.606057
H 0.144483 -4.084941 -0.701884
H 0.992477 -2.956578 -1.773491
H -0.776182 -2.873853 -1.604448
H 4.637855 -0.066328 0.017353
H -5.036417 -0.343298 1.908809
H -4.353042 -1.846001 1.180774
H -6.063726 -1.397571 0.886445
SCF Energy = -1057.74450264
Number of imaginary frequencies = 0

Compound 1_c35
B3LYP/6-31G* Geometry
C 2.208042 2.116719 -0.787894
C 0.954993 0.069448 -0.211099
N 3.366031 0.159657 -0.191177
C 2.203094 -0.585066 0.152313
C 3.379146 1.441398 -0.647552
C 0.995322 1.425381 -0.490035
O -0.112000 2.202932 -0.467269
C -1.209286 1.657005 0.310120
C -1.595561 0.283310 -0.293265
C -0.368906 -0.677997 -0.058088
C -0.573996 -1.950812 -0.951603
C -2.040679 -2.480927 -0.783779
C -2.850124 -1.901533 0.397001
C -2.783342 -0.342353 0.483840
O 2.329740 -1.658294 0.739102
C -2.266444 2.758542 0.372063
C -1.784231 3.994740 1.140236
C -1.934792 0.499123 -1.788624
H -0.407359 -1.011078 0.988296
C -2.515605 -2.586555 1.735897
C 0.407314 -3.119500 -0.766883
O -3.993357 0.252520 -0.001755
O 4.599279 -0.464734 -0.061509
C 5.034989 -0.492109 1.311328
H 2.180229 3.155869 -1.089215
H 4.360703 1.848258 -0.857177
H -0.820695 1.483899 1.327210
H -0.460308 -1.615464 -1.989481
H -2.602511 -2.298968 -1.706076
H -2.015568 -3.571345 -0.670767
H -3.904694 -2.133793 0.190337
H -2.655360 -0.071773 1.546984
H -2.547877 3.044016 -0.647344
H -3.167722 2.346733 0.834397
H -2.563344 4.764676 1.163506
H -1.532404 3.745379 2.178924
H -0.893548 4.428141 0.674884
H -1.053038 0.829590 -2.343577
H -2.706807 1.263404 -1.890843
H -2.321624 -0.398561 -2.269057
H -2.710605 -3.663357 1.671860
H -1.467039 -2.463695 2.025951
H -3.132332 -2.185163 2.550391
H 0.081675 -3.949089 -1.408229
H 0.429690 -3.476904 0.267172
H 1.430242 -2.864765 -1.040947
H -4.724128 -0.109537 0.522915
H 4.345013 -1.083102 1.915360
H 5.131882 0.526637 1.707076
H 6.016428 -0.969876 1.273275
SCF Energy = -1057.74172816
Number of imaginary frequencies = 0

Compound 1_c36
B3LYP/6-31G* Geometry
C -2.289668 2.282878 0.056756
C -1.091347 0.128304 -0.116628
N -3.473858 0.264483 0.255258
C -2.346048 -0.584649 0.062445
C -3.456905 1.622645 0.271920
C -1.108362 1.513545 -0.162623
O -0.015379 2.270263 -0.403680
C 1.217150 1.611255 -0.794974
C 1.423146 0.345047 0.092566
C 0.239873 -0.607845 -0.316622
C 0.332062 -2.059569 0.279660
C 1.651457 -2.342000 1.036577
C 2.945175 -1.731279 0.447756
C 2.717419 -0.397162 -0.312110
O -2.531181 -1.802882 0.045598
C 2.250790 2.742563 -0.884146
C 2.609688 3.491100 0.402703
C 1.405498 0.713509 1.598129
H 0.358875 -0.744442 -1.402905
C 3.739182 -2.722078 -0.422282
C 0.093952 -3.094260 -0.835261
O 3.842241 0.466957 -0.107355
O -4.685767 -0.333706 0.574520
C -5.307816 -0.910801 -0.588499
H -2.238937 3.363717 0.042152
H -4.412247 2.095665 0.462463
H 1.066955 1.254126 -1.824446
H -0.476375 -2.191355 1.000506
H 1.549833 -1.984952 2.065609
H 1.772305 -3.429755 1.119892
H 3.584416 -1.463488 1.299733
H 2.631849 -0.617654 -1.391249
H 3.156554 2.331447 -1.334472
H 1.827298 3.458041 -1.602227
H 3.233928 4.359122 0.160275
H 1.716007 3.859926 0.916660
H 3.175758 2.858936 1.090751
H 0.871614 1.650249 1.769974
H 2.423061 0.837733 1.977242
H 0.906198 -0.050869 2.196836
H 4.022002 -3.609548 0.154845
H 3.155315 -3.057895 -1.286643
H 4.667840 -2.277361 -0.807735
H 0.865113 -3.034814 -1.614834
H -0.882536 -2.934690 -1.297619
H 0.109310 -4.112393 -0.428019
H 4.642633 -0.058095 -0.262732
H -4.684954 -1.709943 -0.993471
H -5.503400 -0.141095 -1.345462
H -6.252197 -1.314274 -0.216810
SCF Energy = -1057.74435915
Number of imaginary frequencies = 0

Compound 1_c37
B3LYP/6-31G* Geometry
C 2.211493 2.112649 -0.795037
C 0.956287 0.068242 -0.214227
N 3.367051 0.157142 -0.186803
C 2.202798 -0.586641 0.154346
C 3.381878 1.437251 -0.647414
C 0.997691 1.422651 -0.499309
O -0.111186 2.199191 -0.484414
C -1.206361 1.659885 0.303080
C -1.596383 0.283180 -0.287974
C -0.368107 -0.677807 -0.061500
C -0.574435 -1.947283 -0.958184
C -2.036527 -2.486656 -0.778109
C -2.855400 -1.902140 0.396213
C -2.783053 -0.343571 0.502759
O 2.326346 -1.659313 0.742363
C -2.259899 2.765390 0.365282
C -1.771783 3.998796 1.134512

C -1.951031 0.494453 -1.780560
H -0.406502 -1.015551 0.983545
C -2.537201 -2.594183 1.735512
C 0.415023 -3.111162 -0.788599
O -4.044455 0.253964 0.181275
O 4.599585 -0.467553 -0.052732
C 5.031155 -0.494057 1.321668
H 2.185596 3.151096 -1.098943
H 4.364359 1.843464 -0.853958
H -0.813006 1.491205 1.318764
H -0.470463 -1.607248 -1.995958
H -2.596055 -2.331306 -1.708548
H -2.001034 -3.574887 -0.649701
H -3.912284 -2.119477 0.193646
H -2.644975 -0.083330 1.559698
H -2.535839 3.056443 -0.654794
H -3.163984 2.358237 0.825676
H -2.546717 4.772839 1.156014
H -1.525523 3.747147 2.173819
H -0.877056 4.427214 0.672168
H -1.078699 0.822383 -2.350759
H -2.712724 1.272510 -1.881919
H -2.337286 -0.405742 -2.262167
H -2.723572 -3.672103 1.662736
H -1.493559 -2.464290 2.041874
H -3.172444 -2.198928 2.537322
H 0.089411 -3.938444 -1.432898
H 0.447234 -3.474187 0.243004
H 1.434228 -2.848448 -1.069552
H -4.256497 0.018781 -0.735723
H 4.339257 -1.084498 1.924005
H 5.127075 0.524949 1.716847
H 6.012530 -0.972106 1.286717
SCF Energy = -1057.74228352
Number of imaginary frequencies = 0

Compound 1_c38
B3LYP/6-31G* Geometry
C -2.167402 2.311516 0.059035
C -1.015511 0.120755 -0.084038
N -3.413809 0.332396 0.200536
C -2.295547 -0.551210 0.114462
C -3.364043 1.688105 0.206336
C -0.999473 1.506858 -0.090337
O 0.125282 2.250418 -0.210518
C 1.381667 1.592549 -0.460950
C 1.475353 0.253501 0.328152
C 0.317244 -0.640732 -0.249463
C 0.337921 -2.118177 0.270428
C 1.725801 -2.516508 0.825695
C 2.937556 -1.938259 0.079847
C 2.871861 -0.391359 -0.009325
O -2.519011 -1.758305 0.197629
C 2.449546 2.660955 -0.195742
C 2.197091 3.976023 -0.944937
C 1.292846 0.503700 1.846268
H 0.510017 -0.707994 -1.332983
C 3.218326 -2.566160 -1.296582
C -0.120283 -3.100383 -0.824912
O 3.925639 0.076394 0.840728
O -4.650038 -0.225722 0.499803
C -5.226140 -0.869211 -0.651366
H -2.081172 3.390048 0.053882
H -4.315229 2.188622 0.337908
H 1.406031 1.350308 -1.534511
H -0.379245 -2.207859 1.089483
H 1.806039 -2.198843 1.871669
H 1.798868 -3.611784 0.842676
H 3.817247 -2.142583 0.700727
H 3.104590 -0.104941 -1.049285
H 2.514131 2.858230 0.882009
H 3.417859 2.264709 -0.526827
H 3.013519 4.682959 -0.762458
H 2.134393 3.807941 -2.026814
H 1.262137 4.439285 -0.621556
H 0.703645 1.405950 2.033390
H 2.260785 0.617713 2.339213
H 0.770338 -0.325387 2.329032
H 3.236599 -3.660320 -1.235270
H 2.471447 -2.288594 -2.047546
H 4.195346 -2.234979 -1.669778
H -0.158230 -4.119530 -0.421307
H 0.564232 -3.110230 -1.679861
H -1.121126 -2.850324 -1.176978
H 3.974044 1.041907 0.783090
H -4.602085 -1.705811 -0.969990
H -5.367725 -0.149633 -1.467188
H -6.195653 -1.229692 -0.301036
SCF Energy = -1057.74445846
Number of imaginary frequencies = 0

Compound 1_c39
B3LYP/6-31G* Geometry
C -2.297446 2.298783 -0.181066
C -1.090051 0.146805 -0.306554
N -3.492518 0.276901 -0.099637
C -2.357497 -0.565941 -0.284112
C -3.478765 1.635441 -0.082650
C -1.101320 1.532663 -0.312057
O 0.011012 2.292444 -0.427743
C 1.272090 1.643103 -0.738267
C 1.400187 0.342007 0.110333
C 0.251078 -0.586543 -0.431078
C 0.295065 -2.065021 0.102516
C 1.538877 -2.383779 0.966533
C 2.882025 -1.752224 0.525342
C 2.725104 -0.400068 -0.217246
O -2.541298 -1.780143 -0.380055
C 2.312182 2.771209 -0.689989
C 2.538334 3.483544 0.647287
C 1.252386 0.654193 1.621225
H 0.457909 -0.673428 -1.508777
C 3.778230 -2.702576 -0.283683
C 0.169819 -3.045146 -1.077745
O 3.905746 0.385680 -0.023445
O -4.750102 -0.310439 -0.168152
C -5.056756 -1.027704 1.040872
H -2.248303 3.379793 -0.182541
H -4.450273 2.106839 -0.001546
H 1.212798 1.330032 -1.790682
H -0.577910 -2.232098 0.735678
H 1.339575 -2.076981 1.998325
H 1.653405 -3.474448 1.010361
H 3.431667 -1.516705 1.450360
H 2.720634 -0.588386 -1.299228
H 3.257775 2.371065 -1.060629
H 1.962898 3.512236 -1.421471
H 3.177047 4.360748 0.493861
H 1.596087 3.829995 1.083476
H 3.039015 2.840285 1.376679
H 0.689711 1.575280 1.784910
H 2.226784 0.788244 2.104519
H 0.728311 -0.141669 2.152867
H 4.036452 -3.592610 0.301687
H 3.277366 -3.040721 -1.198020
H 4.708051 -2.199961 -0.571511
H 0.151987 -4.081433 -0.719260
H 1.012084 -2.948668 -1.775870
H -0.758738 -2.864245 -1.623580
H 4.069365 0.440723 0.931397
H -5.044561 -0.353014 1.905737
H -4.357695 -1.854507 1.178266
H -6.067926 -1.407470 0.879633
SCF Energy = -1057.74483330
Number of imaginary frequencies = 0

Compound 1_c40
B3LYP/6-31G* Geometry
C -2.294551 2.367629 0.097112
C -1.118140 0.219388 -0.220685
N -3.524597 0.377074 -0.121049
C -2.395469 -0.462624 -0.349522
C -3.489774 1.723826 0.057863
C -1.105877 1.594967 -0.064255

O   0.021371 2.337670 -0.032444
C   1.304412 1.720302 -0.339136
C   1.357690 0.311469 0.325581
C   0.216514 -0.515295 -0.373345
C   0.207115 -2.049897 -0.028843
C   1.391062 -2.501870 0.857188
C   2.766797 -1.855460 0.567616
C   2.669854 -0.427051 -0.030240
O -2.594352 -1.652031 -0.602755
C   2.339948 2.794177 0.056803
C   3.391561 3.065568 -1.024473
C   1.184714 0.472110 1.856556
H   0.470632 -0.469931 -1.444149
C   3.677297 -2.745672 -0.296966
C   0.131685 -2.872476 -1.327624
O   3.789616 0.347710 0.414544
O -4.786272 -0.169049 -0.324694
C -5.168746 -1.019686 0.770672
H -2.227417 3.439738 0.227607
H -4.456443 2.203710 0.146596
H   1.330621 1.586542 -1.430246
H -0.703517 -2.272918 0.529074
H   1.140008 -2.310759 1.904837
H   1.480651 -3.593010 0.778382
H   3.268476 -1.720633 1.535096
H   2.705004 -0.501404 -1.131232
H   1.773209 3.713797 0.239663
H   2.831570 2.527165 0.994361
H   4.073039 3.862025 -0.704353
H   3.990984 2.173419 -1.222544
H   2.922203 3.388599 -1.962361
H   0.593217 1.360128 2.093438
H   2.160126 0.584342 2.338459
H   0.676734 -0.381767 2.308322
H   3.860009 -3.708720 0.192704
H   3.230339 -2.948559 -1.276636
H   4.659063 -2.283653 -0.474192
H   0.079669 -3.945014 -1.104648
H   1.010500 -2.706075 -1.964704
H -0.763961 -2.605174 -1.892921
H   4.587365 -0.180829 0.257636
H -5.187197 -0.455828 1.711429
H -4.494351 -1.875110 0.839313
H -6.177821 -1.350424 0.515396
SCF Energy = -1057.74451052
Number of imaginary frequencies = 0

Compound 1_c41
B3LYP/6-31G* Geometry
C -2.296613 2.278487 0.053644
C -1.094193 0.125881 -0.113289
N -3.477219 0.258033 0.256172
C -2.347730 -0.589559 0.065850
C -3.462870 1.616181 0.268830
C -1.113728 1.510860 -0.162358
O -0.020572 2.269538 -0.401830
C   1.214876 1.613291 -0.791166
C   1.422660 0.346116 0.091593
C   0.238511 -0.606816 -0.314705
C   0.336147 -2.061939 0.273276
C   1.655755 -2.348430 1.030070
C   2.950633 -1.724482 0.454478
C   2.718852 -0.404074 -0.322404
O -2.529814 -1.807998 0.051213
C   2.246833 2.747074 -0.873613
C   2.578376 3.510376 0.412650
C   1.399871 0.711966 1.597121
H   0.356858 -0.737952 -1.401642
C   3.784690 -2.698337 -0.392766
C   0.104334 -3.089340 -0.849621
O   3.906618 0.392606 -0.267291
O -4.688487 -0.341613 0.574734
C -5.307922 -0.922408 -0.588126
H -2.248577 3.359405 0.035644
H -4.419458 2.087867 0.456430
H   1.069797 1.259283 -1.822129
H -0.473493 -2.201363 0.991210
H   1.546504 -2.011933 2.065925
H   1.781496 -3.436636 1.095784
H   3.573982 -1.450975 1.320585
H   2.618365 -0.635972 -1.391278
H   3.161030 2.336604 -1.306404
H   1.832151 3.458252 -1.600596
H   3.197803 4.382559 0.174504
H   1.673664 3.871389 0.912008
H   3.140689 2.896645 1.122380
H   0.832925 1.627653 1.775936
H   2.408922 0.882496 1.989242
H   0.940973 -0.073142 2.200178
H   4.091963 -3.569100 0.197805
H   3.215074 -3.065936 -1.254338
H   4.686762 -2.203598 -0.768985
H   0.114896 -4.109652 -0.448012
H   0.882363 -3.026956 -1.622096
H -0.868374 -2.925921 -1.318913
H   4.150274 0.493976 0.666304
H -4.683258 -1.721531 -0.990301
H -5.503451 -0.154641 -1.347016
H -6.252197 -1.326399 -0.216829
SCF Energy = -1057.74477292
Number of imaginary frequencies = 0

Compound 1_c42
B3LYP/6-31G* Geometry
C -2.073691 2.328644 0.530601
C -0.950074 0.184470 0.039992
N -3.343647 0.461435 -0.134565
C -2.216561 -0.356101 -0.439236
C -3.281127 1.752733 0.293310
C -0.903229 1.538057 0.329442
O   0.263372 2.227113 0.400220
C   1.355887 1.595113 -0.306038
C   1.590780 0.198222 0.318881
C   0.321774 -0.664187 -0.037122
C   0.365402 -1.966259 0.835328
C   1.800743 -2.596607 0.774455
C   2.749565 -2.054896 -0.314458
C   2.822455 -0.503046 -0.333875
O -2.383931 -1.405598 -1.054570
C   2.504442 2.606687 -0.311473
C   2.120818 3.944952 -0.955819
C   1.780383 0.373644 1.846599
H   0.417351 -0.975767 -1.085730
C   2.474619 -2.660343 -1.703743
C -0.676812 -3.055394 0.535550
O   4.057573 -0.167209 0.308889
O -4.584050 0.062349 -0.623284
C -5.118548 -1.032441 0.140017
H -1.984677 3.367116 0.822160
H -4.236504 2.253766 0.386088
H   1.025415 1.453438 -1.347741
H   0.187562 -1.648026 1.869681
H   2.292241 -2.476510 1.746170
H   1.707708 -3.679684 0.630419
H   3.763529 -2.368201 -0.039686
H   2.866472 -0.180294 -1.388872
H   2.855707 2.783624 0.712238
H   3.337762 2.173137 -0.879982
H   2.977141 4.627689 -0.967194
H   1.790910 3.803509 -1.991955
H   1.306848 4.424271 -0.405802
H   0.845393 0.671968 2.327194
H   2.520500 1.147163 2.066887
H   2.137071 -0.537345 2.324995
H   2.565491 -3.752188 -1.664316
H   1.473212 -2.432651 -2.083991
H   3.201092 -2.293060 -2.438979
H -0.461875 -3.925380 1.169672
H -0.644383 -3.378703 -0.509333
H -1.697191 -2.734194 0.741556
H   4.149910 0.797067 0.317828
H -6.104435 -1.208243 -0.295726
H -5.220313 -0.756734 1.196561
H -4.491074 -1.918539 0.025304

SCF Energy = -1057.74076409
Number of imaginary frequencies = 0

Compound 3_c01
B3LYP/6-31G* Geometry
C 1.683482 1.289677 -0.071068
C -0.809279 1.253333 -0.184687
N 0.454583 3.285225 -0.460677
C -0.798438 2.654938 -0.582614
C 1.633042 2.646453 -0.220962
C 0.411463 0.600538 -0.134010
O 0.502100 -0.751752 -0.185052
C -0.658858 -1.422541 -0.736532
C -1.880286 -1.043624 0.128860
C -2.131241 0.488719 -0.132510
C -3.262622 0.961561 0.845821
C -4.544058 0.192710 0.442982
C -4.419208 -1.340886 0.430962
C -3.149653 -1.806826 -0.308915
O -1.741359 3.307123 -1.030985
C 2.980882 0.593254 0.107016
C 5.496669 -0.634422 0.488875
C 4.107337 0.980875 -0.640934
C 3.150060 -0.438060 1.045311
C 4.387796 -1.047354 1.235033
C 5.351258 0.386859 -0.454311
O 6.736736 -1.193746 0.632021
C -0.281261 -2.897794 -0.864938
C 0.929404 -3.125817 -1.778569
C -1.543821 -1.364432 1.607580
H -2.568924 0.572361 -1.140205
C -5.673756 -1.999099 -0.158381
C -3.592244 2.457215 0.942364
H 0.442634 4.268731 -0.702709
H 2.518048 3.270276 -0.166139
H -0.811276 -1.015881 -1.749194
H -2.975294 0.655130 1.858825
H -5.364052 0.477596 1.116088
H -4.841464 0.537671 -0.560069
H -4.339493 -1.676190 1.474760
H -3.022583 -2.885411 -0.147072
H -3.292626 -1.668555 -1.392047
H 4.001501 1.750448 -1.400979
H 2.303321 -0.768197 1.637058
H 4.493524 -1.842589 1.971387
H 6.212958 0.689639 -1.040868
H 6.691631 -1.889013 1.306630
H -0.077390 -3.317974 0.126535
H -1.148068 -3.435767 -1.265610
H 1.153648 -4.194854 -1.863213
H 0.739545 -2.744132 -2.789393
H 1.816541 -2.618531 -1.389698
H -0.771246 -0.700049 2.004041
H -1.178521 -2.392277 1.701461
H -2.413936 -1.279582 2.259612
H -6.572429 -1.707440 0.398254
H -5.819888 -1.701470 -1.204711
H -5.602534 -3.093381 -0.129212
H -4.414135 2.584460 1.659821
H -3.896448 2.878510 -0.017305
H -2.744944 3.045253 1.302575
SCF Energy = -1174.35285696
Number of imaginary frequencies = 0

Compound 3_c02
B3LYP/6-31G* Geometry
C 1.659773 1.290842 0.033938
C -0.828502 1.262086 -0.155557
N 0.373112 3.236108 0.483363
C -0.890956 2.633999 0.312498
C 1.576023 2.617680 0.351854
C 0.397074 0.619446 -0.179288
O 0.494979 -0.709598 -0.407307
C -0.621437 -1.622645 -0.158116
C -1.895658 -0.921578 0.378385
C -2.035204 0.384938 -0.483480
C -3.437542 1.047841 -0.467856
C -4.550549 -0.003912 -0.644049
C -4.459797 -1.198471 0.311077
C -3.093000 -1.870852 0.125310
O -1.886739 3.316878 0.571128
C 2.978287 0.617246 -0.054542
C 5.541709 -0.554647 -0.243716
C 3.307619 -0.238271 -1.118698
C 3.968041 0.859140 0.914527
C 5.235131 0.291049 0.825911
C 4.569653 -0.819710 -1.214490
O 6.797506 -1.095194 -0.285267
C -0.054940 -2.757935 0.699615
C 1.075824 -3.531736 0.013208
C -1.799039 -0.598783 1.886203
H -1.894273 0.050593 -1.526707
C -5.603658 -2.194879 0.086531
C -3.575648 2.088712 -1.592005
H 0.320316 4.205933 0.770787
H 2.455127 3.232289 0.510146
H -0.864578 -2.024013 -1.150786
H -3.582346 1.562158 0.487270
H -5.522473 0.495834 -0.532585
H -4.522854 -0.387135 -1.677636
H -4.535684 -0.828007 1.343804
H -3.014087 -2.746795 0.783045
H -3.043739 -2.252533 -0.906545
H 2.568974 -0.448351 -1.884370
H 3.733304 1.490979 1.767209
H 5.990505 0.479989 1.582210
H 4.802405 -1.475194 -2.052304
H 6.869746 -1.662217 -1.068731
H 0.303050 -2.342571 1.648627
H -0.875219 -3.445414 0.941706
H 1.435139 -4.342970 0.656029
H 0.734439 -3.979680 -0.928644
H 1.921285 -2.874769 -0.209913
H -0.854154 -0.114515 2.146678
H -1.884138 -1.510259 2.487878
H -2.602408 0.077379 2.193563
H -6.579160 -1.714463 0.227286
H -5.577662 -2.600957 -0.933173
H -5.541599 -3.039704 0.783420
H -4.592922 2.499164 -1.602168
H -3.397511 1.626096 -2.572774
H -2.886465 2.922334 -1.458864
SCF Energy = -1174.35763811
Number of imaginary frequencies = 0

Compound 3_c03
B3LYP/6-31G* Geometry
C 1.674072 1.272881 0.151698
C -0.812229 1.272184 -0.051668
N 0.409571 3.224732 0.625251
C -0.858352 2.630227 0.459657
C 1.605739 2.593588 0.492704
C 0.407025 0.616285 -0.083856
O 0.511639 -0.703627 -0.355883
C -0.641871 -1.597797 -0.321740
C -1.905335 -0.943749 0.289807
C -2.033074 0.438926 -0.447141
C -3.428572 1.115208 -0.373357
C -4.558081 0.094443 -0.621069
C -4.470463 -1.179881 0.222937
C -3.115688 -1.849629 -0.041143
O -1.846278 3.306691 0.761349
C 2.993641 0.604263 0.015131
C 5.557192 -0.561298 -0.186053
C 4.005361 1.192159 -0.763347
C 3.301358 -0.587934 0.688939
C 4.563971 -1.167794 0.590982
C 5.273465 0.627187 -0.864099
O 6.814451 -1.083414 -0.319648
C -0.144194 -2.888261 0.335985
C 0.973900 -3.577125 -0.455407
C -1.788267 -0.771425 1.820108
H -1.901285 0.198746 -1.516741
C -5.631315 -2.138122 -0.070276

C -3.565159 2.232496 -1.423079
H 0.364203 4.177704 0.965505
H 2.491574 3.184865 0.696644
H -0.865313 -1.813136 -1.375677
H -3.562257 1.561971 0.616857
H -5.521152 0.595179 -0.452387
H -4.550721 -0.198056 -1.684134
H -4.525955 -0.900751 1.285147
H -3.045954 -2.783391 0.531959
H -3.079845 -2.134251 -1.104649
H 3.786690 2.102494 -1.315354
H 2.547332 -1.062964 1.306843
H 4.781302 -2.089958 1.127845
H 6.047139 1.085037 -1.472384
H 6.868958 -1.906013 0.191098
H 0.199713 -2.661582 1.352396
H -0.993795 -3.574417 0.435206
H 1.294380 -4.496269 0.047828
H 0.632760 -3.850853 -1.461443
H 1.844032 -2.923476 -0.562687
H -0.838457 -0.317459 2.115076
H -1.869127 -1.738028 2.329756
H -2.584799 -0.127271 2.204241
H -6.597739 -1.660587 0.130385
H -5.627913 -2.450828 -1.122724
H -5.569621 -3.043247 0.546394
H -4.577043 2.655153 -1.391750
H -3.407238 1.832584 -2.434412
H -2.864092 3.047485 -1.246417
SCF Energy = -1174.35642862
Number of imaginary frequencies = 0

Compound 3_c04
B3LYP/6-31G* Geometry
C 1.677266 1.270538 -0.222737
C -0.810897 1.223569 -0.328185
N 0.448094 3.243968 -0.702858
C -0.798793 2.599056 -0.810219
C 1.627825 2.617310 -0.434922
C 0.409149 0.571915 -0.251248
O 0.505620 -0.779873 -0.200443
C -0.678643 -1.498546 -0.629445
C -1.870224 -1.024299 0.232401
C -2.133600 0.471691 -0.182374
C -3.248118 1.042275 0.763914
C -4.533255 0.228399 0.477953
C -4.398609 -1.294283 0.641851
C -3.148839 -1.833924 -0.079227
O -1.736847 3.216681 -1.313848
C 2.978019 0.601057 0.041033
C 5.503992 -0.565039 0.510566
C 3.449491 -0.448956 -0.761380
C 3.806306 1.049812 1.083172
C 5.055786 0.482841 1.319140
C 4.694622 -1.029359 -0.532319
O 6.735398 -1.094610 0.783777
C -0.323777 -2.985082 -0.604020
C 0.847723 -3.350847 -1.522572
C -1.482189 -1.184689 1.724514
H -2.588980 0.450779 -1.185668
C -5.664469 -2.023065 0.172419
C -3.592914 2.536714 0.704817
H 0.438987 4.204964 -1.023176
H 2.518202 3.236183 -0.436559
H -0.869949 -1.205556 -1.674510
H -2.936103 0.849214 1.797235
H -5.340883 0.586753 1.130568
H -4.852951 0.453602 -0.551921
H -4.283496 -1.504607 1.714587
H -3.010749 -2.887480 0.196519
H -3.325915 -1.816735 -1.166138
H 2.840877 -0.806897 -1.584462
H 3.456376 1.849865 1.730278
H 5.688601 0.831785 2.129096
H 5.043011 -1.839016 -1.171765
H 6.915475 -1.811451 0.155911
H -0.093098 -3.296092 0.421023
H -1.212937 -3.545866 -0.913645
H 1.008369 -4.434674 -1.527404
H 0.655483 -3.038647 -2.556818
H 1.772832 -2.873250 -1.189530
H -0.680940 -0.500110 2.014172
H -1.133510 -2.203797 1.920559
H -2.324620 -1.007693 2.394171
H -6.548552 -1.672315 0.718188
H -5.844207 -1.850937 -0.896691
H -5.583393 -3.106026 0.327400
H -4.409157 2.729953 1.413879
H -3.913207 2.849970 -0.290543
H -2.750692 3.170194 0.990574
SCF Energy = -1174.35132451
Number of imaginary frequencies = 0

Compound 3_c05
B3LYP/6-31G* Geometry
C 1.682583 1.241011 0.036759
C -0.808093 1.248242 -0.132409
N 0.417460 3.154840 0.651450
C -0.853354 2.578564 0.445094
C 1.613574 2.538412 0.461455
C 0.412365 0.601194 -0.222828
O 0.500288 -0.705857 -0.557955
C -0.628795 -1.619754 -0.402156
C -1.883364 -0.965659 0.235014
C -2.028228 0.410145 -0.513955
C -3.425422 1.076808 -0.418142
C -4.549228 0.049063 -0.660712
C -4.449554 -1.222691 0.187935
C -3.090632 -1.883905 -0.077868
O -1.840725 3.246481 0.767218
C 2.993623 0.561782 -0.106563
C 5.545665 -0.616463 -0.387140
C 3.312177 -0.219168 -1.229838
C 3.988055 0.723356 0.875221
C 5.249699 0.152061 0.742008
C 4.568466 -0.802588 -1.370959
O 6.796371 -1.163783 -0.471738
C -0.047665 -2.909077 0.210131
C 0.804769 -2.776084 1.478661
C -1.781690 -0.756213 1.761714
H -1.907892 0.166015 -1.583974
C -5.603778 -2.190725 -0.099507
C -3.576698 2.207192 -1.450804
H 0.375823 4.099199 1.015335
H 2.499448 3.129880 0.664122
H -0.919078 -1.867137 -1.431048
H -3.550285 1.512455 0.578010
H -5.515339 0.543031 -0.489296
H -4.543531 -0.245803 -1.723213
H -4.505681 -0.940297 1.249102
H -3.006537 -2.812380 0.502676
H -3.062259 -2.177809 -1.138863
H 2.569067 -0.369576 -2.004975
H 3.761225 1.293917 1.772108
H 6.008394 0.278489 1.507925
H 4.792498 -1.400130 -2.253335
H 6.861431 -1.673006 -1.294537
H -0.876388 -3.608939 0.376484
H 0.577915 -3.364924 -0.567916
H 1.258461 -3.744611 1.717770
H 1.614585 -2.056610 1.327781
H 0.225751 -2.461409 2.350070
H -0.832361 -0.305319 2.059121
H -1.886494 -1.706324 2.296196
H -2.576991 -0.091835 2.113212
H -6.573371 -1.719865 0.101681
H -5.600327 -2.507235 -1.150823
H -5.533727 -3.093092 0.520266
H -4.588866 2.627562 -1.403241
H -3.427312 1.823483 -2.469671
H -2.874717 3.020643 -1.267973
SCF Energy = -1174.35293552
Number of imaginary frequencies = 0

Compound 3_c06
B3LYP/6-31G* Geometry
C 1.682585 1.243036 0.034535
C -0.808400 1.249827 -0.130152
N 0.418818 3.156434 0.652138
C -0.852413 2.579533 0.448810
C 1.614719 2.540252 0.459378
C 0.412050 0.603027 -0.223118
O 0.501277 -0.703615 -0.557755
C -0.628556 -1.617470 -0.409106
C -1.882013 -0.966038 0.232876
C -2.029273 0.412270 -0.511131
C -3.426561 1.078033 -0.409695
C -4.550610 0.050537 -0.652358
C -4.448022 -1.224097 0.191552
C -3.089534 -1.883731 -0.080188
O -1.838931 3.246882 0.774694
C 2.992402 0.561895 -0.111353
C 5.538970 -0.624544 -0.402929
C 3.312601 -0.210662 -1.244038
C 3.981809 0.710941 0.872682
C 5.243584 0.133518 0.732631
C 4.563240 -0.797065 -1.391694
O 6.752165 -1.224374 -0.601377
C -0.047817 -2.911270 0.193874
C 0.803256 -2.787729 1.464270
C -1.776618 -0.761303 1.759989
H -1.911631 0.171874 -1.582243
C -5.602638 -2.191599 -0.096128
C -3.581277 2.211177 -1.438870
H 0.377411 4.100897 1.015701
H 2.501177 3.131784 0.659445
H -0.919298 -1.857653 -1.439563
H -3.549088 1.510815 0.588015
H -5.516417 0.543542 -0.476441
H -4.547918 -0.240664 -1.715860
H -4.501366 -0.945404 1.253865
H -3.003437 -2.814138 0.496915
H -3.063623 -2.173928 -1.142254
H 2.568797 -0.348901 -2.020909
H 3.757174 1.273111 1.775331
H 5.990458 0.260898 1.514587
H 4.805349 -1.387696 -2.269750
H 7.328828 -1.024833 0.152424
H -0.876359 -3.612754 0.353934
H 0.578944 -3.360540 -0.586904
H 1.257893 -3.757715 1.695507
H 1.612334 -2.066156 1.319518
H 0.223158 -2.480810 2.337799
H -0.825600 -0.313362 2.056530
H -1.882174 -1.712751 2.291938
H -2.569699 -0.096240 2.115236
H -6.571890 -1.721862 0.109346
H -5.601895 -2.504430 -1.148539
H -5.530500 -3.096111 0.520274
H -4.593424 2.631108 -1.387061
H -3.434976 1.830176 -2.459188
H -2.878955 3.024333 -1.256143
SCF Energy = -1174.35284817
Number of imaginary frequencies = 0

Compound 3_c07
B3LYP/6-31G* Geometry
C 1.804262 1.245937 -0.121681
C -0.689421 1.296400 -0.207483
N 0.642813 3.271479 -0.561755
C -0.633918 2.684225 -0.646722
C 1.800470 2.598069 -0.315126
C 0.507869 0.601114 -0.148138
O 0.551725 -0.754518 -0.159188
C -0.639921 -1.398647 -0.683001
C -1.830651 -0.946847 0.191246
C -2.037112 0.581983 -0.113654
C -3.131425 1.120220 0.873057
C -4.445041 0.377842 0.527825
C -4.368106 -1.158267 0.571237
C -3.133228 -1.685087 -0.185806
O -1.559804 3.357365 -1.099991
C 3.077935 0.508790 0.063306
C 5.551037 -0.799781 0.452317
C 4.206478 0.827995 -0.713044
C 3.222197 -0.495965 1.033727
C 4.438855 -1.144706 1.227527
C 5.429951 0.193673 -0.523374
O 6.771340 -1.400836 0.597425
C -0.309937 -2.896163 -0.774308
C -0.907917 -3.591211 -2.004655
C -1.463517 -1.220597 1.673665
H -2.490415 0.653123 -1.115417
C -5.656593 -1.798669 0.038178
C -3.414413 2.627502 0.922591
H 0.662927 4.247092 -0.833320
H 2.708045 3.190604 -0.291093
H -0.783160 -1.007235 -1.702109
H -2.828764 0.838999 1.889010
H -5.239893 0.713598 1.207650
H -4.755221 0.694055 -0.480808
H -4.271874 -1.457846 1.624368
H -3.029849 -2.761859 0.004678
H -3.305958 -1.573073 -1.267016
H 4.118212 1.574646 -1.497857
H 2.372201 -0.774202 1.647121
H 4.525666 -1.918189 1.989125
H 6.293417 0.443253 -1.131980
H 6.710211 -2.070686 1.296054
H 0.782162 -2.974196 -0.817362
H -0.620678 -3.419885 0.136968
H -0.603623 -4.643243 -2.035002
H -2.002102 -3.563634 -2.006769
H -0.560833 -3.118493 -2.931211
H -0.708151 -0.521304 2.041861
H -1.061595 -2.232331 1.790756
H -2.327367 -1.146084 2.335408
H -6.531488 -1.457925 0.604718
H -5.820434 -1.536872 -1.015099
H -5.617994 -2.892743 0.108204
H -4.219288 2.805140 1.648529
H -3.723072 3.023827 -0.046370
H -2.543970 3.202150 1.247173
SCF Energy = -1174.34959186
Number of imaginary frequencies = 0

Compound 3_c08
B3LYP/6-31G* Geometry
C 1.801146 1.206213 -0.276785
C -0.690870 1.264875 -0.340947
N 0.646678 3.212217 -0.812902
C -0.630959 2.623316 -0.864579
C 1.803812 2.544631 -0.545250
C 0.502290 0.565645 -0.258752
O 0.540762 -0.786311 -0.166261
C -0.672893 -1.464926 -0.584993
C -1.831180 -0.924571 0.281777
C -2.041109 0.571992 -0.155433
C -3.109603 1.203400 0.804938
C -4.432382 0.434906 0.567421
C -4.355465 -1.089739 0.756035
C -3.145872 -1.688595 0.013118
O -1.554589 3.268311 -1.360932
C 3.076197 0.494441 -0.002975
C 5.566151 -0.746345 0.479954
C 3.440113 -0.683482 -0.674860
C 3.992811 1.026863 0.920605
C 5.224525 0.425207 1.160710
C 4.666617 -1.298972 -0.438113
O 6.784140 -1.306736 0.752554
C -0.358354 -2.968683 -0.543562
C -0.977068 -3.764728 -1.700174
C -1.417514 -1.064424 1.770211
H -2.517991 0.555281 -1.148736
C -5.660442 -1.773719 0.328061
C -3.396656 2.708342 0.718182
H 0.672595 4.160638 -1.167867
H 2.719318 3.123451 -0.594337

H -0.851554 -1.170425 -1.631045
H -2.779579 1.019622 1.834473
H -5.208176 0.837017 1.233059
H -4.769864 0.653921 -0.458093
H -4.224261 -1.286859 1.829216
H -3.038944 -2.743978 0.297767
H -3.351627 -1.674782 -1.068106
H 2.760175 -1.119088 -1.397917
H 3.725482 1.922745 1.474917
H 5.923496 0.840944 1.879632
H 4.929414 -2.209102 -0.975311
H 6.884776 -2.111944 0.221549
H 0.732315 -3.065618 -0.585880
H -0.665219 -3.403989 0.413981
H -0.690730 -4.820269 -1.635406
H -2.070678 -3.718529 -1.696733
H -0.631615 -3.385447 -2.669518
H -0.628135 -0.359209 2.041656
H -1.040952 -2.072363 1.972511
H -2.254162 -0.898741 2.449887
H -6.516710 -1.377733 0.887063
H -5.857676 -1.612626 -0.739697
H -5.619982 -2.856304 0.500627
H -4.188385 2.951096 1.439685
H -3.726674 3.010762 -0.277450
H -2.523685 3.314031 0.969974
SCF Energy = -1174.34866110
Number of imaginary frequencies = 0

Compound 3_c09
B3LYP/6-31G* Geometry
C 1.765295 -1.241408 -0.075613
C -0.722382 -1.309961 0.098014
N 0.533722 -3.162991 -0.744672
C -0.749752 -2.625893 -0.509353
C 1.718657 -2.527222 -0.540745
C 0.482095 -0.642412 0.207593
O 0.503261 0.647613 0.615060
C -0.568401 1.579461 0.230324
C -1.858359 0.870609 -0.286877
C -1.937012 -0.487687 0.506973
C -3.316230 -1.194675 0.489464
C -4.454245 -0.196774 0.779089
C -4.431547 1.056773 -0.099720
C -3.077145 1.758910 0.074146
O -1.727013 -3.309547 -0.828602
C 3.059821 -0.535903 0.080659
C 5.577169 0.709883 0.379693
C 3.352378 0.244646 1.211030
C 4.061804 -0.662924 -0.898368
C 5.306569 -0.058319 -0.756196
C 4.591790 0.861617 1.360992
O 6.812095 1.290996 0.472750
C 0.128865 2.608954 -0.674533
C -0.629397 3.919047 -0.920975
C -1.825403 0.621875 -1.810314
H -1.769839 -0.205116 1.561749
C -5.590640 2.005305 0.231360
C -3.375383 -2.323566 1.531819
H 0.513066 -4.099774 -1.129633
H 2.614258 -3.094543 -0.768192
H -0.819176 2.066777 1.180128
H -3.478029 -1.641524 -0.496525
H -5.414591 -0.718970 0.670182
H -4.397167 0.119669 1.833821
H -4.541109 0.748784 -1.149658
H -3.058901 2.679084 -0.521148
H -2.995479 2.070685 1.126818
H 2.602822 0.368287 1.984541
H 3.853786 -1.230893 -1.801451
H 6.071219 -0.157844 -1.520179
H 4.796183 1.458142 2.248827
H 6.860840 1.793723 1.300657
H 1.080336 2.839756 -0.182100
H 0.389509 2.136852 -1.628940
H 0.026463 4.640314 -1.420951
H -1.509020 3.784011 -1.557527
H -0.963660 4.374203 0.019274
H -0.901904 0.131270 -2.129163
H -1.913858 1.561712 -2.366077
H -2.653382 -0.022816 -2.118861
H -6.558606 1.507386 0.099219
H -5.533561 2.348512 1.272726
H -5.579314 2.893148 -0.412832
H -4.376442 -2.772001 1.545276
H -3.175221 -1.933621 2.539389
H -2.663313 -3.118794 1.308912
SCF Energy = -1174.35362607
Number of imaginary frequencies = 0

Compound 3_c10
B3LYP/6-31G* Geometry
C -1.793797 -1.198027 0.202738
C 0.688486 -1.310121 0.006309
N -0.596531 -3.142593 0.859991
C 0.693239 -2.620805 0.627222
C -1.769965 -2.483500 0.666216
C -0.502715 -0.614859 -0.083476
O -0.532327 0.672014 -0.492396
C 0.620677 1.569999 -0.344722
C 1.885161 0.868653 0.229969
C 1.919256 -0.544087 -0.466868
C 3.280158 -1.288626 -0.424236
C 4.447154 -0.341845 -0.766106
C 4.461294 0.958334 0.039304
C 3.131508 1.691656 -0.183707
O 1.659386 -3.312107 0.964120
C -3.088355 -0.501277 -0.007338
C -5.608736 0.726286 -0.341891
C -4.124105 -1.137054 -0.713834
C -3.349306 0.771680 0.523034
C -4.590513 1.381485 0.359159
C -5.371210 -0.541716 -0.878977
O -6.846926 1.275399 -0.534476
C 0.033497 2.786145 0.393402
C 0.846253 4.086187 0.347869
C 1.832159 0.740118 1.767385
H 1.748385 -0.330134 -1.537181
C 5.652039 1.848943 -0.336959
C 3.303989 -2.461800 -1.418386
H -0.587266 -4.065468 1.277427
H -2.675308 -3.021628 0.924420
H 0.852434 1.870872 -1.374264
H 3.435366 -1.695432 0.579922
H 5.391033 -0.886142 -0.625827
H 4.401129 -0.083830 -1.837183
H 4.554900 0.708267 1.106251
H 3.145369 2.643283 0.358079
H 3.061832 1.943979 -1.253208
H -3.940794 -2.111728 -1.158456
H -2.576896 1.288056 1.081573
H -4.771603 2.367494 0.784616
H -6.163228 -1.037803 -1.431091
H -6.868651 2.152633 -0.121656
H -0.938594 2.968450 -0.079084
H -0.178760 2.511635 1.433925
H 0.230074 4.920457 0.701224
H 1.737283 4.051591 0.980982
H 1.168963 4.324479 -0.672652
H 0.878824 0.334542 2.117066
H 1.977666 1.714337 2.247764
H 2.617619 0.071467 2.131633
H 6.602707 1.329310 -0.168043
H 5.612532 2.132068 -1.397010
H 5.665178 2.772839 0.254368
H 4.292536 -2.937332 -1.414262
H 3.113164 -2.107543 -2.440866
H 2.571759 -3.227624 -1.163040
SCF Energy = -1174.35205988
Number of imaginary frequencies = 0

Compound 3_c11
B3LYP/6-31G* Geometry
C 1.728453 1.306698 -0.059905

C -0.765783 1.310715 -0.162904
N 0.524641 3.343779 -0.263799
C -0.737581 2.742512 -0.431676
C 1.695592 2.671669 -0.087729
C 0.448688 0.640795 -0.182254
O 0.526520 -0.701033 -0.369515
C -0.660435 -1.290894 -0.952864
C -1.849546 -0.989218 -0.015204
C -2.099829 0.559732 -0.162331
C -3.220637 0.964224 0.857646
C -4.505106 0.214745 0.426251
C -4.380082 -1.315144 0.318714
C -3.124686 -1.726852 -0.473780
O -1.674152 3.445431 -0.811519
C 3.016342 0.580029 0.055755
C 5.513383 -0.711373 0.328006
C 4.143032 1.002875 -0.671667
C 3.175142 -0.516977 0.917750
C 4.403715 -1.158000 1.053542
C 5.378338 0.376860 -0.538598
O 6.744983 -1.299537 0.420496
C -0.292746 -2.722634 -1.368007
C 0.203935 -3.709046 -0.303383
C -1.480486 -1.384270 1.437711
H -2.544016 0.722659 -1.157481
C -5.645006 -1.937525 -0.287114
C -3.559732 2.449004 1.046691
H 0.523432 4.345204 -0.415833
H 2.589101 3.276384 0.019294
H -0.849391 -0.748980 -1.892737
H -2.918683 0.599871 1.846850
H -5.315924 0.456783 1.126594
H -4.817758 0.618263 -0.550031
H -4.280058 -1.714345 1.337902
H -2.986888 -2.813506 -0.388390
H -3.291584 -1.518152 -1.542132
H 4.043818 1.827777 -1.372266
H 2.328167 -0.871161 1.495538
H 4.502420 -2.004222 1.731746
H 6.240977 0.707296 -1.108610
H 6.693445 -2.039345 1.045424
H -1.156564 -3.145057 -1.895879
H 0.495829 -2.611762 -2.123034
H 0.627411 -4.593399 -0.793045
H 0.989444 -3.262001 0.313556
H -0.596976 -4.058075 0.355446
H -0.720243 -0.719821 1.857285
H -1.089502 -2.401819 1.477774
H -2.343581 -1.348936 2.103977
H -6.533481 -1.682884 0.303045
H -5.810466 -1.575119 -1.309906
H -5.572599 -3.031368 -0.327887
H -4.380647 2.526304 1.772401
H -3.870849 2.925750 0.115227
H -2.715912 3.020776 1.438163
SCF Energy = -1174.34718840
Number of imaginary frequencies = 0

Compound 3_c12
B3LYP/6-31G* Geometry
C 1.727819 1.306983 -0.055695
C -0.766159 1.311351 -0.164298
N 0.525273 3.344230 -0.261547
C -0.736712 2.743067 -0.433249
C 1.695777 2.671819 -0.082802
C 0.448260 0.641265 -0.180877
O 0.527424 -0.700149 -0.367482
C -0.658769 -1.292297 -0.950201
C -1.849022 -0.988694 -0.014623
C -2.100001 0.559910 -0.165495
C -3.223174 0.965935 0.851291
C -4.506259 0.214447 0.419299
C -4.379867 -1.315569 0.315956
C -3.122971 -1.728160 -0.473544
O -1.671657 3.446526 -0.816089
C 3.014341 0.578050 0.061463
C 5.504576 -0.724514 0.339342
C 4.136085 0.989530 -0.673572
C 3.173878 -0.513542 0.935458
C 4.395981 -1.159303 1.075018
C 5.370601 0.355502 -0.536635
O 6.682936 -1.396540 0.515916
C -0.289643 -2.724982 -1.360912
C 0.206618 -3.708083 -0.293022
C -1.481834 -1.380326 1.439660
H -2.542119 0.720626 -1.161946
C -5.643327 -1.940570 -0.290232
C -3.563905 2.450907 1.035852
H 0.524193 4.345798 -0.412444
H 2.589272 3.276042 0.027378
H -0.847090 -0.752807 -1.891674
H -2.922873 0.604267 1.841955
H -5.318570 0.457763 1.117471
H -4.817309 0.615244 -0.558642
H -4.281077 -1.712039 1.336311
H -2.984418 -2.814463 -0.385440
H -3.288347 -1.522001 -1.542661
H 4.039567 1.807834 -1.382161
H 2.326142 -0.856496 1.519093
H 4.513426 -1.998093 1.754021
H 6.223512 0.691972 -1.123895
H 7.360673 -0.996991 -0.051147
H -1.152502 -3.149241 -1.888955
H 0.499841 -2.615480 -2.115237
H 0.631574 -4.593235 -0.779843
H 0.991132 -3.259146 0.323672
H -0.594871 -4.056282 0.365551
H -0.722331 -0.714871 1.858948
H -1.090478 -2.397557 1.482589
H -2.345885 -1.343708 2.104633
H -6.532933 -1.685313 0.297966
H -5.807579 -1.580845 -1.314180
H -5.569809 -3.034433 -0.328180
H -4.386847 2.529312 1.759164
H -3.872969 2.925149 0.102412
H -2.721643 3.024335 1.428209
SCF Energy = -1174.34712239
Number of imaginary frequencies = 0

Compound 3_c13
B3LYP/6-31G* Geometry
C 1.676554 1.318031 0.059166
C -0.814163 1.269192 -0.106259
N 0.413644 3.326030 0.129149
C -0.846395 2.721309 -0.037219
C 1.606306 2.676823 0.174715
C 0.413977 0.628131 -0.101459
O 0.548451 -0.711988 -0.244516
C -0.609149 -1.525680 -0.564802
C -1.844374 -1.022647 0.221406
C -2.084917 0.434526 -0.292444
C -3.440667 1.076355 0.180484
C -4.367326 0.109625 0.964096
C -4.398948 -1.380407 0.526490
C -3.093805 -1.835977 -0.174707
O -1.829608 3.465138 -0.108065
C 2.995576 0.635780 0.069294
C 5.559351 -0.547868 0.143736
C 4.060502 1.145106 -0.694727
C 3.250637 -0.491296 0.867012
C 4.512589 -1.079325 0.905017
C 5.328002 0.571924 -0.659656
O 6.819101 -1.080753 0.141163
C -0.158159 -2.969849 -0.337303
C 1.023265 -3.377130 -1.226489
C -1.556702 -1.123740 1.736237
H -2.203922 0.309416 -1.380295
C -5.617299 -1.723543 -0.344659
C -4.170693 1.684185 -1.030342
H 0.370519 4.335830 0.194554
H 2.487667 3.292442 0.314881
H -0.805485 -1.390763 -1.639062
H -3.214570 1.905940 0.852206
H -4.092997 0.150325 2.023756

H -5.386627 0.515369 0.924308
H -4.494769 -1.972779 1.446687
H -2.942859 -2.902646 0.029800
H -3.220226 -1.749058 -1.264619
H 3.885813 1.997750 -1.345666
H 2.453420 -0.911569 1.469954
H 4.686447 -1.949783 1.535760
H 6.142021 0.969174 -1.257807
H 6.836241 -1.850782 0.730496
H 0.108477 -3.104461 0.717623
H -1.008150 -3.631647 -0.537734
H 1.296222 -4.423435 -1.049504
H 0.771545 -3.274249 -2.289278
H 1.900626 -2.755606 -1.027580
H -0.575010 -0.714658 1.989440
H -1.573164 -2.170683 2.060140
H -2.294437 -0.586106 2.332066
H -6.553746 -1.505056 0.182161
H -5.616619 -1.145376 -1.276597
H -5.625379 -2.787525 -0.613961
H -5.090912 2.190073 -0.714240
H -4.448117 0.913243 -1.762026
H -3.536038 2.424086 -1.523681
SCF Energy = -1174.34655764
Number of imaginary frequencies = 0

Compound 3_c14
B3LYP/6-31G* Geometry
C 1.666401 1.323954 -0.072707
C -0.822251 1.252244 -0.227011
N 0.392529 3.325322 -0.079455
C -0.861866 2.705760 -0.235797
C 1.589713 2.685649 -0.015988
C 0.408651 0.617893 -0.193219
O 0.551435 -0.728313 -0.235173
C -0.606951 -1.583012 -0.422865
C -1.834109 -1.001055 0.319477
C -2.087862 0.397354 -0.332197
C -3.443474 1.072325 0.092602
C -4.361927 0.177416 0.966571
C -4.386727 -1.346354 0.672068
C -3.080738 -1.857498 0.014394
O -1.846734 3.439768 -0.363671
C 2.987295 0.649034 0.004062
C 5.559290 -0.509360 0.116662
C 3.396984 -0.297400 -0.948389
C 3.900706 0.993577 1.014994
C 5.172463 0.430861 1.075271
C 4.664131 -0.872921 -0.895795
O 6.816237 -1.039125 0.219161
C -0.143482 -2.983199 -0.015543
C 1.030908 -3.503469 -0.852908
C -1.529592 -0.940269 1.834267
H -2.213275 0.169808 -1.402764
C -5.603768 -1.776267 -0.161793
C -4.182557 1.573246 -1.160750
H 0.345890 4.337056 -0.089982
H 2.471524 3.311989 0.060098
H -0.826062 -1.587774 -1.501263
H -3.215847 1.958050 0.687332
H -4.083730 0.317235 2.016606
H -5.383456 0.572523 0.893299
H -4.478840 -1.849916 1.644046
H -2.920845 -2.897891 0.320645
H -3.212091 -1.878703 -1.078289
H 2.719546 -0.580005 -1.746867
H 3.600719 1.705545 1.779457
H 5.869953 0.698626 1.862693
H 4.962046 -1.599651 -1.650019
H 6.948186 -1.677317 -0.499054
H 0.134303 -2.975892 1.044488
H -0.991608 -3.669070 -0.119553
H 1.292491 -4.525097 -0.555635
H 0.778496 -3.522448 -1.920842
H 1.916224 -2.874941 -0.724124
H -0.512496 -0.586993 2.024559
H -1.622776 -1.933450 2.288489
H -2.209146 -0.271813 2.364361
H -6.541214 -1.515093 0.343338
H -5.606634 -1.287490 -1.143645
H -5.605891 -2.860744 -0.330765
H -5.101865 2.102631 -0.882690
H -4.463166 0.743416 -1.823636
H -3.553058 2.270458 -1.719077
SCF Energy = -1174.34638825
Number of imaginary frequencies = 0

Compound 3_c15
B3LYP/6-31G* Geometry
C 1.597681 1.307210 -0.087482
C -0.892948 1.142227 -0.132025
N 0.259057 3.228083 -0.486601
C -0.964900 2.536157 -0.548650
C 1.474821 2.655516 -0.268170
C 0.360733 0.554558 -0.096593
O 0.517366 -0.792279 -0.114654
C -0.616222 -1.527506 -0.636702
C -1.849077 -1.200878 0.232036
C -2.175013 0.317608 -0.003475
C -3.210133 0.763570 1.088904
C -4.351599 -0.307803 1.219722
C -4.453506 -1.372700 0.103550
C -3.076038 -2.024354 -0.229440
O -1.956613 3.136793 -0.964517
C 2.933025 0.680881 0.069094
C 5.517953 -0.407692 0.407571
C 4.017755 1.108211 -0.717954
C 3.179730 -0.319169 1.023941
C 4.451933 -0.859746 1.192508
C 5.295161 0.582906 -0.553077
O 6.788649 -0.899351 0.528694
C -0.167962 -2.985404 -0.738335
C 0.995973 -3.182639 -1.717181
C -1.494973 -1.526401 1.705394
H -2.683618 0.394686 -0.974017
C -5.167138 -0.838417 -1.152755
C -3.846747 2.154291 0.935145
H 0.189091 4.205508 -0.742951
H 2.328052 3.324250 -0.255097
H -0.803106 -1.147139 -1.654014
H -2.662780 0.778838 2.039925
H -4.251320 -0.825695 2.180737
H -5.316728 0.210157 1.275783
H -5.101814 -2.167087 0.496877
H -3.020626 -3.025034 0.216654
H -3.015946 -2.175962 -1.316567
H 3.852008 1.853866 -1.491002
H 2.367105 -0.678988 1.645474
H 4.617986 -1.631722 1.942340
H 6.124258 0.915666 -1.169615
H 6.797558 -1.579815 1.219647
H 0.122008 -3.350508 0.253256
H -1.026818 -3.586384 -1.059170
H 1.284136 -4.238339 -1.769732
H 0.720868 -2.861329 -2.729389
H 1.870618 -2.603857 -1.406969
H -0.705167 -0.875478 2.090601
H -1.142162 -2.559593 1.787868
H -2.354777 -1.435884 2.369332
H -6.165974 -0.463328 -0.899041
H -4.622151 -0.018670 -1.633151
H -5.290004 -1.634335 -1.897840
H -4.594696 2.283757 1.728564
H -4.351477 2.267049 -0.029526
H -3.124186 2.964942 1.014887
SCF Energy = -1174.34047459
Number of imaginary frequencies = 0

Compound 3_c16
B3LYP/6-31G* Geometry
C 1.595439 1.274003 -0.269103
C -0.890418 1.110702 -0.282960
N 0.260502 3.166238 -0.790906
C -0.960244 2.466428 -0.812041

C 1.476567 2.605798 -0.540660
C 0.360395 0.519474 -0.220076
O 0.518136 -0.823008 -0.111782
C -0.640814 -1.607149 -0.488980
C -1.841660 -1.163712 0.374645
C -2.172940 0.315205 -0.035154
C -3.180804 0.894883 1.020354
C -4.315402 -0.151015 1.317940
C -4.451240 -1.338396 0.339673
C -3.084460 -2.033528 0.059081
O -1.948653 3.026804 -1.287286
C 2.934175 0.678643 -0.019024
C 5.527945 -0.342352 0.418031
C 3.424053 -0.397048 -0.774950
C 3.779013 1.227424 0.960311
C 5.061638 0.732599 1.179538
C 4.702787 -0.905887 -0.561626
O 6.792121 -0.798631 0.673113
C -0.216505 -3.074105 -0.409614
C 0.887999 -3.446124 -1.405158
C -1.435195 -1.304789 1.863585
H -2.703081 0.276948 -0.996415
C -5.192015 -0.950289 -0.954177
C -3.828609 2.255095 0.710911
H 0.194378 4.112749 -1.145712
H 2.336717 3.262892 -0.604865
H -0.866322 -1.361602 -1.539559
H -2.608845 1.029829 1.946967
H -4.179616 -0.552929 2.328897
H -5.278855 0.372103 1.346190
H -5.093634 -2.076382 0.838618
H -3.019733 -2.965664 0.633886
H -3.055528 -2.329099 -0.999401
H 2.802978 -0.831051 -1.550601
H 3.415894 2.049268 1.571820
H 5.707124 1.158737 1.941108
H 5.064498 -1.737321 -1.164653
H 6.982358 -1.544065 0.082647
H 0.119188 -3.303207 0.607673
H -1.100516 -3.693357 -0.599796
H 1.111192 -4.517490 -1.351261
H 0.588387 -3.220244 -2.436272
H 1.810215 -2.899101 -1.191187
H -0.616063 -0.631345 2.129388
H -1.101106 -2.327264 2.067196
H -2.265254 -1.106920 2.541705
H -6.184971 -0.545933 -0.723247
H -4.658289 -0.191946 -1.536914
H -5.331115 -1.826253 -1.599956
H -4.566001 2.474680 1.494168
H -4.347661 2.250742 -0.252909
H -3.110915 3.073082 0.683183
SCF Energy = -1174.33891430
Number of imaginary frequencies = 0

Compound 3_c17
B3LYP/6-31G* Geometry
C 1.721649 1.308201 0.067673
C -0.769743 1.297266 -0.089288
N 0.482177 3.318030 0.298501
C -0.785137 2.739157 0.099879
C 1.667926 2.654426 0.287877
C 0.453201 0.645643 -0.147418
O 0.575533 -0.677887 -0.417313
C -0.603065 -1.402878 -0.844460
C -1.809790 -0.997202 0.038460
C -2.061325 0.503164 -0.319144
C -3.387144 1.086009 0.293937
C -4.270811 0.024947 1.002896
C -4.357810 -1.386422 0.363517
C -3.064137 -1.779165 -0.397791
O -1.763148 3.492789 0.102807
C 3.031504 0.609259 0.025314
C 5.575030 -0.616281 0.008886
C 4.090475 1.132030 -0.736847
C 3.281689 -0.550265 0.775680
C 4.533838 -1.159123 0.769899
C 5.348693 0.537157 -0.746915
O 6.825596 -1.168757 -0.036569
C -0.179514 -2.872087 -0.969959
C 0.270409 -3.614942 0.292756
C -1.484094 -1.200558 1.544229
H -2.233939 0.502333 -1.406507
C -5.588058 -1.571449 -0.538798
C -4.179210 1.831275 -0.795299
H 0.449979 4.319708 0.444946
H 2.557317 3.247207 0.470100
H -0.824492 -1.064889 -1.867136
H -3.124653 1.830104 1.048802
H -3.915850 -0.093128 2.031876
H -5.283603 0.438048 1.097614
H -4.474736 -2.093911 1.195552
H -2.899037 -2.858121 -0.287689
H -3.212745 -1.602343 -1.473993
H 3.918340 2.013431 -1.349064
H 2.489963 -0.975740 1.382937
H 4.704834 -2.054932 1.364901
H 6.159253 0.944546 -1.342942
H 6.840274 -1.960499 0.523329
H -1.005704 -3.413449 -1.446418
H 0.645555 -2.884358 -1.693519
H 0.643896 -4.608934 0.021491
H 1.082387 -3.082532 0.797641
H -0.543541 -3.753668 1.009693
H -0.406857 -1.231552 1.721430
H -1.906437 -2.137852 1.921086
H -1.881351 -0.389961 2.157964
H -6.516011 -1.390647 0.016551
H -5.569611 -0.881646 -1.390894
H -5.632795 -2.592105 -0.940006
H -5.088224 2.278558 -0.375080
H -4.484442 1.155935 -1.605574
H -3.574318 2.636033 -1.218483
SCF Energy = -1174.34156940
Number of imaginary frequencies = 0

Compound 3_c18
B3LYP/6-31G* Geometry
C 1.720638 1.309546 0.070734
C -0.770374 1.298074 -0.090727
N 0.480865 3.319058 0.299453
C -0.786147 2.739939 0.098217
C 1.666833 2.655485 0.291355
C 0.452763 0.646741 -0.146906
O 0.576748 -0.676095 -0.417131
C -0.601050 -1.403759 -0.842633
C -1.808594 -0.997013 0.038686
C -2.060975 0.502738 -0.321450
C -3.387730 1.085583 0.289438
C -4.271473 0.024829 0.998711
C -4.357069 -1.387234 0.360804
C -3.061931 -1.780574 -0.397725
O -1.764026 3.493775 0.098827
C 3.029224 0.608020 0.030663
C 5.565523 -0.631388 0.020332
C 4.083186 1.118099 -0.740717
C 3.280191 -0.545969 0.795309
C 4.525760 -1.161081 0.793781
C 5.340619 0.513757 -0.747212
O 6.770533 -1.277850 0.055617
C -0.175448 -2.872723 -0.964018
C 0.277020 -3.611171 0.300383
C -1.484128 -1.197758 1.545075
H -2.232247 0.500280 -1.409033
C -5.585641 -1.573584 -0.543519
C -4.178883 1.829400 -0.801439
H 0.447903 4.320501 0.447151
H 2.555907 3.247956 0.476278
H -0.822462 -1.068469 -1.866246
H -3.126430 1.830598 1.043822
H -3.917139 -0.092008 2.028025
H -5.284584 0.437465 1.092214
H -4.475364 -2.093736 1.193460
H -2.896157 -2.859138 -0.285238

H -3.209111 -1.605972 -1.474504
H 3.913880 1.993124 -1.362687
H 2.487659 -0.959108 1.410227
H 4.715109 -2.048110 1.390209
H 6.140269 0.926843 -1.359937
H 7.394665 -0.811344 -0.521763
H -1.001232 -3.416519 -1.438488
H 0.649128 -2.885851 -1.688135
H 0.651888 -4.605179 0.031410
H 1.089116 -3.076518 0.802512
H -0.535925 -3.749264 1.018617
H -0.407090 -1.228804 1.723324
H -1.906790 -2.134400 1.923096
H -1.881806 -0.386076 2.157140
H -6.514656 -1.392064 0.009829
H -5.565651 -0.885023 -1.396603
H -5.629532 -2.594818 -0.943341
H -5.088744 2.276456 -0.382796
H -4.482622 1.153169 -1.611523
H -3.573947 2.634202 -1.224536
SCF Energy = -1174.34155012
Number of imaginary frequencies = 0

Compound 3_c19
B3LYP/6-31G* Geometry
C 1.798338 1.281413 0.015998
C -0.693547 1.317231 -0.135745
N 0.608539 3.335273 0.021607
C -0.673053 2.770801 -0.119108
C 1.777284 2.645118 0.085990
C 0.511041 0.632671 -0.116349
O 0.596739 -0.715997 -0.218130
C -0.593332 -1.481825 -0.547049
C -1.794727 -0.926068 0.258690
C -1.998879 0.527261 -0.276694
C -3.318130 1.225675 0.220229
C -4.252874 0.307881 1.052036
C -4.347686 -1.189704 0.649242
C -3.079064 -1.708028 -0.076997
O -1.630171 3.545433 -0.212831
C 3.091692 0.552259 0.042912
C 5.610719 -0.721126 0.145552
C 4.172847 1.002591 -0.735255
C 3.306756 -0.563025 0.868384
C 4.546660 -1.194514 0.920877
C 5.418843 0.385021 -0.686598
O 6.850446 -1.299468 0.156105
C -0.192882 -2.954096 -0.350322
C -0.755582 -3.906094 -1.414134
C -1.470268 -1.004457 1.768637
H -2.152814 0.388813 -1.358418
C -5.601265 -1.509264 -0.179956
C -4.064629 1.828387 -0.983202
H 0.602280 4.347612 0.052704
H 2.680844 3.232922 0.202243
H -0.784545 -1.314673 -1.616931
H -3.044874 2.062636 0.865232
H -3.946972 0.361798 2.102325
H -5.258757 0.747216 1.031764
H -4.438494 -1.757480 1.585270
H -2.951066 -2.771476 0.158463
H -3.237126 -1.650904 -1.164569
H 4.027828 1.843933 -1.407838
H 2.495701 -0.939666 1.481524
H 4.689783 -2.054957 1.572789
H 6.245558 0.736857 -1.295682
H 6.840420 -2.052876 0.766676
H 0.901397 -2.987514 -0.393425
H -0.472184 -3.299495 0.651657
H -0.402509 -4.928087 -1.237426
H -1.849757 -3.930645 -1.417803
H -0.426672 -3.610713 -2.417723
H -0.466623 -0.633022 1.988782
H -1.523551 -2.040966 2.120818
H -2.170117 -0.422594 2.368258
H -6.514488 -1.246111 0.366780
H -5.606787 -0.953016 -1.125042
H -5.653512 -2.578196 -0.423494
H -4.958242 2.371388 -0.652586
H -4.388888 1.049378 -1.686504
H -3.421300 2.534547 -1.513065
SCF Energy = -1174.34286897
Number of imaginary frequencies = 0

Compound 3_c20
B3LYP/6-31G* Geometry
C 1.786027 1.285039 -0.141938
C -0.705858 1.299692 -0.270662
N 0.584471 3.330632 -0.224393
C -0.693602 2.751844 -0.336644
C 1.758142 2.650248 -0.141054
C 0.502034 0.623118 -0.234695
O 0.594811 -0.728644 -0.248070
C -0.599512 -1.530745 -0.454933
C -1.779149 -0.906867 0.328587
C -2.007650 0.495300 -0.320143
C -3.316087 1.221614 0.166372
C -4.208323 0.365409 1.103738
C -4.318863 -1.154155 0.808240
C -3.066324 -1.720809 0.091448
O -1.655071 3.513322 -0.480176
C 3.080045 0.565309 -0.025813
C 5.605226 -0.683856 0.170177
C 3.440305 -0.479529 -0.891911
C 4.018691 0.958498 0.944423
C 5.267423 0.352594 1.044395
C 4.683855 -1.098960 -0.797573
O 6.840830 -1.254100 0.310099
C -0.180928 -2.970439 -0.106022
C -0.737575 -4.035511 -1.059863
C -1.406422 -0.847022 1.832771
H -2.191505 0.271356 -1.382852
C -5.591156 -1.526351 0.031133
C -4.109238 1.726590 -1.051870
H 0.575257 4.342122 -0.276131
H 2.662025 3.247222 -0.094699
H -0.827124 -1.479818 -1.529636
H -3.028466 2.108154 0.734204
H -3.848359 0.485890 2.130874
H -5.214177 0.805445 1.104522
H -4.389304 -1.655307 1.783144
H -2.923415 -2.761894 0.404815
H -3.254085 -1.749547 -0.992557
H 2.742240 -0.807607 -1.653816
H 3.755900 1.744066 1.648080
H 5.983132 0.659397 1.800616
H 4.942378 -1.903547 -1.484371
H 6.938105 -1.959066 -0.348730
H 0.913521 -2.998135 -0.145501
H -0.454345 -3.207090 0.928524
H -0.373679 -5.029812 -0.778259
H -1.831552 -4.070818 -1.055531
H -0.416940 -3.844658 -2.091313
H -0.334686 -0.685223 1.973831
H -1.665976 -1.784633 2.337370
H -1.925347 -0.040942 2.353090
H -6.491446 -1.221647 0.577749
H -5.615997 -1.038382 -0.950510
H -5.651333 -2.609647 -0.134869
H -4.999681 2.281097 -0.731803
H -4.444688 0.896852 -1.688647
H -3.492665 2.401055 -1.650650
SCF Energy = -1174.34281474
Number of imaginary frequencies = 0
